# Leafy and weedy seadragon genomes connect genic and repetitive DNA features to the extravagant biology of syngnathid fishes

**DOI:** 10.1073/pnas.2119602119

**Published:** 2022-06-22

**Authors:** Clayton M. Small, Hope M. Healey, Mark C. Currey, Emily A. Beck, Julian Catchen, Angela S. P. Lin, William A. Cresko, Susan Bassham

**Affiliations:** ^a^Institute of Ecology and Evolution, University of Oregon, Eugene, OR 97403;; ^b^Presidential Initiative in Data Science, University of Oregon, Eugene, OR 97403;; ^c^Department of Evolution, Ecology, and Behavior, University of Illinois at Urbana–Champaign, Urbana, IL 61801;; ^d^Knight Campus for Accelerating Scientific Impact, University of Oregon, Eugene, OR 97403

**Keywords:** genome sequencing, novel traits, transposable elements, fibroblast growth factors, syngnathid fishes

## Abstract

Seadragons are a remarkable lineage of teleost fishes in the family Syngnathidae, renowned for having evolved male pregnancy. Comprising three known species, seadragons are widely recognized and admired for their fantastical body forms and coloration, and their specific habitat requirements have made them flagship representatives for marine conservation and natural history interests. Until recently, a gap has been the lack of significant genomic resources for seadragons. We have produced gene-annotated, chromosome-scale genome models for the leafy and weedy seadragon to advance investigations of evolutionary innovation and elaboration of morphological traits in seadragons as well as their pipefish and seahorse relatives. We identified several interesting features specific to seadragon genomes, including divergent noncoding regions near a developmental gene important for integumentary outgrowth, a high genome-wide density of repetitive DNA, and recent expansions of transposable elements and a vesicular trafficking gene family. Surprisingly, comparative analyses leveraging the seadragon genomes and additional syngnathid and outgroup genomes revealed striking, syngnathid-specific losses in the family of fibroblast growth factors (FGFs), which likely involve reorganization of highly conserved gene regulatory networks in ways that have not previously been documented in natural populations. The resources presented here serve as important tools for future evolutionary studies of developmental processes in syngnathids and hold value for conservation of the extravagant seadragons and their relatives.

## Significance

Seadragons are widely recognized for their derived traits, which include leaf-like appendages and extreme spinal curvature. Efforts to understand the genetic basis of these unique traits and conserve these species and their relatives have been limited by genomic resource gaps. In this paper we present full, annotated genomes of leafy and weedy seadragons, which we use to uncover surprising features of gene family and genome architecture evolution that likely relate to the extravagant phenotypic traits of seadragons and their pipefish and seahorse relatives. These genomes and their analyses are important advances for the study of elaborate vertebrate traits, leveraging this diverse, morphologically exceptional group of fishes.

**Fig. 1.**
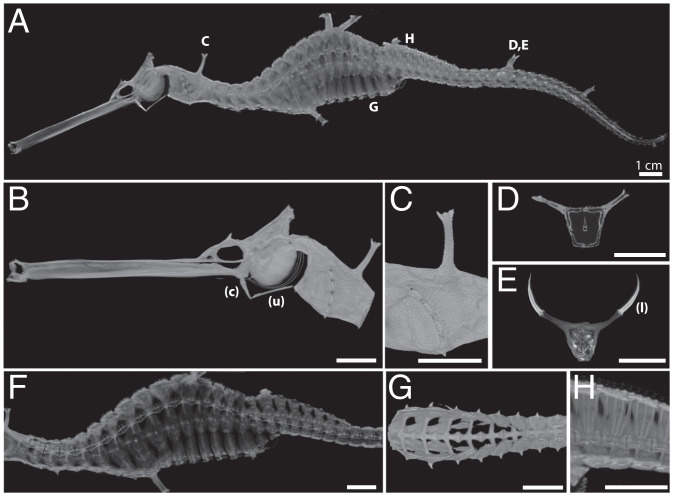
The anatomy of the weedy seadragon includes remarkably elongated facial features terminating in toothless, upturned jaws, an unusual hyoid apparatus specialized for suction feeding, a bony exoskeleton with elaborate spines that support fleshy leaves, and a sinusoidal spine of ribless vertebrae that vary in shape and size. (*A*) Lateral view of the skeleton of *P. taeniolatus* reconstructed by X-ray microscopy. (*B*) Detail of the head (the ceratohyal, c, and urohyal, u, of the hyoid apparatus are noted). (*C*) Detail of the pectoral region (lateral view) showing a dorsal, unpaired “leafy” appendage support surrounded by other dermal plates with much shorter spines. (*D*) Optical cross-section of the tail through a pair of leafy appendage spines. (*E*) Optical cross-section through the same appendages as in *D* but with a contrast agent that reveals the fleshy leaves, as denoted by (l). (*F*) Lateral view shows keystone-shaped vertebrae at curvatures—both kyphosis and lordosis—of the spine. (*G*) Ventral view of the ribless abdominal vertebrae. (*H*) Lateral detail of the specialized vertebrae beneath the propulsive dorsal fin.

Seadragons are phenotypic outliers in an already exceptional clade of teleost fishes (family Syngnathidae) that also includes seahorses and pipefishes. For this reason, seadragons are often a colorful, flagship group in discussions of adaptation and evolutionary innovation. They show strikingly derived characters compared to their pipefish and seahorse relatives, including “leafy appendages,” extreme curvature of the spine (kyphosis and lordosis), elongated craniofacial bones, and large body size (Fig. 1) (1, 2). Substantial differences exist even among the three known extant species: *Phycodurus eques* (leafy seadragon), *Phyllopteryx taeniolatus* (weedy, or common, seadragon), and the recently described *Phyllopteryx dewysea* (ruby seadragon) (2).

In addition to being a focus for evolutionary studies, seadragons are of significant cultural and conservation interest (3–5). Presumed adaptations for crypsis, including the leafy appendages, unique body plan, and elaborate skin coloration, contribute to the status of seadragons as distinguished and valued cultural symbols for the people of Australia, where seadragon species are endemic. Because seadragon distributions are specific to temperate Australian macroalgal reefs, and their population sizes are relatively small, seadragons are likely susceptible to negative human impacts, including global climate change. Furthermore, recent population genomic studies documenting significant population structure (6, 7) in these species are especially relevant to conservation decisions. The unique evolutionary innovations, cultural importance, and conservation challenges all elevate the need to better understand and conserve seadragon species.

To improve our understanding of highly derived phenotypic traits and genomic features within seadragons, as well as those that are shared but derived among the Syngnathidae, we created annotated, chromosome-scale assemblies for a male leafy seadragon and a female weedy seadragon. In addition to the production of these resources, we carried out several comparative analyses among five syngnathid and many other teleost genomes to determine changes in genome organization and content, including a detailed analysis of key gene families and regulatory elements that may be involved in the development of syngnathid innovations. Lastly, we performed high-resolution three-dimensional (3D) X-ray microscope scans of an adult male weedy seadragon to more precisely view seadragon innovations.

Our work reveals several seadragon-specific genomic features, including divergent conserved noncoding elements (CNEs) near key developmental genes, a unique microRNA gene repertoire, and expanded gene families related to immunity and vesicular trafficking. We also found that the seadragon genomes are highly repetitive for their sizes, with unique repeat abundance distributions. Because the seadragon lineage occupies a region of the syngnathid phylogeny that is relatively basal to most of the species’ diversity, we leveraged their phylogenetic position to identify several genomic synampomorphies of the family. These genomic features include the striking loss of several highly conserved fibroblast growth factor (FGF) genes, expansions and contractions of gene families related to immunity and potentially male pregnancy, and syngnathid-specific transposable element (TE) expansion.

With these genome models and rich accompanying data, we add to the existing collection of high-quality genomic tools and insights for several syngnathid groups, including genera *Syngnathus* (8, 9), *Hippocampus* (10, 11), *Microphis* (12), and most recently (published as of the writing of this paper) *Phyllopteryx* and *Syngnathoides* (13). Such tools are useful in illuminating the evolution and development of puzzling syngnathid novelties such as male pregnancy and leaf-like appendages. These genomic resources will also support ongoing efforts to understand and conserve sensitive syngnathid populations, including phylogenetic umbrella species like the seadragons.

## Materials and Methods

### Seadragon Genome Assemblies.

We isolated high molecular weight genomic DNA from tissues of an adult male leafy seadragon (*P. eques*) and from an adult female common (“weedy”) seadragon (*P. taeniolatus*). We then generated PacBio libraries for both species and sequenced 49.12 and 80.80 Gb, respectively. We also generated “shotgun” whole-genome sequencing (WGS) Illumina libraries for both species, sequencing 57.48 (*P. eques*) and 105.79 Gb (*P. taeniolatus*), to estimate genome size and polish the PacBio assemblies (see *SI Appendix*, *SI Methods* for all software versions and parameters). We assembled both genomes with Flye (14), using all PacBio data excluding “scraps” and an estimated genome size of 600 Mb, followed by two rounds of polishing with the tool arrow (15). We performed an additional two rounds of polishing per genome with WGS Illumina data, using pilon (16). To organize Flye assemblies into putative chromosome-scale genome models we generated Hi-C libraries using Phase Genomics Proximo Animal kits, then scaffolded using the 3D-DNA pipeline (17) with breaking of original scaffolds disabled, aided by Juicer and Juicebox (18, 19). We evaluated assembly quality and completeness using Quast (20) and BUSCO (21).

### Draft Short-Read Genome Assemblies for *Doryrhamphus excisus* and *Synchiropus splendidus*.

To supplement our comparative analyses with additional syngnathid genomes and a close outgroup we produced linked-reads assemblies for the bluestripe pipefish (*D. excisus*) and the Mandarin dragonet (*S. splendidus*). The *D. excisus* assembly was used in all subsequent comparative genomic analyses, and the *S. splendidus* assembly was used in all except the repetitive DNA analyses, in which cases the chromosome-level assembly for a different dragonet, *Callionymus lyra* was used to maximize completeness (22). These assemblies were performed using 10× Genomics Chromium technology and the Supernova assembly software (23), as described in Stervander and Cresko (24).

### mRNA Sequencing (mRNA-Seq).

To generate mRNA-seq libraries, we extracted total RNA from tissues of the same *P. eques* and *P. taeniolatus* individuals as were used for PacBio genome sequencing. From the *P. eques* specimen, we dissected testis, leafy appendage, eye, and gill tissues. From *P. taenoiolatus* we dissected ovary, leafy appendage, eye, and liver tissue (*SI Appendix*, *SI Methods*). We used the Roche KAPA HyperPrep Kit to generate indexed, stranded mRNA-seq libraries for Illumina sequencing to obtain 301.52 million paired-end 100-bp reads. We trimmed Illumina adaptors and low-quality regions from reads using process_shortreads from the Stacks software suite (25, 26) and aligned cleaned RNA-seq reads from both seadragon species to both *P. taeniolatus* and *P. eques* genome assemblies using STAR aligner (27).

### miRNA-Seq.

To generate small RNA-seq reads we purified RNA from each tissue above using Zymo DirectZol columns and generated indexed sequencing libraries using the NextFlex Small RNA-Seq Kit v3. We ran BBMap (28) to align reads to both genomes. We annotated miRNAs in the leafy seadragon based on sequence conservation with annotated miRNAs from seven ray-finned fish species, as described in the Prost! (29) manual (*SI Appendix*, *SI Methods*). To run Prost! we supplied threespine stickleback noncoding sequences (29). We ran Prost! for the weedy seadragon by adding the leafy seadragon’s annotations to the species pool. We distinguished orthologous miRNAs for the seadragon species using synolog (30) synteny software, which aligned the leafy seadragon genome with the stickleback genome. Any miRNAs not identified from reads were searched for in the leafy seadragon genome with BLASTN and VISTA plots.

### Genome Annotation.

To better standardize comparisons, we generated annotations for leafy seadragon (*P. eques*), weedy seadragon (*P. taeniolatus*), greater pipefish (*Syngnathus acus*), bluestripe pipefish (*D. excisus*), Mandarin dragonet (*S. splendidus*), and Pacific bluefin tuna (31) (*Thunnus orientalis*) (see *SI Appendix*, *SI Methods* for public data accessions). Assemblies were soft-masked for repeats and areas of low complexity with RepeatMasker (31) using custom repeat libraries made by combining a teleost library extracted from RepeatModeler2 (32) with species-specific repeat libraries produced by running RepeatModeler. We aligned all RNA-seq data (including new and previously published reads) as described above and supplied .bam files to BRAKER2 (33) for annotation, followed by filtering with InterProScan (34).

### Repeat Annotation.

We characterized the repetitive content of 16 teleost genome assemblies (see *SI Appendix*, Fig. S1 and *SI Methods* for accession Nos.). With one exception (tiger tail seahorse [*Hippocampus comes*]), we focused exclusively on assemblies produced by long-read (i.e., PacBio or Oxford Nanopore) and/or linked-read (i.e., 10× Genomics) technologies. All genomes were subject to a unified repeat library generation and annotation workflow. We identified repeats de novo for each assembly using: 1) RepeatModeler2 (32) and 2) TransposonPSI (35). We combined those repeat predictions with teleost repeats extracted from RepeatMasker (31) libraries and all sequences from the FishTEDB database (36) (http://www.fishtedb.org). These sequences (576,007 in total) were classified using the RepeatClassifier module of RepeatModeler. We also clustered repeats at 80% sequence identity using USEARCH (37) as a way to group and ultimately enumerate repeats based on sequence divergence (*SI Appendix*, *SI Methods*). We ran RepeatMasker on all 16 genome assemblies using all 576,007 sequences and integrated the aforementioned RepeatClassifier and USEARCH cluster IDs into the RepeatMasker output. Finally, we used RepeatClassifier taxonomy and USEARCH cluster membership as alternative grouping mechanisms to characterize the distribution of repeats within and among genomes, and to ordinate genomes in repeat space using principal components analysis (PCA ). Regional repeat abundance distributions for target gene families (*SI Appendix*, *SI Methods*) were compared to those from random samples of genes across the genome using resampling-based hypothesis tests. All downstream repeat analysis and visualizations were carried out using the R statistical language (38).

### Gene Family Evolution.

**Fig. 2.**
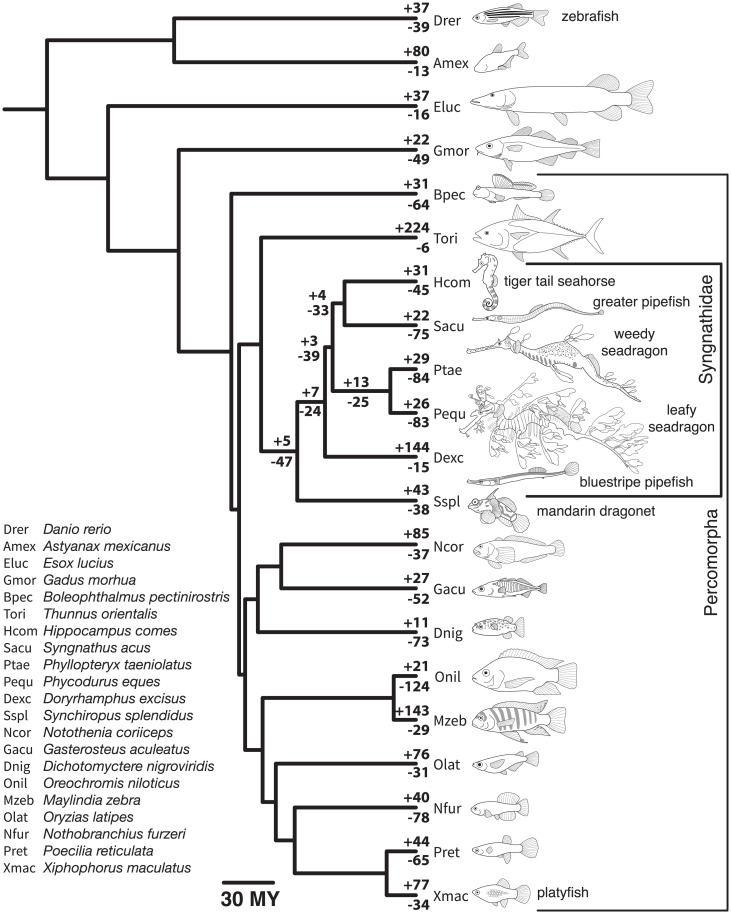
Genomes of 21 teleost species provide phylogenetic context for gene family evolution in seadragons and syngnathid fishes. Represented are evolutionary relationships among a sample of morphologically diverse teleosts, according to a time-calibrated phylogenetic tree adapted from Rabosky et al. (120). We used published and newly generated protein-coding gene annotations for the species pictured here to understand putative gene family expansions and contractions in lineages of interest, specifically the seadragons. The number of gene families with statistical evidence for expansion (*Top* values) and contraction (*Bottom* values) along all terminal branches and internal syngnathiform branches are shown. Note that the four-letter species symbols in the legend are used in this article and its supplementary files.

We used protein annotations from 21 teleost genomes (Fig. 2 and see *SI Appendix*, *SI Methods* for accession Nos.) to better understand gene family size evolution in seadragon and syngnathid lineages. We defined putative gene families via all-by-all blastp (39) and clustering with mcl (40), then we conducted a series of gene family size evolution analyses using CAFE 5 (41). Briefly, we first fit an error model to account for artifactual (e.g., genome assembly or annotation error) family size variation, which was applied to subsequent CAFE runs. We fit a model assuming a single rate of gene family expansion/contraction (λ) to the data and identified gene families evolving especially rapidly using CAFE’s internal likelihood ratio tests (LRTs).

Of those families showing evidence for rapid evolution, we identified subsets for which branch-specific LRTs suggested extreme λs along the terminal *P. eques* and *P. taeniolatus* branches, the internal seadragon branch, and the internal syngnathid branch. We assigned each family to a Kyoto Encyclopedia of Genes and Genomes (KEGG) orthology (KO) identifier if possible, to perform KEGG pathway overrepresentation analysis using ClusterProfiler (42), with respect to the branch-specific rapidly evolving gene families. Lastly, we fit several multiple-λ models to the data in order to test hypotheses of overall differences (from a global λ) for the internal seadragon branch and the internal syngnathid branch. To test these hypotheses, we used CAFE to perform 100 simulations, fit the aforementioned models to the simulated datasets, and compared likelihood ratios (LRs) from the data to the LR distributions from the simulations. For details, see the *SI Appendix*, *SI Methods*.

### *fgf* and *fgfr* Gene Family Characterization.

We collected fibroblast growth factor (FGF) and receptor (FGFR) amino acid sequences from Ensembl of several percomorph species (*SI Appendix*, *SI Methods*). We aligned orthologs and screened the genome assemblies of syngnathids and outgroups based on hidden Markov model (HMM) profiles from the alignments, according to methods described in Small et al. (8). In some cases, we supplemented these sensitive searches with regional RNA-seq read alignments to correctly define exon boundaries. We conducted targeted, lineage-specific tests of positive selection using branch site models in PAML (*SI Appendix*, *SI Methods*) and tested for deleterious mutations using Provean (43).

### X-Ray Tomography and 3D Model Reconstruction.

We obtained a killed adult male weedy seadragon, fixed in neutral buffered formalin from the Tennessee Aquarium (Chattanooga, TN), and scanned it using a resolution of 54-µm voxels on a Zeiss XRadia 620 Versa X-ray microscope. The anterior of the fish, including the head and cleithrum, were scanned again at 17-µm voxel resolution. Composite virtual three-dimensional reconstructions of the unstained specimen were generated using Dragonfly Pro and Dragonfly software (Object Research Systems). After completing scans of the unstained specimen, which allowed high-contrast visualization of electron-dense bony structures, the fish was then dehydrated through an ethanol series, stained with an iodine-based X-ray contrast agent to enhance imaging of soft tissues, and a section of the rostral part of the stained tail that includes a pair of leafy appendages was scanned at 27-µm voxel resolution (Fig. 1 and *SI Appendix*, Fig. S2 and *SI Methods*).

## Results

### PacBio Assembly with Hi-C Scaffolding Yields Chromosome-Level Genome Models for the Leafy and Weedy Seadragon.

We estimated the haploid genome sizes for leafy and weedy seadragons to be 644.0 Mb and 597.3 Mb, respectively, based on k-mer frequency analysis of Illumina WGS data (44). From this analysis we also estimated genome-wide heterozygosity for each individual to be 0.27 and 0.33%. Polished PacBio assemblies were 664.24 Mb for leafy and 650.38 Mb for weedy seadragon. Before scaffolding using Hi-C data, Flye scaffold/contig N50s were 19.59/16.32 Mb (leafy) and 9.90/9.73 Mb (weedy). The longest Flye scaffolds were 38.02 Mb (leafy) and 29.83 Mb (weedy), and BUSCO completeness frequencies were 95% for both genomes. After scaffolding both polished PacBio assemblies using *P. eques* Hi-C reads, we obtained 23 putative chromosome models for each genome (*SI Appendix*, Fig. S3), which reflect 93.22% and 96.10% of the total length for final leafy and weedy seadragon genome models. These assembly and completeness metrics, our ability to annotate 22,256 and 22,043 protein-coding genes in the respective genomes, and extensive evidence for conserved synteny between the two seadragon assemblies (*SI Appendix*, Fig. S4), all support that these genome models are at least equal in quality and contiguity to recently published, chromosome-level syngnathid genomes (11, 13).

### Seadragon Karyotypes Are Conserved Relative to Other Syngnathid Genomes but Lack One of Two Chromosome Fusions Observed in *Syngnathus* and *Hippocampus*.

Haploid chromosome number in syngnathid fishes, as assessed by karyotyping and genetic mapping, is reported to be 22 or 24 in seahorse (*Hippocampus*) species (45, 46) and 22 in Gulf pipefish (*Syngnathus scovelli*) (8). In Gulf pipefish, the reduction in chromosome number from 24, the putative ancestral number in ray-finned fishes (47) to 22, likely resulted from fusion of two pairs of chromosomes orthologous to chromosomes 1 and 24 and to chromosomes 14 and 23 in platyfish (*Xiphophorus maculatus*) (8). Though a haploid number of 24 chromosomes in seahorse was reported (with some published confusion about the pertinent species) (45, 46), the genome for tiger tail seahorse (*Hippocampus comes*) (10) provides conserved synteny evidence that both ancestral chromosome fusions were already present in the common ancestor of seahorses and *Syngnathus* pipefish (*SI Appendix*, Fig. S5). As stated, our inferred seadragon haploid chromosome number of 23 is based on the size distribution of Hi-C scaffolds, which shows a sharp dropoff in scaffold length after the longest 23 scaffolds (*SI Appendix*, Fig. S3). The seadragon lineage apparently shares only the ancestral chromosome 1 to chromosome 24 fusion with the seahorse + *Syngnathus* pipefish ancestor, leaving one-to-one orthologs of platyfish chromosomes 14 and 23 (*SI Appendix*, Fig. S5).

### Seadragon Genomes Are Highly Repetitive for Their Compact Size, with Large Contributions from Relatively Recent TE Expansions.

A correlation between eukaryotic genome size and TE content has led to an appreciation that TEs can be an important driver of genome size evolution (48). The relationship is particularly apparent among some of the well-characterized genomes of teleost fish model species. Genome size and TE proportion share a positive, tightly linear relationship in a comparison of green spotted puffer (*Dichotomyctere nigroviridis*, syn. *Tetraodon nigroviridis*), threespine stickleback (*Gasterosteus aculeatus*), medaka (*Oryzias latipes*), and zebrafish (*Danio rerio*), whose genomes span a range from roughly 358 Mb to over 1.37 Gb (49). *Syngnathus* pipefish have genomes that rival green spotted puffer in genomic compactness, with assembly lengths of 307.0 Mb for the Gulf pipefish (8) (*S. scovelli*), and 324.33 Mb for the greater pipefish (*S. acus*) (RefSeq Genome GCF_901709675.1). In the spectrum of vertebrate genomes, known seahorse genomes are also diminutive, estimated at 421 Mb for the lined seahorse (*Hippocampus erectus*) (11) and 494 Mb for the tiger tail seahorse (*H. comes*) (10).

Our final genome assembly sizes for seadragons were 666.5 Mb for leafy seadragon and 652.2 Mb for weedy seadragon, which are appreciably larger than those of *Syngnathus* and *Hippocampus* but still much smaller than many other teleost genomes. Surprisingly, as much as 58.7% of the leafy and 57.9% of the weedy seadragon genome is composed of repetitive sequences, as classified by our workflow (*Materials and Methods*). To assess whether seadragons or syngnathids are exceptional in their repetitive DNA characteristics, we measured the proportion of repetitive sequence in 16 teleost genomes (*SI Appendix*, Fig. S1) using an in-common repeat reference library to create a standardized basis for comparison. Species were chosen for taxonomic breadth and having genomes assembled from long- or linked-read sequence data (except tiger tail seahorse). Genomes of five syngnathids were represented, including a seahorse and tail-brooding *Syngnathus* pipefish, the seadragons, and a basal lineage relative to these, the abdominal-brooding bluestripe pipefish (*D. excisus*).

**Fig. 3.**
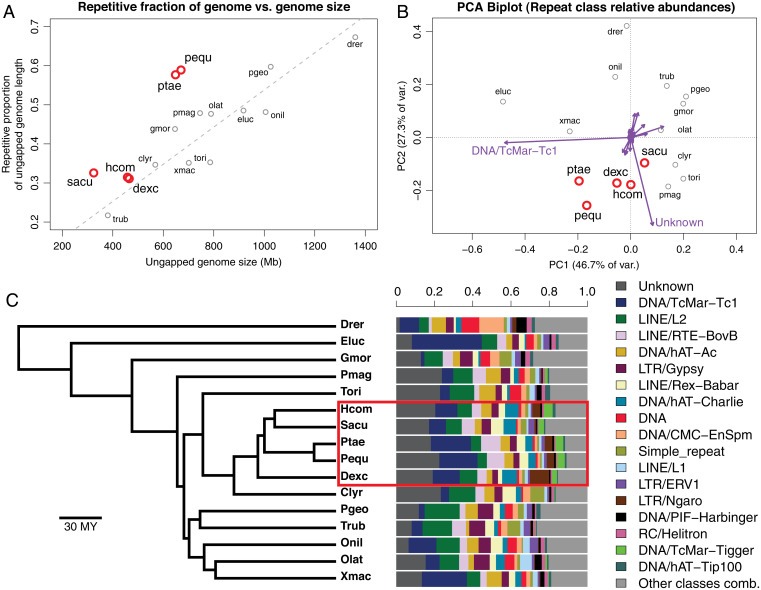
Seadragon genomes demonstrate a large fraction of repetitive DNA relative to other teleost fishes, characterized by substantial contributions from TcMar-Tc1 transposons and unclassified constituents. (*A*) Scatterplot showing the strong, positive relationship between genome assembly size (*x* axis) and the proportion of the genome annotated as repetitive for 16 recent teleost genome assemblies. Note that seadragon genomes (pequ and ptae) are especially repetitive (∼60%) given their relatively small size (∼650 Mb). Dashed line shows a PGLS regression fit, and syngnathid genomes are in red. (*B*) PCA biplot shows similarity of the 16 genomes based on relative frequencies of repeat classes. TcMar-Tc1 and “unknown” repeat classes load especially heavily on PC1 and PC2, respectively, as indicated by vectors (purple arrows) drawn in the space, and these contribute to the distinctiveness of syngnathid genomes (in red). (*C*) Barplots showing the relative abundances of repeat classes across the 16 genomes, ordered (from *Left* to *Right*, and *Top* to *Bottom* in the legend) from highest to lowest mean relative abundance. Phylogenetic relationships among the 16 species are presented as a time-calibrated tree from Rabosky et al. (120) and the syngnathid clade is indicated by a red rectangle.

We confirmed a strong, positive relationship between repeat content and genome size for the 16 genomes using phylogenetic generalized least squares (PGLS) regression (*t*_16,14_ = 4.88; *P* = 0.0002; *b* = 0.00048), but seadragons are notable outliers with large, positive residuals (Fig. 3). Both seadragon genomes are unusually bloated with repetitive DNA among teleosts for their relatively compact size, a feature either derived specifically in the seadragon lineage or an ancestral lineage. These alternatives are not yet testable, given currently available genome assemblies, but can be addressed with the addition of syngnathid genomes at key phylogenetic positions.

**Fig. 4.**
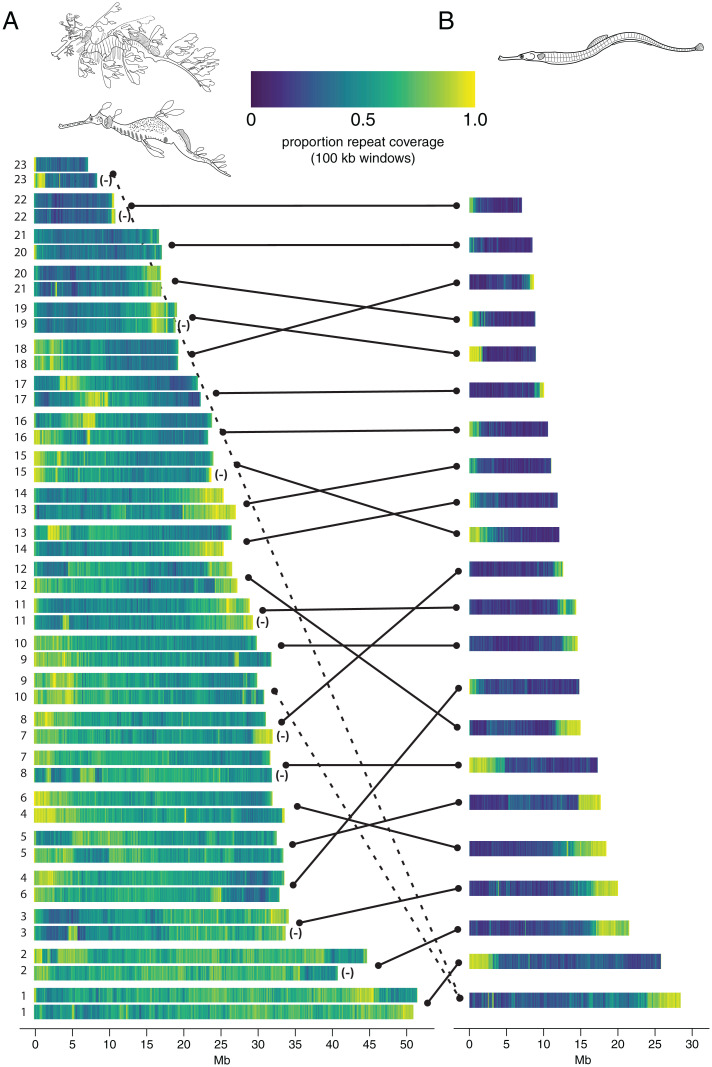
Leafy and weedy seadragon chromosomes are densely and nonuniformly populated by repetitive DNA. (*A*) Orthologous pairs of the 23 seadragon chromosomes, ordered ascending from shortest to longest leafy seadragon sequence. For each pair the leafy seadragon chromosome is on *Top*, and the weedy seadragon ortholog is *Below*. Cases in which the orientation of the weedy seadragon chromosome has been reversed to align with the leafy ortholog are denoted by “(−)”. (*B*) Chromosome models of the greater pipefish (*S. acus*), also ascending from shortest to longest. Lines connect seadragon and pipefish chromosomes with large regions of orthology, as inferred via conserved synteny analysis. Dashed lines reflect the fusion of two ancestral syngnathid chromosomes that is derived in the lineage leading to *Syngnathus* and *Hippocampus*. Overall repeat base pair occupation of 100-kb windows (expressed as a proportion) is presented as a heatmap.

Repeat density was high across seadragon chromosomes, in stark contrast with the greater pipefish (Fig. 4), which shows “hotspots” almost exclusively near chromosome ends, and a close outgroup to syngnathids, common dragonet (*C. lyra*), which has uniform, low repeat density (*SI Appendix*, Fig. S6).

Several TE classes contribute notably to the large repeatomes of seadragons. The *Tc1* family of the *Tc1/Mariner* superfamily of transposases is a major contributor to repeatome composition variation among teleosts as revealed by PCA based on within-repeatome relative class proportions, with seadragons, platyfish, and Northern pike (*Esox lucius*) genomes influenced heavily by abundant *Tc1* repeats (Fig. 3). Phylogenetic patterns of repeat abundance among the fish lineages we analyzed suggest *Tc1* expansion in the syngnathid lineage, given lower *Tc1* abundances in the close outgroups of common dragonet and Pacific bluefin tuna (*T. orientalis*). Among syngnathids, *Tc1* repeats compose a disproportionately large fraction of seadragon repeatomes (Fig. 3). This class of “cut-and-paste” DNA transposons is widespread in animals and especially common in teleost fishes, with high abundance and variability among species (49, 50). In fact, phylogenetic evidence suggests *Tc1* transposons are still active and recently expanding in some neoteleosts, such as threespine stickleback (50).

The second most abundant, classifiable TE category in seadragons was the *BovB* family of non-long terminal repeat (non-LTR) long interspersed elements (LINE) retrotransposons (Fig. 3 and *SI Appendix*, Fig. S7). This TE family is restricted to animal taxa, where its members are patchily distributed among lineages and have been inferred to populate animal genomes through horizontal transfer, perhaps via metazoan parasites (51, 52). *BovB* density variation along seadragon chromosomes (*SI Appendix*, Fig. S7) was largely concordant with overall repeat density patterns (*SI Appendix*, Fig. S6), suggesting common mechanisms or constraints for the expansion of at least some TE families.

We also discovered an apparent expansion of *Tigger* transposases in the syngnathid clade, which are members of the *pogo* superfamily closely related to *Tc1/Mariner* (53). *Tigger* repeats are overrepresented in syngnathids compared to outgroups but proportionally are conserved among the five syngnathid genomes we analyzed (Fig. 3). Unlike *Tc1* and *BovB*, *Tigger* repeat density variation along seadragon and *Syngnathus* pipefish chromosomes deviates from the collective pattern of repeat density (*SI Appendix*, Fig. S8), with the highest-density *Tigger* regions more centrally located, although the potential significance of this is unclear.

**Fig. 5.**
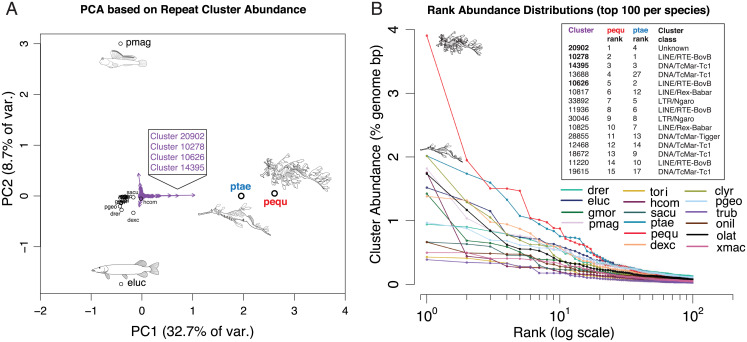
Relatively recent repeat expansions in the seadragon lineage drive the uniqueness of seadragon repeatomes. Repeat clusters defined at the 80% sequence identity level and quantified by the proportion of total genome length they occupy were used to conduct PCA. (*A*) Biplot of the first two PCA axes, showing extreme separation of seadragons from the other 14 species in repeatome space, particularly along the leading axis of variation (*x* axis). Purple arrows represent individual repeat clusters and the strength and direction by which they influence the position of genomes in this repeatome space. Four repeat clusters (shown in a box) with especially large loadings on PC1 are strongly associated with seadragon repeatome uniqueness. (*B*) Rank abundance distributions for the top 100 repeat clusters in each of the 16 species included in the repeat analysis. The top 20 clusters for each seadragon species are consistently elevated in abundance, relative to corresponding ranks in the other 14 fish genomes. Shown in a box are the top 15 leafy seadragon clusters, their ranks in weedy seadragon, and the repeat class to which they likely belong. Note that the top 3 (and fifth) clusters in the list correspond to the boxed clusters in *A*.

Because the grouping and enumeration of repeats according to classification alone comes with a loss of evolutionary resolution and precision, we also analyzed repeats as clusters of sequences with ≥80% sequence identity. PCA based on this clusterwise treatment of repeats revealed that leafy and weedy seadragon genomes are quite divergent in repeat space from the other teleost species, including the three other syngnathids compared (Fig. 5 and *SI Appendix*, Fig. S9). In particular, four repeat clusters are heavily influential, one of which was not classifiable but especially abundant in scaffolds unassigned to chromosomes (cluster 20902), two of which belong to the *BovB* LINEs mentioned above (clusters 10278 and 10626), and the last (cluster 14395) belonging to the *Tc1* group of transposases mentioned above (Fig. 5 and Dataset S1).

We also generated rank abundance distributions (RADs) for repeat clusters in the 16 fish genomes, an approach from ecology used to understand community evenness and major and rare constituents (54). Interestingly, leafy and weedy seadragon RADs show markedly high repeat cluster abundances relative to the other genomes for repeat ranks 1 to 20 (Fig. 5). Among the highest-ranking repeats for both seadragon species are the four repeat clusters mentioned above as major contributors to the distinctiveness of seadragon repeatomes. These findings, along with the observation that members of the top-ranking seadragon repeat clusters are rare in the other genomes (Dataset S1), suggest that the unique repetitive features of seadragon genomes are driven largely by recent expansions of *BovB* and *Tc1*, and a yet-to-be-classified cluster.

### Gene Family Contractions in Syngnathidae Involve Innate Immunity, and Seadragon-Specific Expansions Are Associated with Vesicular Trafficking.

We identified 290 total gene families as having expanded or contracted at a rate (λ) significantly higher than the background λ among 21 teleost species. Of these, 109 showed evidence for rapid size evolution along the leafy seadragon branch, 113 along the weedy seadragon branch, 38 along the internal seadragon branch, and 31 along the internal branch leading to syngnathids (Fig. 2 and Dataset S2). Based on 100 simulations of gene family evolution along the tree, we also inferred that λs for the internal seadragon and syngnathid branches are respectively distinct from the global λ for the tree, and likely distinct from one another (*SI Appendix*, Fig. S10).

Among the gene families most likely to have evolved rapidly in size in the ancestral syngnathid lineage, at least seven families related to innate immunity experienced contractions (Dataset S2), in contrast to multiple lines of evidence for expansion of inflammation and innate immunity gene families in teleosts relative to other vertebrates (55–59). Specifically, we found evidence for syngnathid-specific contractions in TRIM, IAN, and mannose receptor (MRC) gene families, consistent with some of the immunity and detoxification pathway gene families depleted in the genome of the Manado pipefish *(Microphis manadensis*) (12).

Several functional categories (KEGG pathways) were overrepresented among gene families with large size changes in the seadragon lineage, including cancer, cardiomyopathy, and immunity (Dataset S2), primarily due to contraction events. However, two families with roles in vesicular trafficking—*Vacuolar Protein Sorting-Associated Protein 13B* (*vps13b*) and *Coatomer Protein Complex Subunit Beta 2* (*copb2*)—show notable expansion along the branch leading to seadragons. Sequences with high similarity to *copb2*, which encodes one subunit of a Golgi budding and vesicular trafficking protein complex (60), are especially abundant in seadragon genomes relative to other syngnathids and teleosts (Dataset S2).

Though Pacific bluefin tuna and platyfish, for example, have two paralogs of *copb2*, we could find evidence for only a single gene copy in the genomes of tiger tail seahorse and greater pipefish. By contrast, we detected the presence of at least nine copies in the leafy and six copies in the weedy seadragon genomes (based on consideration of long Hi-C scaffolds with many gene annotations) (Dataset S3). We also found numerous sequences matching *copb2* on multiple short scaffolds with few other annotated genes, suggesting that the repetitive nature of this region prevented these from being incorporated into chromosome-scale scaffolds, or, but less likely, that they could be redundant artifacts. Regardless, these many, high-scoring hits specific to seadragon genomes (*SI Appendix*, Fig. S11) reflect a likely large, relatively recent *copb2* expansion. One of the *copb2* paralogs (on Pequ Hi-C scaffold 13 and Ptae Hi-C scaffold 14) appears to be the ortholog of platyfish *copb2* on the orthologous chromosome (Xmac 6), with the remaining copies likely expanding secondarily via an unknown mechanism.

Given the repetitive nature of these regions, we hypothesized that TE activity could have played a role in seadragon *copb2* expansion. Specifically, we first tested whether the seadragon-expanded TE classes of *BovB* and *Tc1* are overrepresented in the immediate vicinity (1-kb flanking both sides) of *copb2* copies, relative to 499 randomly resampled gene groups. Both *BovB* (4.24-fold enriched; *P* < 0.002) and *Tc1* (1.39-fold enriched; *P* < 0.002) were significantly overrepresented in *copb2* regions (*SI Appendix*, Fig. S11 and Dataset S4). We secondarily performed naïve hypothesis tests with the false discovery rate (FDR) controlled at 0.1 to identify other TE classes with enrichment in these regions, revealing *Rex-Babar* LINEs, and *Tigger* and *Charlie* transposons as candidates (Dataset S4).

To assess whether neighboring repeat density might be generally elevated for expanded and contracted gene families, we performed similar analyses for a reference panel of 20 families, including 5 seadragon-expanded gene families, as well as 5 seadragon-contracted families and 10 families expanded or contracted in nonsyngnathid lineages (*SI Appendix*, *SI Methods*). In addition to enrichment of *BovB* repeats in *copb2* regions, we found evidence for enrichment of this specific repeat class in one seadragon-expanded family of mucin like sequences (family 311; 2.58-fold enriched; *P* = 0.004), but in none of the remaining 18 families (Dataset S4). We also found evidence for enrichment of total repeat density in several of these 20 families. Specifically, we noted significant (FDR = 0.1) enrichment in 4 out of 5 seadragon-expanded, 1 out of 5 seadragon-contracted, and 5 out of 10 rapidly evolving (in nonsyngnathid lineages) gene families (Dataset S4). Last, we compared the repeat density among these three groups directly, treating within-family variation as a random effect. Average density was 1.00 (SEM = 0.104) for seadragon-expanded families, 0.756 (SEM = 0.080) for seadragon-contracted families, and 0.816 (SEM = 0.097) for rapidly evolving gene families in nonsyngnathid lineages, suggesting minor or no group differences (Kenward–Roger *F* test, *F*_1,2_ = 3.49; *P* = 0.057) given this sample.

### Syngnathid Fishes Have Lost Several FGF Family Genes, Most Notably *fgf3* and *fgf4*.

The FGF and FGFR gene families include well-studied ligand and receptor signaling molecules central to vertebrate craniofacial, limb, dermal appendage, sensory placode, and hindbrain development, among many other functions (reviewed in ref. 61). Given prominence of FGF signaling in the morphogenesis of traits that are distinctively modified in syngnathids, we explored whether FGF ligands or their receptors are exceptional in seadragons and other syngnathids: Flagtail pipefishes (the most basal lineage of the four), seahorses, and *Syngnathus* pipefishes (Fig. 2).

FGFs and FGFRs in seadragons and other syngnathid fishes conform, with two notable exceptions mentioned below, to a typical complement of gene paralogs relative to other percomorphs. Within Syngnathidae, there are several lineage-specific losses of FGF and FGFR genes (*SI Appendix*, Table S1). We did not detect *fgf9* in any syngnathid. Seadragons and *Syngnathus* pipefish have apparently lost the gene *fgf4-like*, while tiger tail seahorse and bluestripe pipefish have retained it. *fgf17* is missing in birds (62), in seadragons, and in bluestripe pipefish, but is present in tiger tail seahorse and greater pipefish. Because flagtail pipefishes, the lineage to which bluestripe pipefish belongs, are basal to the clade containing seadragons, seahorses, and *Syngnathus* pipefish (63), *fgf17* therefore must have been lost at least twice. Evolutionary loss of *fgf17*, though uncommon, is not unique to syngnathid lineages; orthologs of this gene have been independently erased in distant taxa, such as the medaka genus *Oryzias* (64). Also, at least two clades of *fgf5* and *fgfr1b* are present in seadragons, tiger tail seahorse, and bluestripe pipefish, but absent in the two *Syngnathus* pipefish genomes (*S. acus* and *S. scovelli*).

**Fig. 6.**
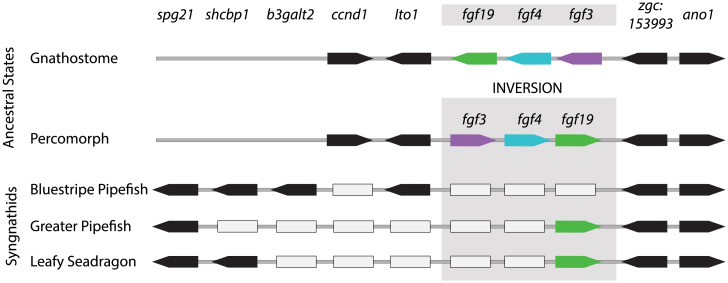
The *fgf3/4/19* cluster locus has experienced surprising gene losses in the syngnathid lineage. While retaining the same immediate gene neighbors, the FGF cluster became inverted in the percomorph fishes relative to outgroups like zebrafish and tetrapods. Several genes (gray rectangles) appear to have been deleted from the locus in the syngnathids, though not all of the losses are shared. *spg21*, *shcbp1*, and *b3galt2*, which neighbor the locus in the syngnathid lineage, are separated from the FGF cluster by other genes in nonsyngnathid percomorphs like platyfish. Genes that appear lost from the locus cannot be found anywhere else in the genome assemblies of pipefishes, seahorses, or seadragons. Arrows show gene order and orientation; gray rectangles represent apparent gene losses. In the leafy seadragon assembly, the region from *spg21* to *ano1* spans approximately from 38.26 to 38.43 Mb on group 2.

The most startling gene losses from the FGF family are shared across the seadragon, tiger tail seahorse, greater pipefish, and bluestripe pipefish lineages: all are missing *fgf3* and *fgf4* (Fig. 6 and *SI Appendix*, Table S1). Blastx searches of Gulf pipefish embryo and brood pouch transcriptome assemblies against seven RefSeq protein sets (*SI Appendix*, *SI Methods*) did not return any hits for *fgf3* or *fgf4*. These searches returned hits for a total of 21,497 unique greater pipefish RefSeq proteins, reflecting deep, diverse coverage of the *Syngnathus* pipefish gene complement and further supporting the loss of *fgf3* and *fgf4*. In percomorph outgroups to the syngnathids, *fgf3*, *fgf4*, and *fgf19* are clustered, with *fgf3* flanked on one side by *ccnd1* and *lto1*, genes that are also missing from seadragons, seahorses, and greater pipefish. But bluestripe pipefish has retained *lto1* (Fig. 6). The expected neighbors normally on the *fgf19* side of the cluster (zgc:153993 and *ano1*) are present in seadragons and both lineages of pipefishes, but bluestripe pipefish has lost *fgf19*. Close outgroups to the syngnathids, the razorfish (*Aeoliscus strigatus*) and the Mandarin dragonet (*S. splendidus*) (63) retain intact *fgf3/4/19* clusters, making this drastic alteration a likely syngnathid synapomorphy (*SI Appendix*, Fig. S12). In the common ancestor of the jawed vertebrates, *fgf3*, *fgf4*, and *fgf19* were likely already clustered (65), and the genes remain tandemly arrayed in available genomes of lobe-finned, ray-finned, and cartilaginous fishes, such as human, coelacanth, zebrafish, and elephant shark (*SI Appendix*, Fig. S12). Of the 80 nonsyngnathid ray-finned fish species whose genome assemblies are currently available in the Ensembl database, only the Lyretail cichlid (*Neolamprologus brichardi*) appears to be missing *fgf3*, likely because of gaps in the assembly. What is more, nearly all of these fish species retain *fgf3/4/19* as a cluster (*SI Appendix*, Table S2). The syngnathid lineage has therefore experienced a degree of change in this cluster that is perhaps unprecedented throughout gnathostome evolution.

Exceptional absences of FGF and FGFR members in extant syngnathid genomes suggest that some property of their syntenic neighborhoods is inherently volatile. We tested whether the local repeat landscape flanking significant regions of FGF, FGFR, and other developmental gene loss in syngnathids—the *fgf3/4/19* cluster, *fgf4l*, *fgf5*, *fgf17*, *fgfr1b*, *eve1* (8), and *tbx4* (8, 10)—differs from that of genes in general, by using the leafy seadragon genome and an outgroup, common dragonet, as a preloss comparator. While no individual repeat class was significantly enriched in these regions (of either genome) after FDR adjustment, several show nominal evidence for enrichment and are at high density in the leafy seadragon regions, including *Tc1*, *BovB*, *hAT-Ac*, and additional LINEs *Rex-Babar*, and *L2* (*SI Appendix*, Fig. S13 and Dataset S4). Furthermore, we found support for the alternative hypothesis that overall repeat density in general is enriched in these regions (*SI Appendix*, Fig. S13 and Dataset S4), only for the leafy seadragon (Pequ: 1.45-fold enrichment; *P* = 0.022), and not the dragonet genome (Clyr: 1.11-fold enrichment; *P* = 0.27).

### Limited Evidence for Compensatory Evolution in the FGF Pathway.

FGF paralogs sometimes overlap in their expression and ability to compensate, partially or fully, for loss of function of one another in specific developmental contexts. In zebrafish, for example, knockdown of either *fgf3* or *fgf10* causes relatively mild effects in lateral line migration and neuromast maturation, but there are severe lateral line defects when expression of both paralogs is depleted (66). We explored whether those paralogs most recently diverged from *fgf3* and *fgf4*, or with overlapping developmental roles, showed evidence for compensatory evolution prior to the syngnathid radiation.

Syngnathid and outgroup sequences are largely conserved for *fgf4-like*, *fgf6a*, *fgf7*, *fgf8a*, *fgf8b*, *fgf10a*, *fgf10b*, *fgf19*, *fgf20a*, *fgf20b*, *fgf22*, and *fgf24*, and exhibit no evidence of positive selection along the syngnathid branch (*SI Appendix*, Table S3). We found potential positive selection in *fgf6b*, although the result was within bounds of false discovery (*SI Appendix*, Fig. S14 and Table S3). Second, although lost in the seadragon and bluestripe pipefish lineages, we found the strongest evidence for positive selection on *fgf17*, which is retained in seahorse and *Syngnathus* pipefish (*SI Appendix*, Table S3).

Like the ligands, the syngnathid FGF receptors are largely conserved, and we found no evidence of syngnathid lineage–specific positive selection for noncanonical *fgfrl1a* and *fgfrl1b*, nor for canonical *fgfr1a*, *fgfr2*, *fgfr3*, or *fgfr4*. In contrast, *fgfr1b* presented evidence for positive selection (although not robust to false discovery), displaying surprising syngnathid-specific substitutions (*SI Appendix*, Table S3). These include a remarkable, likely deleterious substitution within the activation loop of the kinase domain, a position conserved across all canonical FGFR paralogy groups (*SI Appendix*, Fig. S15) (provean score = −6.123).

Although we did not find evidence for lineage-specific positive selection using codon-based models, leafy and weedy seadragons share a derived six amino acid deletion in a conserved region of the Fgf16 protein (*SI Appendix*, Fig. S16), while seahorse has amino acid substitutions in this same motif. Other percomorphs, chicken, and human Fgf16 sequences are identical across this region, though no function has been ascribed to this domain. Divergence of seadragon *fgf16* is not limited to the coding sequence. A putative regulatory change is hinted at by the absence in both dragons of an ∼240-bp conserved CNE that is well preserved across percomorph fishes 5′ of *fgf16* (*SI Appendix*, Fig. S17).

### miRNA-Seq Data from Seadragons Reveal Loss of Conserved microRNAs.

Although microRNAs (miRNAs) are important developmental regulators (67), there were no genome-wide miRNA annotations in syngnathids. In the leafy and weedy seadragons, we identified 259 and 251 miRNA genes that produce 331 and 318 unique mature miRNAs (Dataset S5). The class of conserved miRNA genes lacking both transcript and genomic support included *mir10a* and *mir196b*, previously discovered by a targeted analysis of HOX clusters to be missing in the Gulf pipefish (8). Six of the additional highly conserved missing miRNAs belong to two miRNA clusters from the miR-130 family. These absences include *mir130a*, *mir301a*, *mir130b*, *mir301b*, *mir130c-2*, and *mir454b*. While *mir130a*, *mir301a*, and *mir301b* are convergently missing in platyfish (*X. maculatus*) and medaka (*O. latipes*), absences of *mir130b*, *mir130c-2*, and *mir454b* have not yet been reported in other vertebrates (*SI Appendix*, Fig. S18) (29, 68, 69). In some tetrapods and teleosts, *mir301a* sits in the first intron of *ska2*, which is missing in seadragons. We confirmed that Gulf pipefish, greater pipefishes, and tiger tail seahorse are also missing *ska2*, *mir301a*, and *mir130a.* Throughout vertebrates, *mir130b* and *mir301b* (and, in teleosts and Coelacanth, additionally *mir130c-2* and *mir454b*) lie between *sdf2l1* and *top3b*. Though these adjacent protein coding genes and the immediate syntenic neighborhood are conserved in seadragons, Gulf and greater pipefishes, and tiger tail seahorse, this cluster of microRNAs is not.

## Discussion

Exploring the seadragon genomes in a phylogenetic context has lifted a veil on the evolution of seadragon-specific traits and has also revealed intriguing evolutionary facets of this unusual vertebrate family, the Syngnathidae, as a whole. We found that both leafy and weedy seadragon genomes stand out among their relatives in having a surprisingly large contingent of repetitive DNA. This pattern appears to have been driven largely by recent expansions of *BovB* and *Tc1*, and a yet-to-be-classified family of repetitive sequences specific to the seadragon lineage. One explanation for the large disparity between seadragon and *Syngnathus* pipefish repeatome size and genomic distribution could be a difference in historical effective population size (*N*_e_). With lower *N_e_*, selection to remove rapidly expanding repeats from a population would be less effective owing to stronger genetic drift (70, 71). Differences in k-mer–based heterozygosity estimates from individual WGS data are at least consistent with this idea. A heterozygosity estimate for Gulf pipefish (8) is roughly three times the seadragon estimates (1.01% versus 0.27% and 0.33% for leafy and weedy seadragons).

The above explanation for TE expansion assumes that deleterious effects are common, likely through interruption of coding regions or promoters, or by regional silencing via chromatin changes (72). However, TEs can also contribute new genes or gene regulatory sequences when a host genome coopts (“domesticates”) these exogenous genetic elements (73). Some classes of TE provide, for example, binding sites for master transcriptional regulators NANOG and OCT4 throughout the mouse and human genomes but in largely nonoverlapping sets of loci between the taxa, potentially restructuring, in lineage-specific ways, the transcriptional networks for developmental pluripotency (74). TEs and repetitive DNA in general can fuel gene family expansion or contraction by precipitating unequal crossover events; Hahn et al. (75) suggest an “explosion” of TEs in the primate lineage could be linked to its accelerated gene content evolution. We found evidence for enriched TE density near members of gene families that have undergone significant expansion in the seadragon lineage and a weaker signature of this relationship for seadragon-contracted families and those evolving rapidly in size in nonsyngnathids. Future comparative analyses involving a larger sample of syngnathid genomes will be required to confirm and more precisely quantify this pattern. Repeatome expansion, such as what we here observe in seadragons, could have had disruptive impacts on gene regulatory networks and gene content, subject to subsequent evolution via negative or positive selection.

Using global and targeted approaches, we explored expansion and contraction of gene families in seadragons and their relatives and possible connections of gene content changes to observed TE distributions. Perhaps the most obvious trend we observed for the syngnathid lineage in general was contraction of particular immunity and detoxification pathway gene families, some of which have previously been described (10, 12). GTPase of the immunity-associated protein genes (GIMAP), for example, was among the contracted families in seadragons that was also detected in the seahorse genome (10), and our more comprehensive analysis supports that the GIMAP contraction occurred prior to the radiation of syngnathids. A single eight-member cluster in mammals (76), GIMAP genes have multiplied in other teleost lineages, numbering up to nearly 190 genes. Balla et al. (55) found zebrafish GIMAP genes respond to pathogenic viral exposure and suggest the gene expansion could have been evolutionarily favored by the relatively long period that hatchlings must rely on innate immunity before the development of a functional adaptive immune system. Male-brooded syngnathid embryos might enjoy a luxury not available to free-spawned progeny like those of zebrafish, namely pathogen climate control afforded by the paternal immune system before their own adaptive immunity develops. The male’s immune system strikes a balance of defending against foreign agents but without rejecting his own brood. The syngnathid contraction of the GIMAP family is also interesting, therefore, in light of the possibility that *gimap4*, which does persist in the lineage, could promote immunologic tolerance to embryos in brood pouch tissues (9). Roth et al. (9) based this assertion on the observation that *gimap4* is up-regulated in *Syngnathus* pregnancy tissues, where it could contribute to local suppression of the lymphocyte population (9, 77).

We detected seadragon-specific copy number expansion of a coatamer complex gene *copb2*. Variants of the *BovB* and *Tc1* transposable elements were enriched surrounding the supernumerary gene copies, suggesting a TE-driven mechanism for expansion. Copb2 forms part of a protein complex involved in retrograde vesicle budding from the Golgi apparatus and secretion of macromolecule cargo, such as collagen, which is critical for bone and connective tissue development. Mice and fish developing with deficits in *copb2* have delayed bone mineralization and low bone density, as well as defects in type II collagen trafficking and secretion (78). Zebrafish mutants of *copb2* develop mispatterned, kinked notochords with a disorganized perinotochordal basement membrane, which is secreted by notochord sheath cells (79). In teleosts, the notochord, particularly its sheath, plays an instructive role in patterning the vertebrae (80, 81).

Given the elaborated bony exoskeleton in seadragons, their stiff bodies with connective tissue–dense leafy ornaments, and their kinked axial skeletons with varied and regionalized vertebral forms (Fig. 1), the proliferation of *copb2* sequences ignites curiosity about the possible evolutionary developmental consequences for the seadragons’ unique exo- and endoskeletons. Kinked vertebral columns in the guppy (*Poecelia reticulata*) mutant *curveback* are characterized by wedge-shaped vertebrae (82). Similar to *curveback* and to the developmental malformation of vertebrae in human Scheuermann’s kyphosis sufferers, vertebrae are keystone shaped in the weedy seadragon at locations of spinal curvature (Fig. 1 and *SI Appendix*, Fig. S2).

The most surprising gene family reduction we uncovered is shared by all of the syngnathid lineages we explored; it is the loss of *fgf3* and *fgf4*. Loss of these genes in syngnathids is striking because their orthologs in other vertebrates are thought to play nearly indispensable pleiotropic developmental roles in the pharyngeal arches, teeth, brain, cranial placodes, epidermal appendages, limbs, and the segmental axis (66, 83–97). It is reasonable to weigh whether losing these two multifunctional signaling ligands could have had broad consequences to both deeply conserved developmental pathways and their morphological readouts. Another possibility is that these developmental pathways had diverged neutrally, or through changes in other pathway members, from anciently conserved functions along the syngnathid lineage, permitting genes that had once been critical to become expendable. It is nevertheless valuable to note that syngnathid fishes share peculiarities in many features from the constellation of vertebrate traits that *fgf3* and *fgf4* are known to help pattern.

Dermal integuments of syngnathid fishes are bony plates, and in several syngnathid lineages including the seadragons these have been elaborated to magnificence, sometimes independently. A subset of these plates in seadragons bear blunted struts of bone that end in fleshy paddle-shaped ornaments, the “leaves” and “weeds” (Fig. 1). Another percomorph clade, the pufferfishes, are adorned with bony spines likely evolved from elasmoid scales. Shono et al. (98), showed that *fgf3* is expressed in developing pufferfish dermal spines. Given this and a trove of other evidence for an FGF signaling role in the development and diversification of scales, spines, and denticles in ray-finned and chondrichthyan fishes (96–102), absence of *fgf3* and *fgf4* in the often heavily armored, elaborately spined syngnathids clearly suggests that derived mechanisms for integumentary bone development are at play in this lineage.

Syngnathids have evolved elongated faces with an unusual hyoid apparatus integral to specialized suction feeding (Fig. 1) (103), and they are toothless. Both *fgf3* and *fgf4* are expressed in the pharyngeal arches, which form skeletal elements of the jaw, hyoid, and, in fish, the gill supports (95, 104, 105); *fgf3* plays an essential role in craniofacial development. Additionally, when *fgf3* expression is disrupted in the mesendoderm in developing zebrafish, an “inverted” backward directed ceratohyal cartilage is formed (95). It is tempting to speculate that evolutionary loss of these genes could have led to altered craniofacial architecture of the elongated syngnathid face, either directly or through the effects of genetic compensation percolating through this signaling pathway.

Syngnathid toothlessness is also particularly interesting, given *fgf3* and *fgf4* expression in zebrafish dental epithelium and their suspected roles in tooth morphogenesis (106). Gibert et al. (107) propose a model for patterning of dentition in ray-finned fishes in which a tooth primordium acts as an organizer that induces the development of subsequent teeth, likely via secretion of Fgf3 and Fgf4. Other tooth developmental genes are known to be lost or reduced in copy number in syngnathids, including *eve1* [(8); see above] and P/Q-rich SCPP enamel/enameloid matrix genes (10, 12, 13). These losses imply erosion of tooth development pathways spanning induction to mineralization, with our discovery of *fgf3/4* loss enlarging the pool of candidate causative genes.

The syngnathid central nervous system features its own peculiarities. Benedetti et al. (108) described greater pipefish (*S. acus*) and long-snouted seahorse (*Hippocampus guttulatus*) to have highly modified or no discernable Mauthner neurons, the large, rhombomere 4 (r4) reticulospinal neurons critical for the rapid “C-start” escape response in many fishes and some amphibians (reviewed in ref. 109). Syngnathids are reputed also to lack mechanosensory lateral line neuromasts, from which the Mauthner cells receive synaptic inputs (108, 110). In zebrafish, joint impairment of *fgf3* and *fgf8* impacts segmental identity of rhombomeres 3 and 5 and their reticulospinal neurons (89, 92). Depletion of *fgf3* and *fgf10* reduced the number of zebrafish posterior lateral line neuromasts and inhibited migration of the placode down the length of the body (66). These observations present a compelling case for future interrogation of *fgf3* loss and derived hindbrain and sensory development in syngnathids.

Though it is known that paralogous FGF ligands can compensate for one another in some experimental contexts (89, 92), in general, we did not find sweeping evidence for adaptive evolution of paralogous FGF proteins or their receptors in the wake of syngnathid *fgf3* and *fgf4* gene losses. Correlated changes could instead have included evolution of noncoding, regulatory sequences of FGF/FGFR genes or changes to other gene families that interact with FGF signaling. Syngnathids, we found, have lost six deeply conserved miRNAs in the miR-130 family. Biological implications for these missing miRNA genes are uncertain, though it is possible that their losses could relate to derived syngnathid-specific traits and gene pathway changes. For instance, angiogenesis and tissue remodeling are critical components in syngnathid male pregnancy tissues (111), and mir130a is connected to vascular repatterning in mammals and teleosts (112, 113). mir130 genes are also known to interact with FGF signaling (114, 115, 116, 117). Loss of miRNAs that regulate and are potentially regulated by FGFs and FGFRs could indicate a further restructuring of FGF signaling pathways in syngnathids.

Evidence for positive selection as a compensatory consequence of having lost *fgf3* and *fgf4* in a syngnathid ancestor proved to be scarce. Derived functional change of *fgf16* in the seadragon lineage, at both the protein and cis-regulatory levels, provides a possible example of a separate evolutionary scenario. Leafy and weedy seadragon fgf16 proteins share an unusual deletion in a deeply conserved motif, and the seadragon gene also appears to have lost a 5′ noncoding sequence that is otherwise conserved among syngnathids and distantly related percomorphs. In zebrafish, this gene is necessary for outgrowth of the pectoral fin, upstream of *fgf4* and *fgf8* (118), and we show it is expressed similarly in fin margins in a representative percomorph (*SI Appendix*, Fig. S19). The eponymous leaves of seadragons that are fleshy extensions borne on bony supports are apparently stiffened by a core of collagenous tissue rather than ossified structures such as fin rays (Fig. 1). Homology of the leafy ornaments with fins might pertain only at the level of shared genetic pathways, perhaps via redeployment of FGF signaling for outgrowth of these superficially fin-like structures. A role in scale patterning for *fgf16* is not known from teleosts; though in birds its ortholog can suppress both *shh* expression and downy feather elongation (119). The seadragon-specific changes in a known AER and integument-patterning gene are intriguing in the context of the leafy “appendages.” The seahorse fgf16 protein bears substitutions in the same amino acid motif deleted in seadragons. The fact that two lineages that have evolved elaborate bony and fleshy ornaments show divergence in a conserved motif of this gene is tantalizing and warrants further comparative work.

## Supplementary Material

Supplementary File

Supplementary File

Supplementary File

Supplementary File

Supplementary File

Supplementary File

## Data Availability

DNA sequence data have been deposited in National Center for Biotechnology Information databases under BioProjects PRJNA765699 and PRJNA765702 (121, 122). Summary files and R code have been deposited in Dryad (https://doi.org/10.5061/dryad.31zcrjdmf) (123). The X-ray scans are available via the MorphoSource repository (https://www.morphosource.org/concern/biological_specimens/000436213) (124). All other study data are included in the article and/or supporting information.
