# Supplementary material for: Leafy and weedy seadragon genomes connect genic and repetitive DNA features to the extravagant biology of syngnathid fishes
Source: Proc Natl Acad Sci U S A. 2022 Jun 22;119(26):e2119602119. doi: 10.1073/pnas.2119602119 (PMC9245644; doi:10.1073/pnas.2119602119)
Supplement: Supplementary File [file pnas.2119602119.sapp.pdf]

Supplemental Figures, Tables, and Dataset descriptions

LEAFY AND WEEDY SEADRAGON GENOMES CONNECT GENIC AND REPETITIVE DNA FEATURES TO THE EXTRAVAGANT BIOLOGY OF SYNGNATHID FISHES

Clayton M. Small, Hope M. Healey, Mark C. Currey, Emily A. Beck, Julian Catchen, Angela S. P. Lin, William A. Cresko, and Susan Bassham

SUPPLEMENTAL FIGURES:

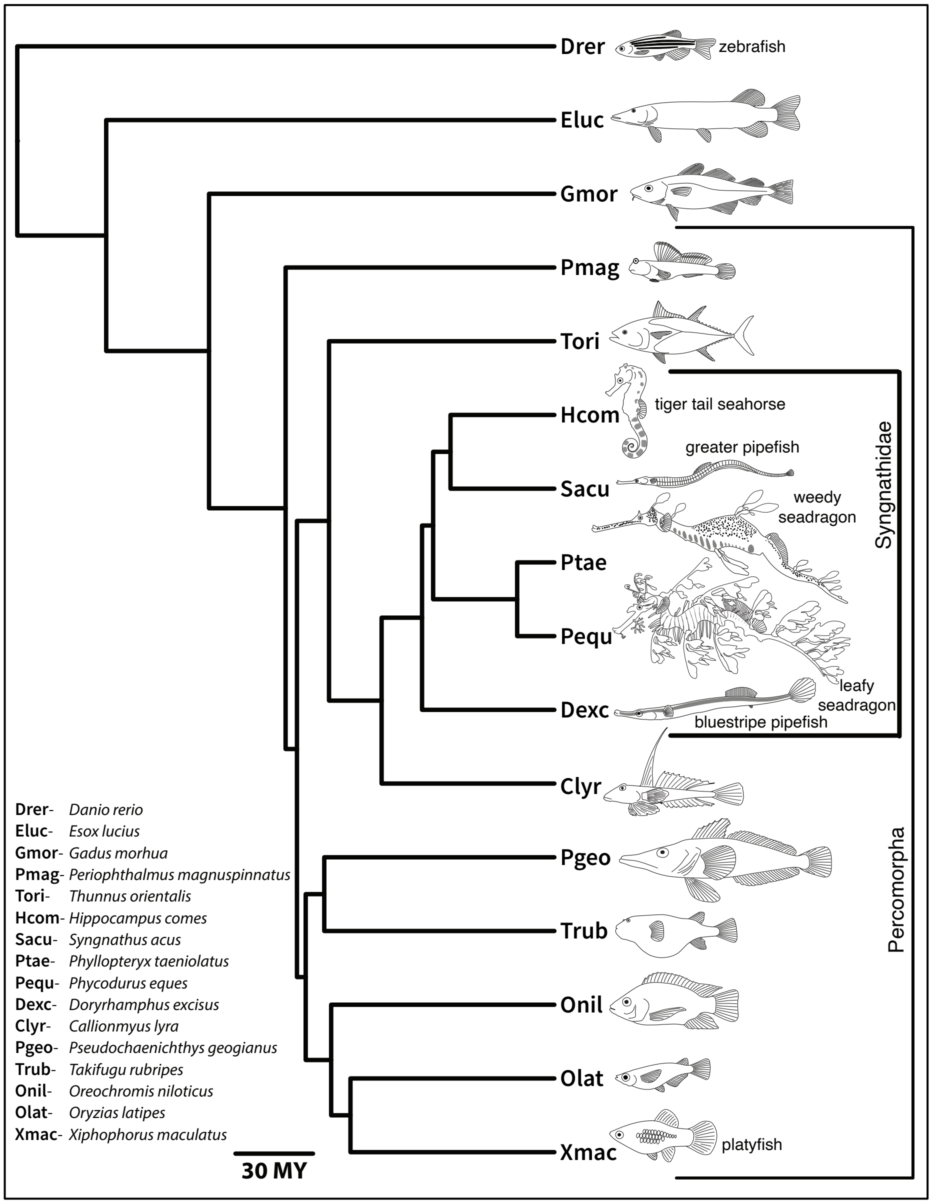

Figure S1. Genomes of 16 teleost species provide phylogenetic context for the contributions of repetitive DNA to genome evolution. Represented are evolutionary relationships among a sample of diverse teleosts, according to a time-calibrated phylogenetic tree adapted from Rabosky et al (1, 2). A major aim with the selection of these species was to choose genomes assembled with long-read (or at

the very least linked-read) technology to the extent possible, to minimize exclusion of repetitive regions from our analysis.

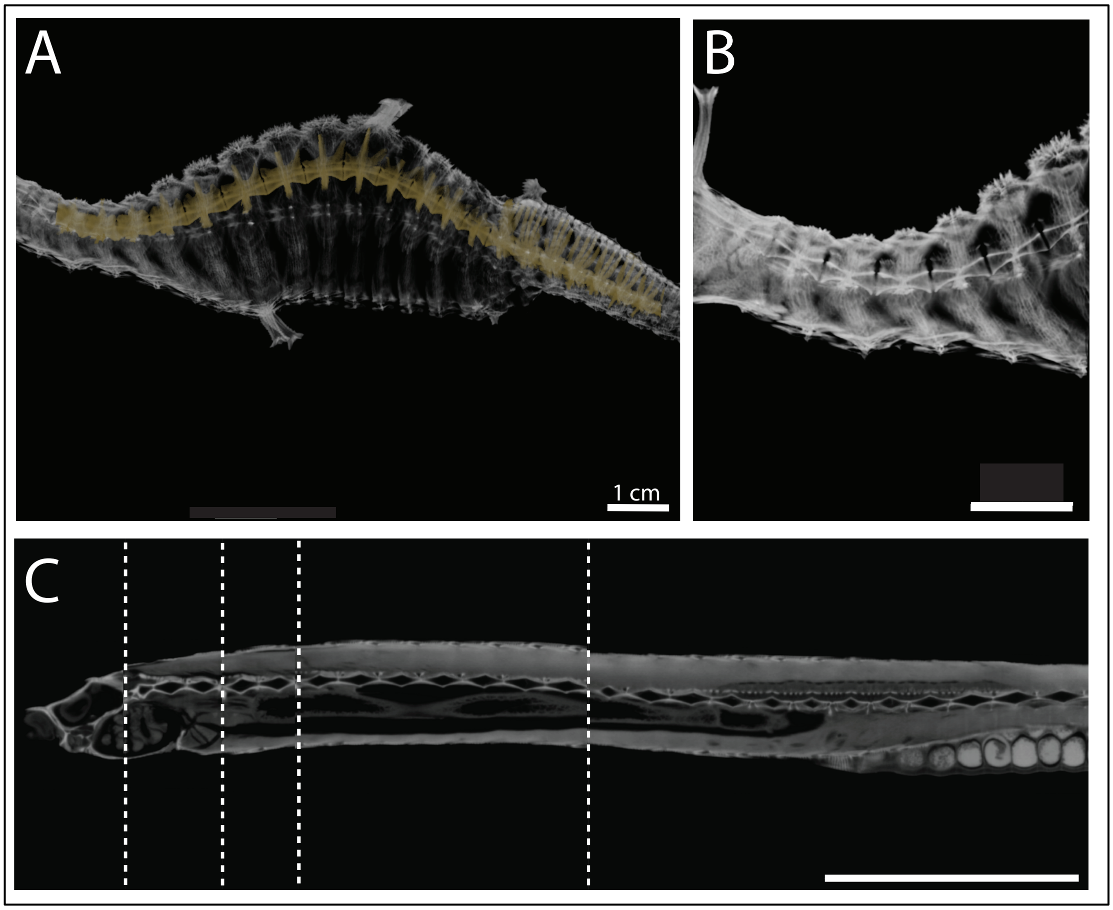

Figure S2. Vertebral shape contributes to spinal curvature in weedy seadragon. A) a lateral view of the seadragon with the vertebrae highlighted. B) a lateral detail of the keystone shaped vertebrae close to the head. C) The vertebrae of the straight-bodied Gulf pipefish (Syngnath*us scovelli*) are relatively homogenous in shape. Because of slight lateral curvature of the scanned specimen, a pregnant male, we show here a composite image, with vertical dashed lines marking junctions between the slightly different optical sections needed to follow a midline sagittal plane along the fish. Scale bars = 1 cm.

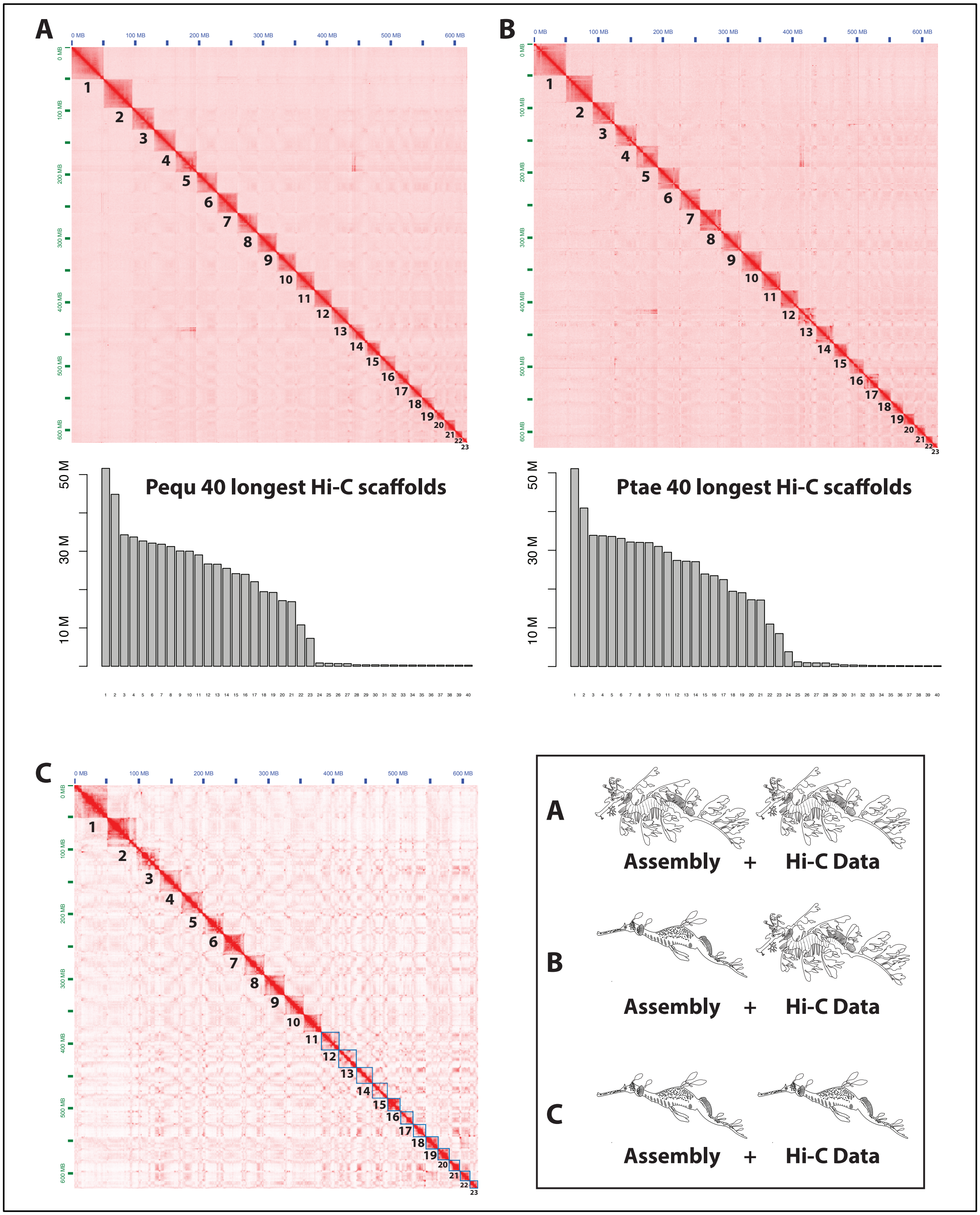

Figure S3. Hi-C sequencing of proximity-ligated DNA fragments facilitates the organization of PacBio contigs into seadragon chromosome models. Shown are contact maps, produced by Juicebox (3) reflecting the frequency of DNA proximity events across the 23 putative chromosomes (numbered) after Hi-C scaffolding, and the length distributions for the 40 longest Hi-C scaffolds. Final versions of the leafy

and weedy seadragon assemblies (A and B) were each scaffolded using Hi-C data generated from the leafy seadragon. Hi-C data were sequenced for the weedy seadragon, but scaffolding that assembly using the matched species data was problematic (C), possibly due to inferior library preparation.

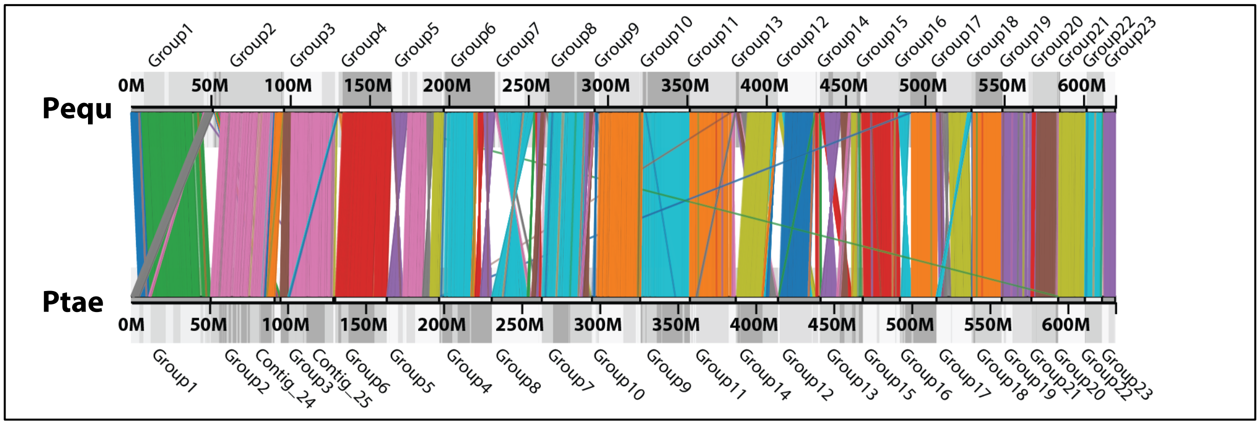

Figure S4. The assembled genomes of leafy (Pequ) and weedy (Ptae) seadragons have highly conserved gene synteny on a fine scale. Strings, colored by synteny cluster, connect orthologous genes and groups of genes. A "cluster" is a block of orthologous genes that occur in the same syntenic neighborhood in both genomes, so large blocks of a single color show regions of highly parallel contiguity of orthologs. Hi-C chromosomes (and in two Ptae cases, large Hi-C scaffolds unassigned to chromosomes) are colored in alternating light and dark grey bars, and contig boundaries are indicated by alternating light and dark grey shaded boxes.

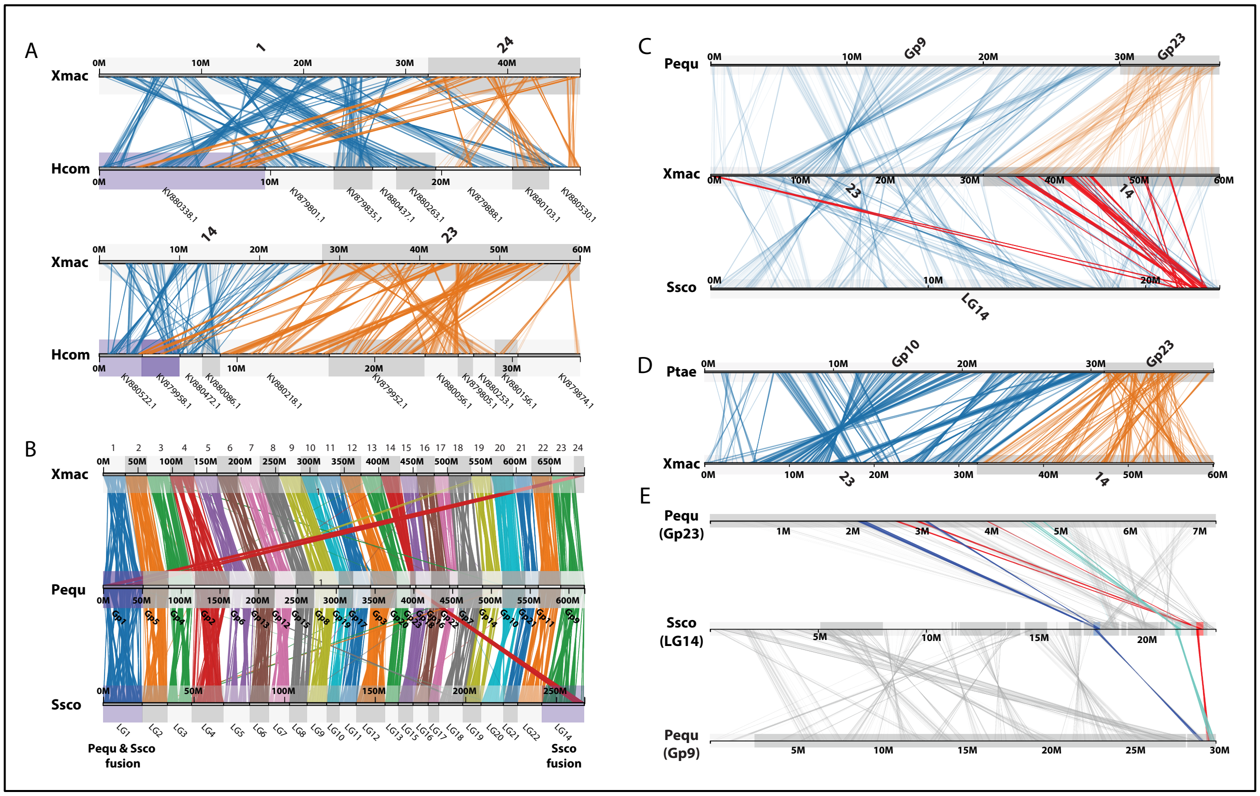

Figure S5. The evolutionary sequence of chromosomal fusion events in syngnathid lineages is illustrated by conserved gene synteny comparisons. Fusion of ancestral chromosomes 1 and 24 (numbered as in the non-syngnathid comparator species, platyfish) occurred in a common ancestor of seadragons, seahorses, and *Syngnathus* pipefish, but the seahorse+pipefish fusion of chromosomes 14 and 23 (also numbered as in platyfish) occurred after the seadragon lineage diverged. Blocks of conserved gene orthologs are connected by colored strings rooted at their physical positions along the length of the genome assemblies for compared species. A) Of the tiger tail seahorse scaffolds (alternating light and dark shaded bars) that are orthologous to platyfish chromomsomes 1, 2, 14, and 23, some scaffolds (shaded in lavender) appear to capture the junctions of ancestral chromosomes that became fused in the lineage leading to both seahorses and *Syngnathus* pipefish. Strings are colored by platyfish chromosome. B) Leafy seadragon and Gulf pipefish share the fusion of ancestral chromosomes 1 and 24, but the seadragon orthologs of chromosomes 14 and 23 remain separate. Pipefish fusion chromosomes are shaded in lavender. C) Gulf pipefish has rearrangements (emphasized by red strings) in the fusion chromosome relative to the non-syngnathid outgroup species, platyfish, that blend gene synteny blocks across the ancestral border between platyfish Chromosome 14 and 23. Similar rearrangements in seahorse can be seen in panel A. In neither species of seadragon do we detect such regions of syntenic gene orthologs that connect a single seadragon group to both platyfish chromosomes (C and D). E) Strings colored by scaffold illustrate the pipefish rearrangement relative to seadragon, where individual pipefish scaffolds have orthology to both leafy seadragon groups (Pequ 9 and 23). Hcom, tiger tail seahorse; Pequ, leafy seadragon; Ptae, weedy seadragon; Ssco, Gulf pipefish; Xmac, platyfish.

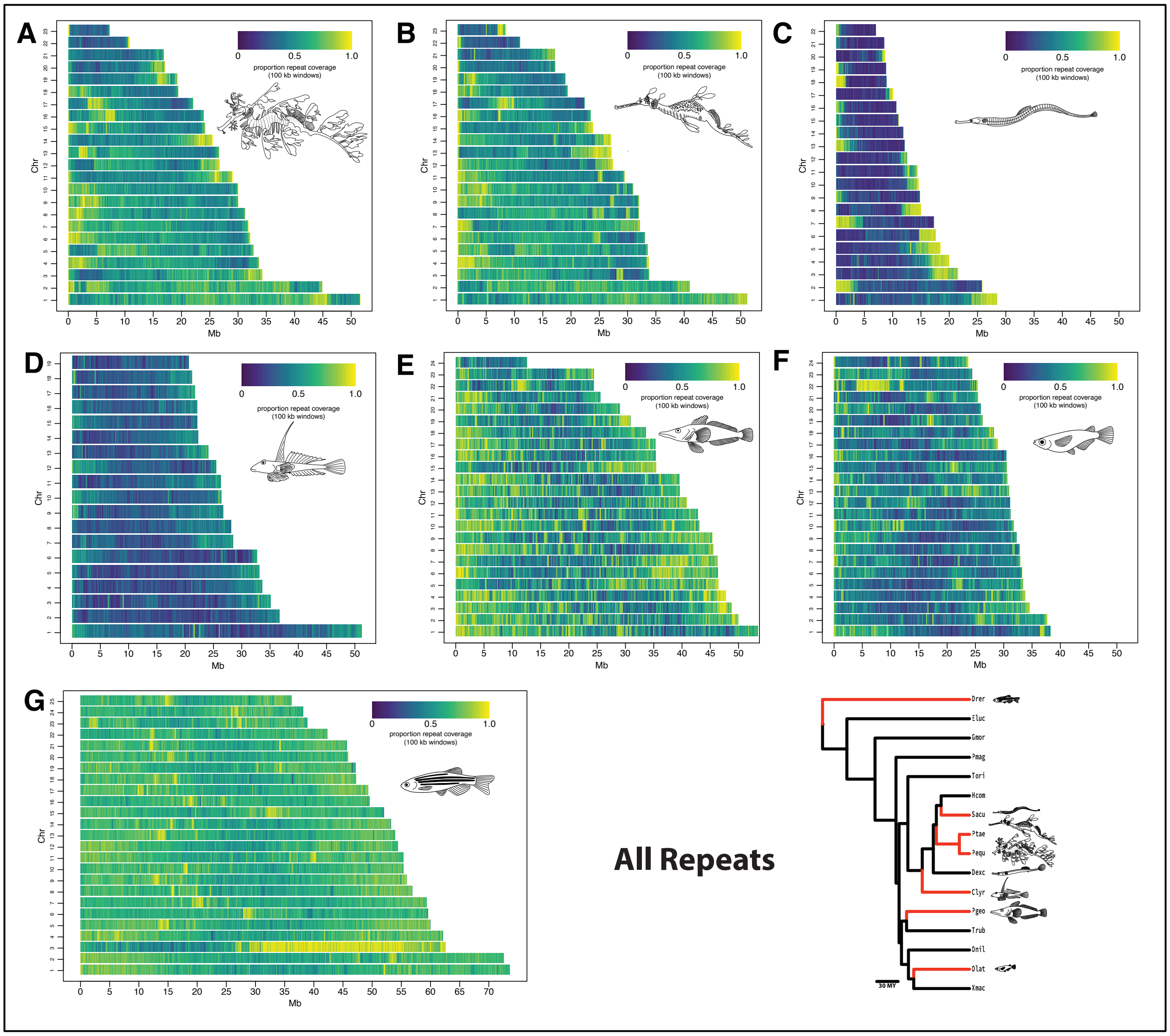

Figure S6. Chromosomal variation in repeat density among seven chromosome-scale teleost genome assemblies. Overall repeat basepair occupation of 100 kb windows (expressed as a proportion) is presented as a heatmap for A) leafy seadragon, *Phycodurus eques*; B) weedy seadragon, *Phyllopteryx taeniolatus*; C) greater pipefish, *Syngnathus acus*; D) common dragonet, *Callionymus lyra*; E) South Georiga icefish, *Pseudochaenichthys georgianus*; F) Japanese medaka, *Oryzias latipes*; and G) zebrafish, *Danio rerio*.

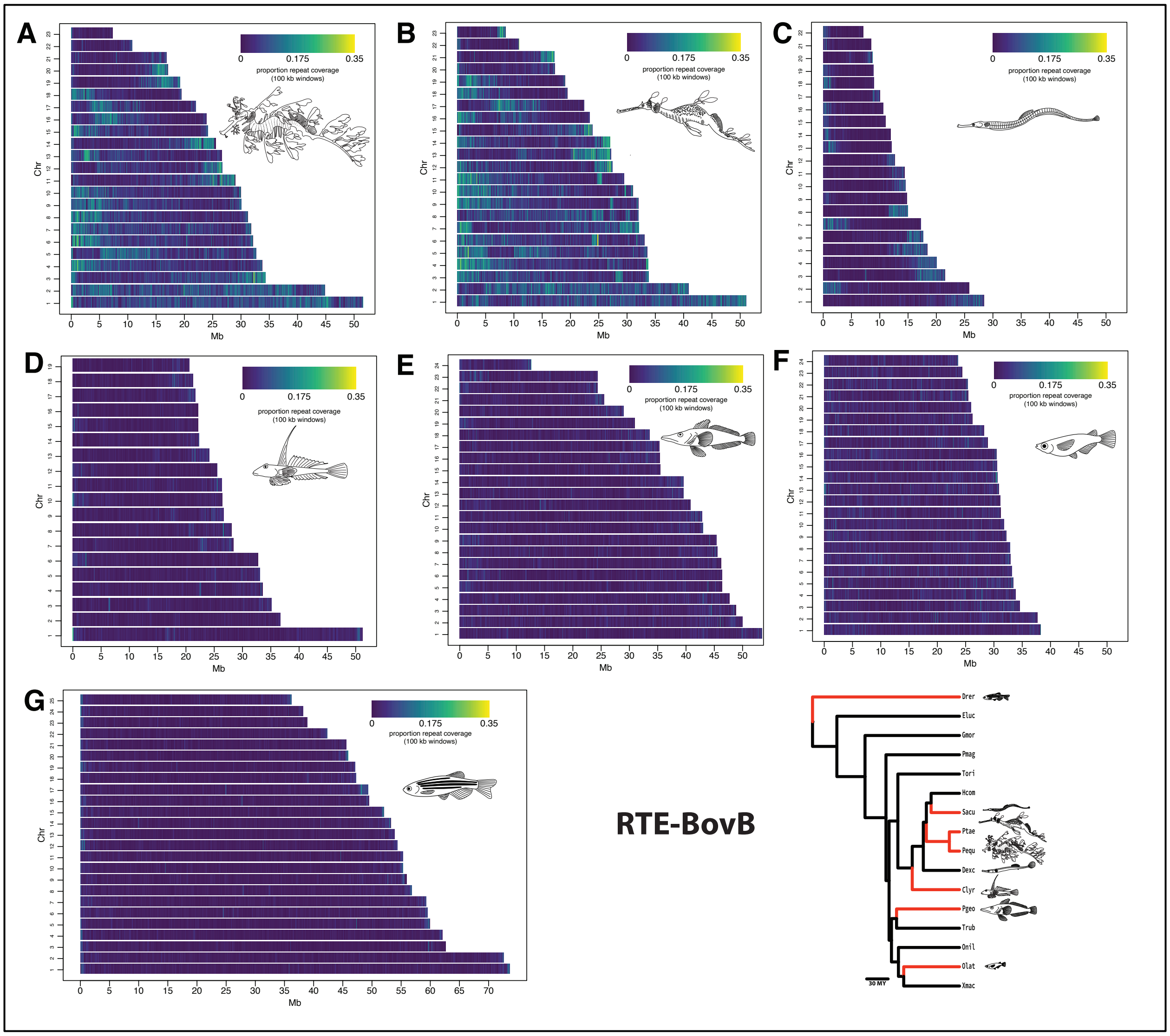

Figure S7. Chromosomal variation in the density of RTE-BovB (a LINE retrotransposon) repeats among seven chromosome-scale teleost genome assemblies. BovB basepair occupation of 100 kb windows (expressed as a proportion) is presented as a heatmap for A) leafy seadragon, *Phycodurus eques*; B) weedy seadragon, *Phyllopteryx taeniolatus*; C) greater pipefish, *Syngnathus acus*; D) common dragonet, *Callionymus lyra*; E) South Georgia icefish, *Pseudochaenichthys georgianus*; F) Japanese medaka, *Oryzias latipes*; and G) zebrafish, *Danio rerio*. BovB repeats are particularly abundant in seadragon genomes.

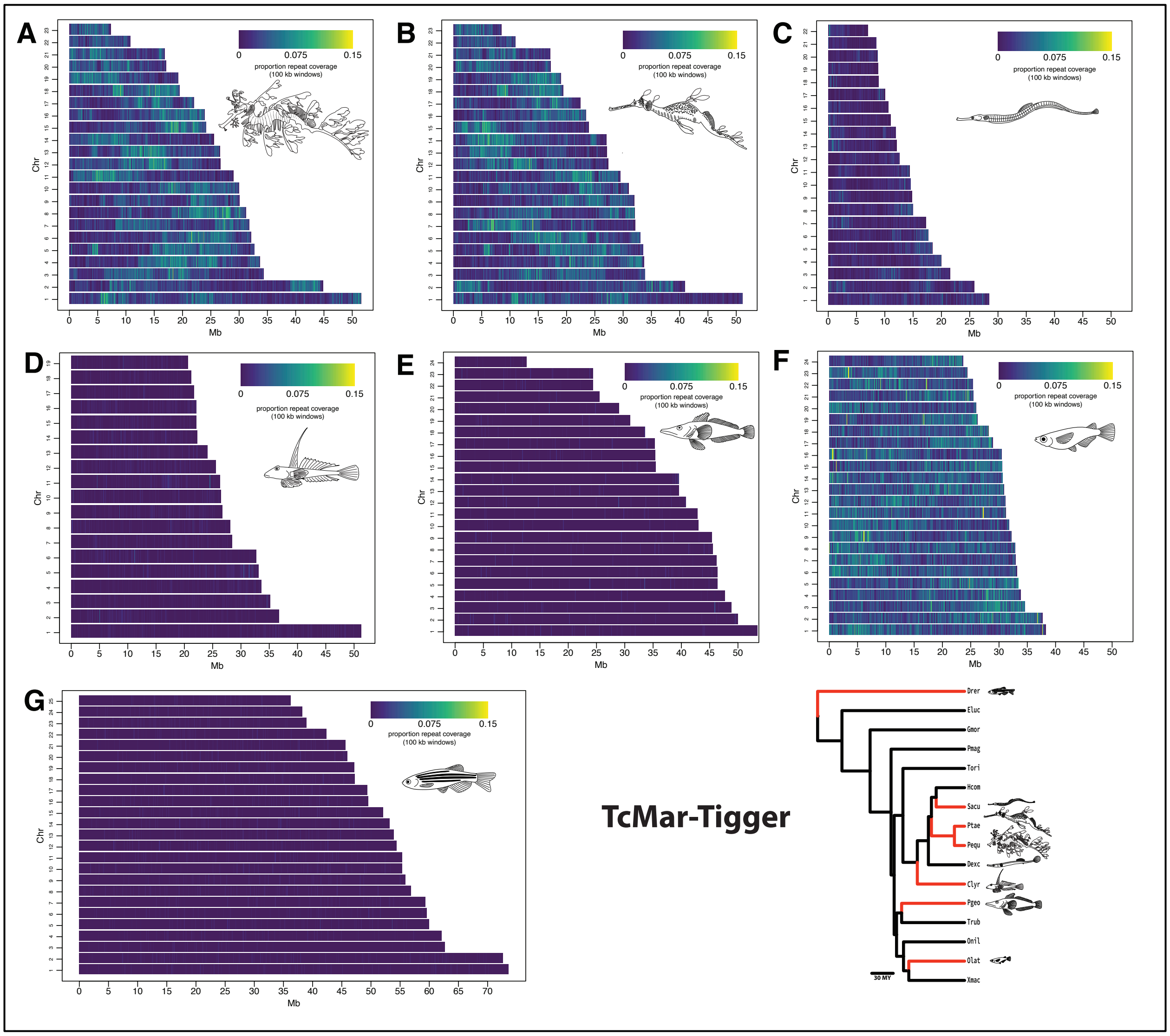

Figure S8. Chromosomal variation in the density of TcMar-Tigger (a DNA transposon) repeats among seven chromosome-scale teleost genome assemblies. Tigger basepair occupation of 100 kb windows (expressed as a proportion) is presented as a heatmap for A) leafy seadragon, *Phycodurus eques*; B) weedy seadragon, *Phyllopteryx taeniolatus*; C) greater pipefish, *Syngnathus acus*; D) common dragonet, *Callionymus lyra*; E) South Georiga icefish, *Pseudochaenichthys georgianus*; F) Japanese medaka, *Oryzias latipes*; and G) zebrafish, *Danio rerio*. Tigger repeats are particularly abundant in syngnathid genomes, as well as in other genomes such as medaka. Also note the "patchy" distribution of Tigger repeats in seadragon and greater pipefish genomes, in regions that are largely incongruent with generally high repeat density in those genomes.

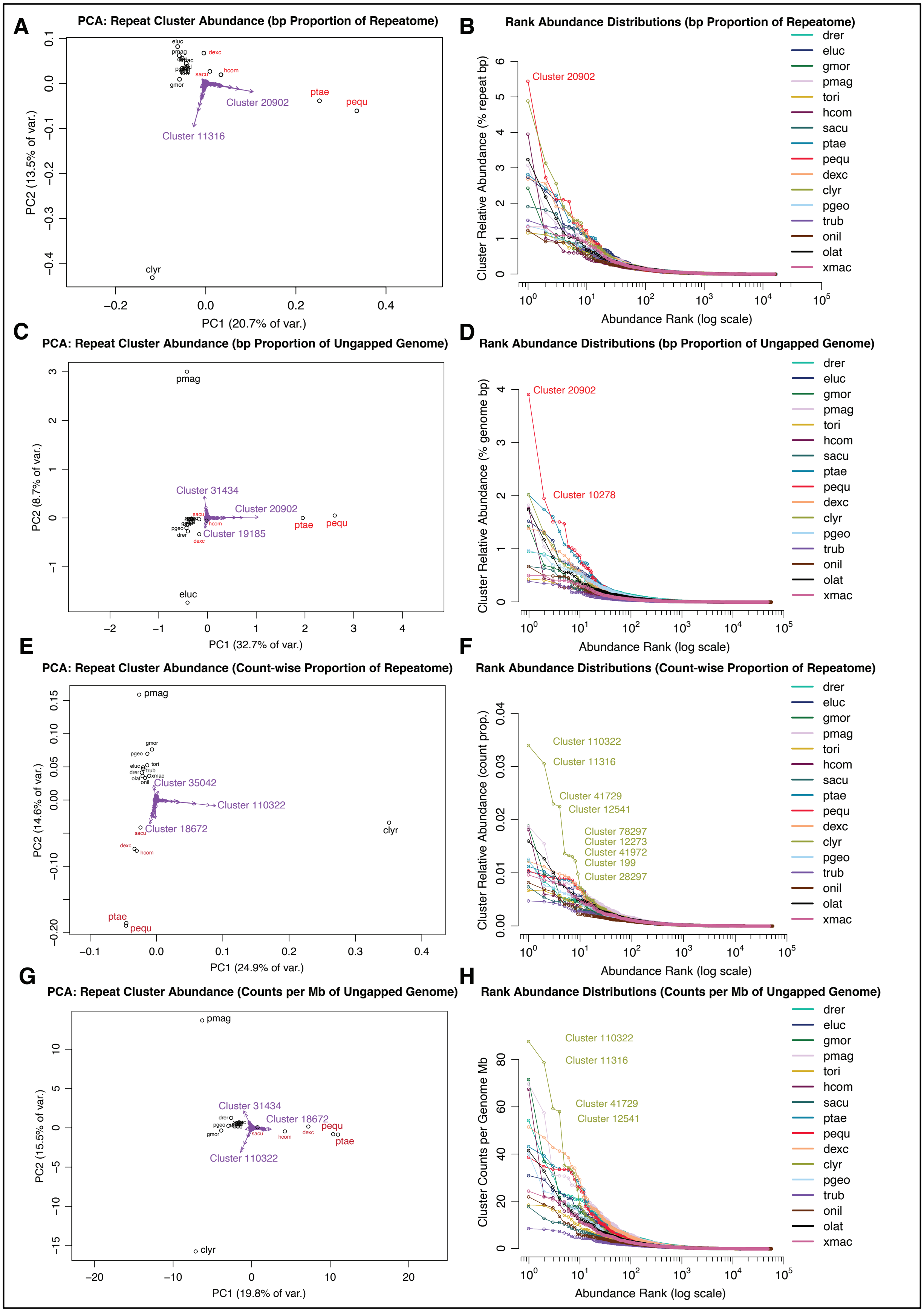
A
PCA: Repeat Cluster Abundance (bp Proportion of Repeatome)
PC1 (20.7% of var.)
PC2 (13.5% of var.)
Cluster 20902
Cluster 11316
ptae
pequ
clyr
B
Rank Abundance Distributions (bp Proportion of Repeatome)
Cluster Relative Abundance (% repeat bp)
Abundance Rank (log scale)
Cluster 20902
drer
eluc
gmor
pmag
tori
hcom
sacu
ptae
pequ
dexc
clyr
pgeo
trub
onil
olat
xmac
C
PCA: Repeat Cluster Abundance (bp Proportion of Ungapped Genome)
PC1 (32.7% of var.)
PC2 (8.7% of var.)
pmag
Cluster 31434
Cluster 20902
Cluster 19185
ptae
pequ
eluc
D
Rank Abundance Distributions (bp Proportion of Ungapped Genome)
Cluster Relative Abundance (% genome bp)
Abundance Rank (log scale)
Cluster 20902
Cluster 10278
E
PCA: Repeat Cluster Abundance (Count-wise Proportion of Repeatome)
PC1 (24.9% of var.)
PC2 (14.6% of var.)
pmag
Cluster 35042
Cluster 110322
Cluster 18672
clyr
ptae
pequ
F
Rank Abundance Distributions (Count-wise Proportion of Repeatome)
Cluster Relative Abundance (count prop.)
Abundance Rank (log scale)
Cluster 110322
Cluster 11316
Cluster 41729
Cluster 12541
Cluster 78297
Cluster 12273
Cluster 41972
Cluster 199
Cluster 28297
G
PCA: Repeat Cluster Abundance (Counts per Mb of Ungapped Genome)
PC1 (19.8% of var.)
PC2 (15.5% of var.)
pmag
Cluster 31434
Cluster 18672
Cluster 110322
pequ
ptae
clyr
H
Rank Abundance Distributions (Counts per Mb of Ungapped Genome)
Cluster Counts per Genome Mb
Abundance Rank (log scale)
Cluster 110322
Cluster 11316
Cluster 41729
Cluster 12541

Figure S9. Alternative methods for quantifying repeat cluster abundance consistently reveal uniqueness of seadragon repeatomes. Shown are principal components analysis (PCA) bi-plots in the left column of panels, and rank abundance distributions (RADs) in the right column. Repeat clusters were quantified as (A-B) the relative proportion of total repeat basepairs, (C-D) the relative proportion of the entire genome basepairs, (E-F) the count-wise proportion of total repeat counts, and (G-H) as repeat counts per Mb of genome. In each plot pair, individual repeat clusters that load heavily on PCs or are especially abundant in individual genomes are noted in purple (PCA plots) or the matching species color (RAD plots).

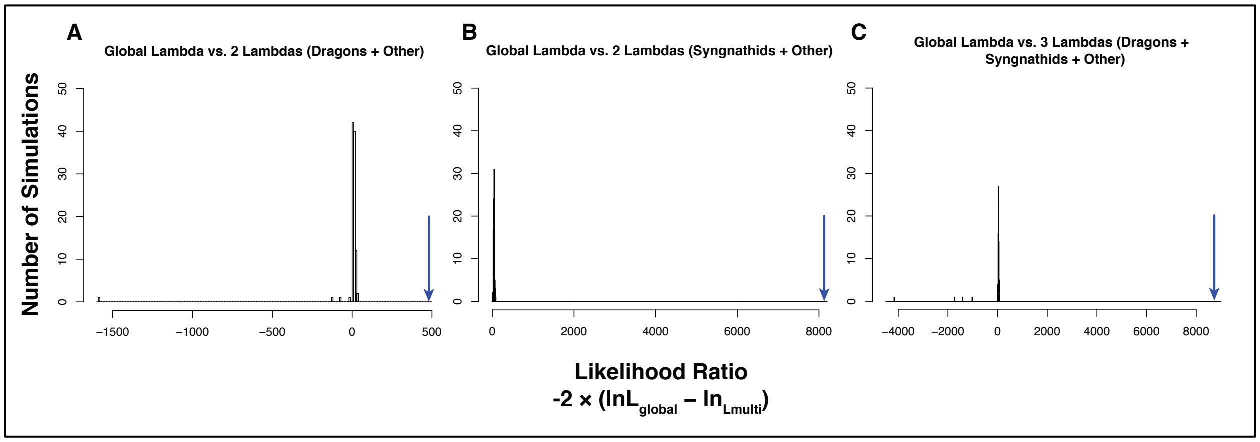

Figure S10. Evolutionary simulations with CAFE 5 (4) provide evidence for seadragon and syngnathid branch-specific gene family expansion/contraction rates (λ) that are distinct from a global estimate for the 21-species tree. We compared the likelihood for a single-λ model to that of a multi-λ model in which branches of interest had a separate, distinct λ, using the likelihood ratio (-2 × ($\ln L_{global} - \ln L_{multi}$)). For each test we compared the likelihood ratio observed from the data to a distribution based on 100 Brownian Motion gene family evolution simulations. Specific branch tests included (A) a distinct λ for the internal seadragon ancestor branch, (B) a distinct λ for the internal syngnathid ancestor branch, and (C) distinct λs for both the seadragon and syngnathid ancestor branches. Shown are histograms (gray bars) of the simulated likelihood ratios, and blue arrows marking the LRs observed from the actual data.

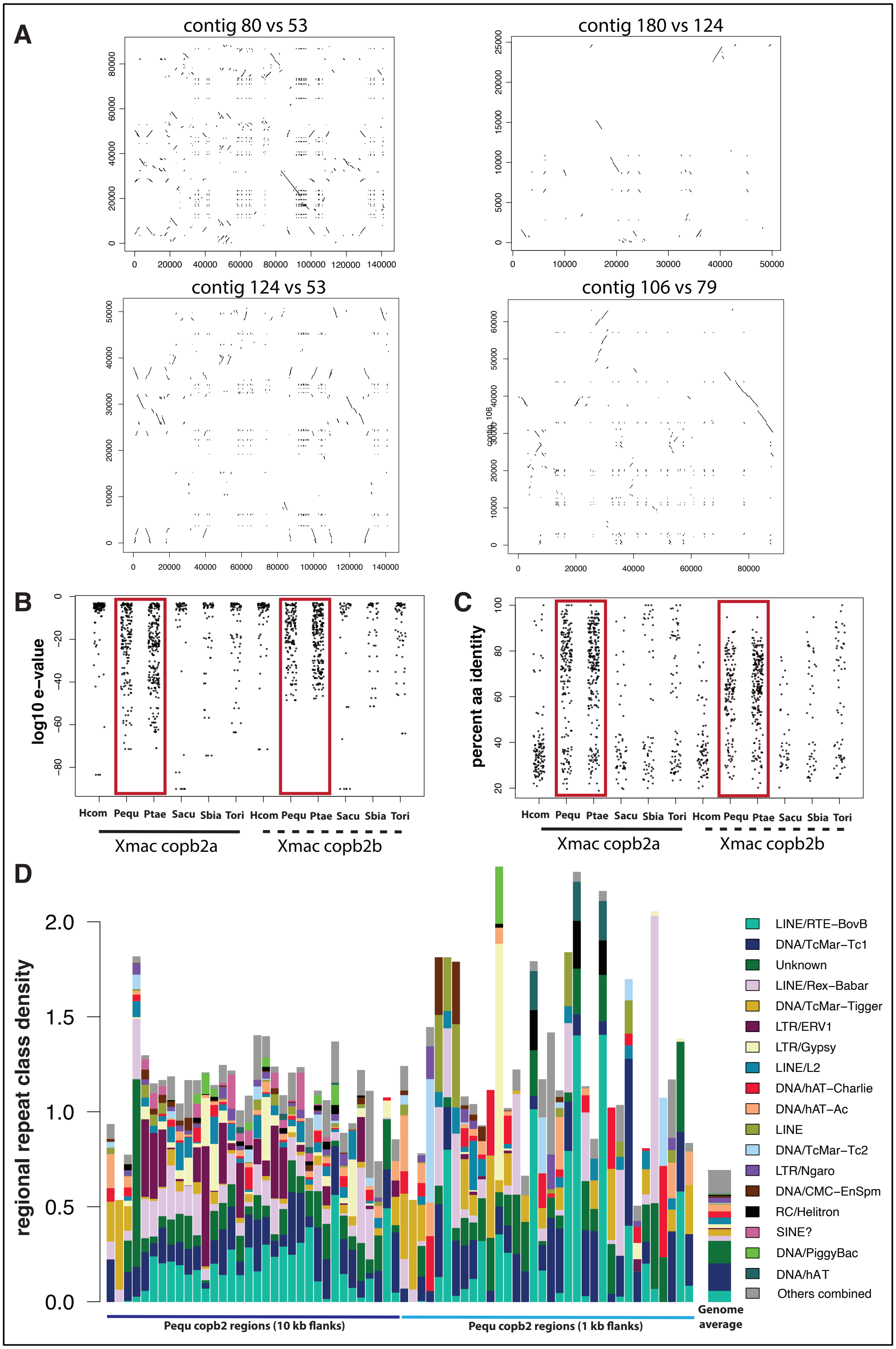

A
contig 80 vs 53
contig 180 vs 124
contig 124 vs 53
contig 106 vs 79
B
log10 e−value
Hcom Pequ Ptae Sacu Sbia Tori Hcom Pequ Ptae Sacu Sbia Tori
Xmac copb2a
Xmac copb2b
C
percent aa identity
Hcom Pequ Ptae Sacu Sbia Tori Hcom Pequ Ptae Sacu Sbia Tori
Xmac copb2a
Xmac copb2b
D
regional repeat class density
LINE/RTE−BovB
DNA/TcMar−Tc1
Unknown
LINE/Rex−Babar
DNA/TcMar−Tigger
LTR/ERV1
LTR/Gypsy
LINE/L2
DNA/hAT−Charlie
DNA/hAT−Ac
LINE
DNA/TcMar−Tc2
LTR/Ngaro
DNA/CMC−EnSpm
RC/Helitron
SINE?
DNA/PiggyBac
DNA/hAT
Others combined
Pequ copb2 regions (10 kb flanks)
Pequ copb2 regions (1 kb flanks)
Genome average

Figure S11. Leafy and weedy seadragon genome assemblies provide evidence for expansions of *coatomer subunit beta 2* (*copb2*) genes. A) Dotplot examples reflecting genomic sequence alignments of regions from the weedy seadragon genome containing putative *copb2* copies. The dotplots do not show large tracks of conserved sequence between regions, suggesting that they are not alternative haplotypes assembled separately. B) Distributions of log-transformed e-values for tblastn queries of platyfish (Xmac) *copb2* paralogs against tiger tail seahorse (Hcom), leafy and weedy seadragons (Pequ and Ptae), greater pipefish (Sacu), alligator pipefish (Sbia) and Pacific bluefin tuna (Tori) genomes. C) Distributions of amino acid percent identity for tblastn queries of *X. maculatus* (platyfish) *copb2* paralogs against the genomes listed in B. Each point in B and C represents an individual blast high-scoring pair (hsp), so multiple points per gene are expected. Tblastn hits in the two seadragon genomes are boxed in red, illustrating many more high-scoring *copb2* hits relative to the other genomes. Because Sbia shares a recent common ancestor with seadragons, the expansion is likely specific to the seadragon lineage. D) Repeat landscapes in *copb2* regions and flanking sequence within the leafy seadragon genome. Each region reflects an individual gene (or pseudogene), and either 10 kb flanking sequence in both directions (first 34 bars) or 1 kb flanking sequence in both directions (second 34 bars). Colors in stacked bars reflect summed basepairs annotated for a specific repeat class divided by the total length of the region. The last, thick bar reflects mean repeat class abundances from 499 random samples of 34 genes (and their 10 kb flanking regions), used as a genomic "background" for comparison. The background repeat frequencies for 1 kb flanking regions were nearly identical, and are not shown. Bars can exceed y-axis values of 1.0 due to overlapping repeat annotations (e.g., those on opposite strands).

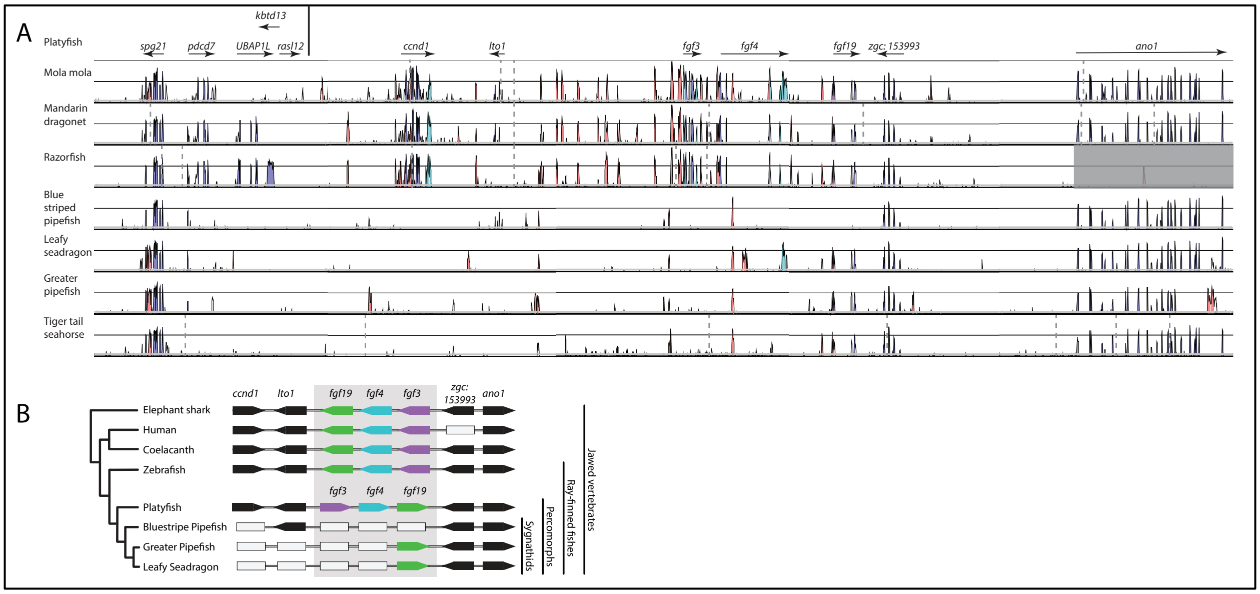

Figure S12. Syngnathids have uniquely lost *fgf3, fgf4,* and other neighboring genes. A) These losses are demonstrated on the VISTA plot with platyfish as the reference. In the plot, conserved exons are marked in dark blue peaks, conserved non-coding sequences in pink, and conserved three prime untranslated regions in light blue. Gaps in the tiger tail seahorse, mola mola, mandarin dragonet, and razorfish assemblies are noted with grey dashed lines (small gaps) and grey shading (a larger gap of unknown absolute size). There is a 150 kb gene sparse region that is removed from the VISTA and noted with the black bar. Although *ano1* appears missing in razorfish, it is present on a different contig than *fgf3* and

fgf4 due to a fragmented assembly. B) The unusual losses in syngnathids are contrasted against a backdrop of deep conservation of the syntenic neighborhood across other jawed vertebrates, despite the apparently derived inversion of the *fgf3/4/19* cluster in the percomorph lineage.

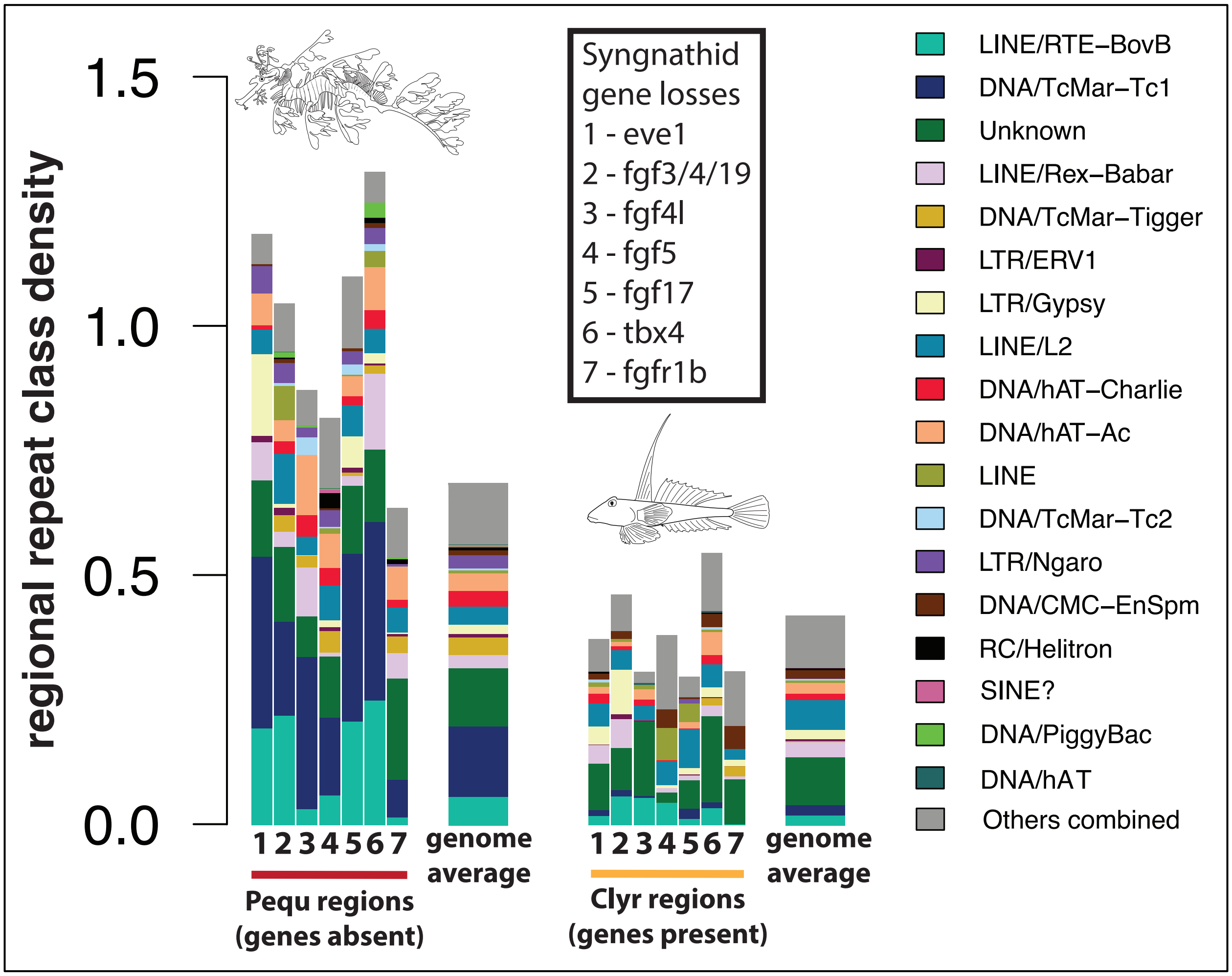

Figure S13. Repeat density is elevated in key regions of syngnathid-specific developmental gene loss. Repeat landscapes at seven regions in which highly conserved developmental genes have been lost in syngnathid fishes are shown using repeat density bar plots for leafy seadragon (as a syngnathid representative), and orthologous regions in a close outgroup, the common dragonet. Colors in stacked bars reflect summed basepairs annotated for a specific repeat class divided by the total length of the region. Thick bars reflect mean repeat class abundances from 499 random samples of seven genes (with regions extending to immediate flanking genes in both directions), used as genomic "backgrounds" for comparison. Bars can exceed y-axis values of 1.0 due to overlapping repeat annotations (e.g., those on opposite strands). Overall repeat density is significantly higher in the focal regions relative to the background distribution in the seadragon, but not the dragonet, genome.

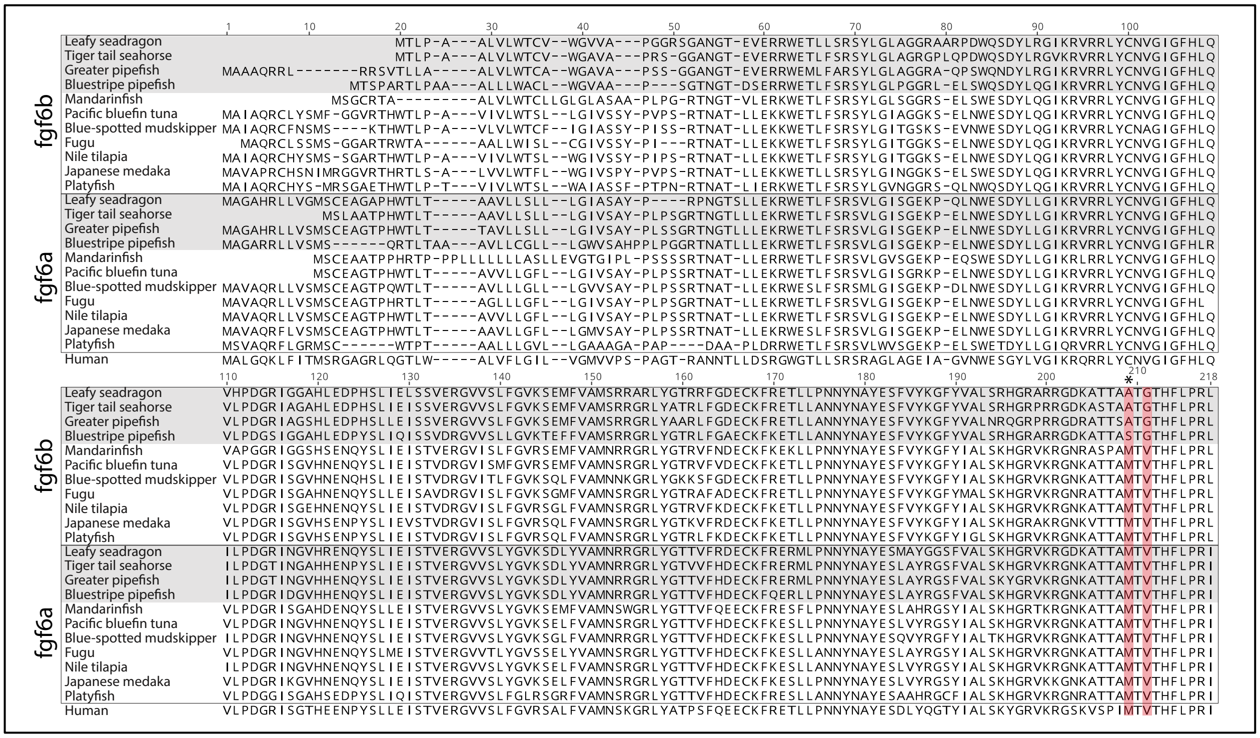

Figure S14. *fgf6b* was inferred to have experienced positive selection (before adjusting for false discovery rate) in syngnathids, and one of the derived substitutions in this category is predicted to be deleterious. Aligned Fgf6b protein sequences from percomorph fishes and human are shown, with syngnathid sequences highlighted in grey. Two syngnathid-specific mutations at deeply conserved sites are highlighted in red; the site with the asterisk was predicted to be deleterious by Provean (5). By contrast syngnathid Fgf6a has maintained these conserved amino acids.

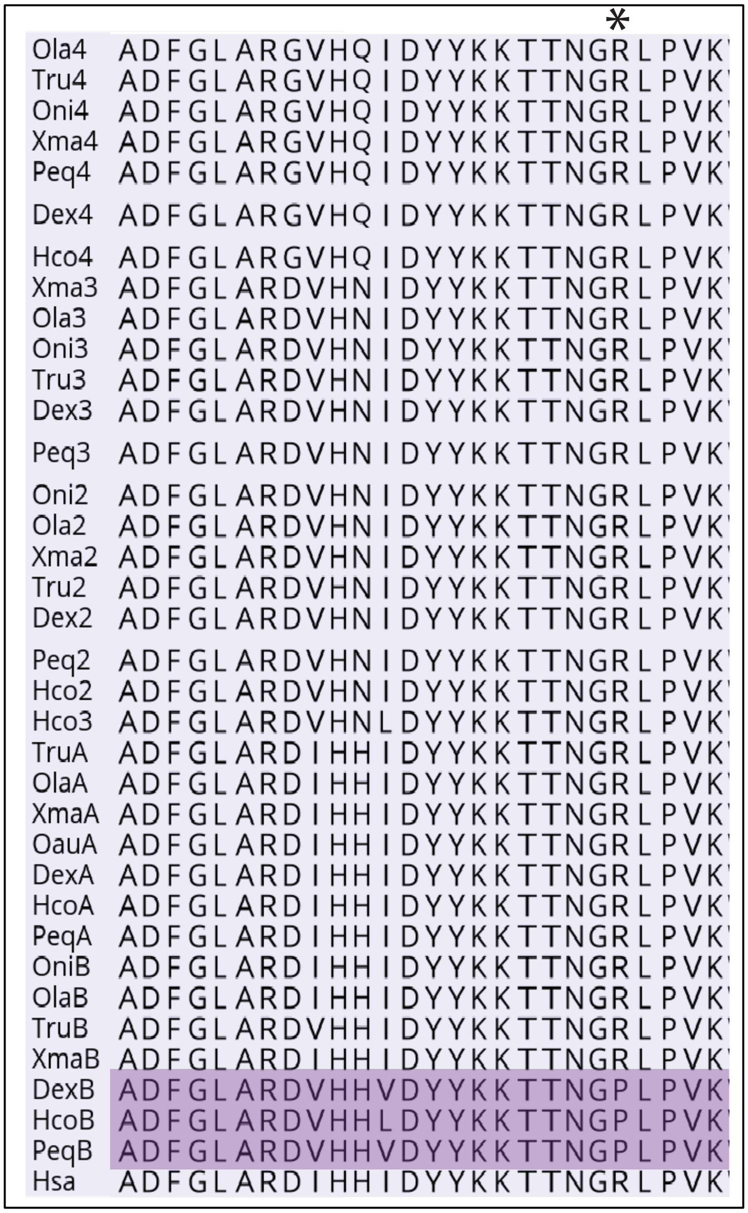

Figure S15. A unique substitution in the deeply conserved activation loop of syngnathid *fgfr1b*. The activation loop from teleost fish *fgfr* sequences are aligned and shown. The receptor number is appended to the three-letter species code. For *fgfr1* sequences, A and B are appended to the end. The human (Hsa) activation loop sequence for *fgfr1* is included on the bottom row. The syngnathid *fgfr1b* sequences are highlighted in purple, and the syngnathid *fgfr1b* R to P substitution is noted with an asterisk.

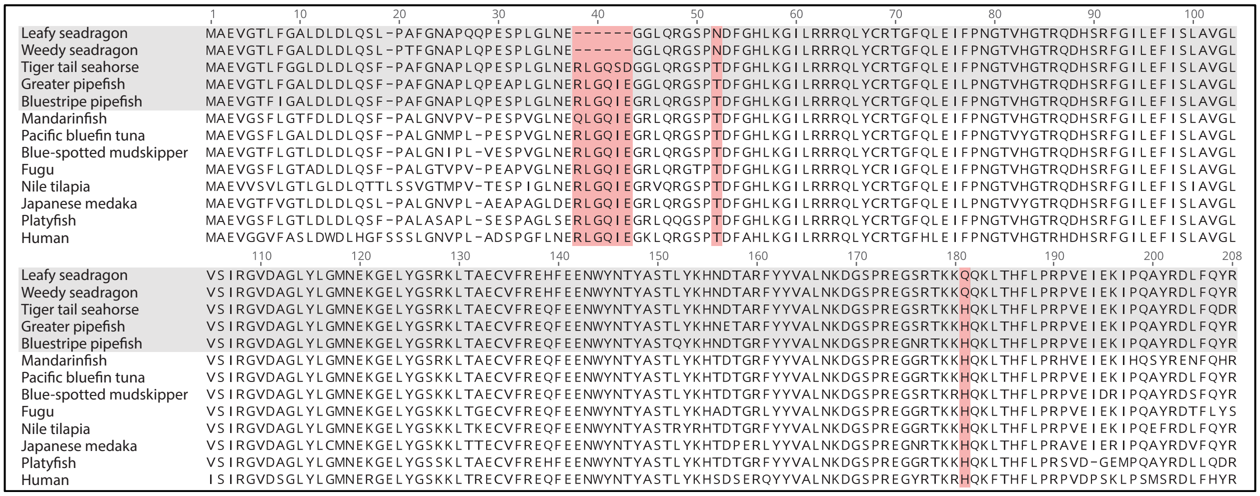

Figure S16. An alignment of predicted Fgf16 protein sequences reveals a deletion of six deeply conserved amino acids and substitutions of two other conserved amino acids (shaded in red) in the seadragons. These differences are not shared by the other syngnathids (clade shaded in grey), though seahorse has two substitutions in the 6 amino acid motif.

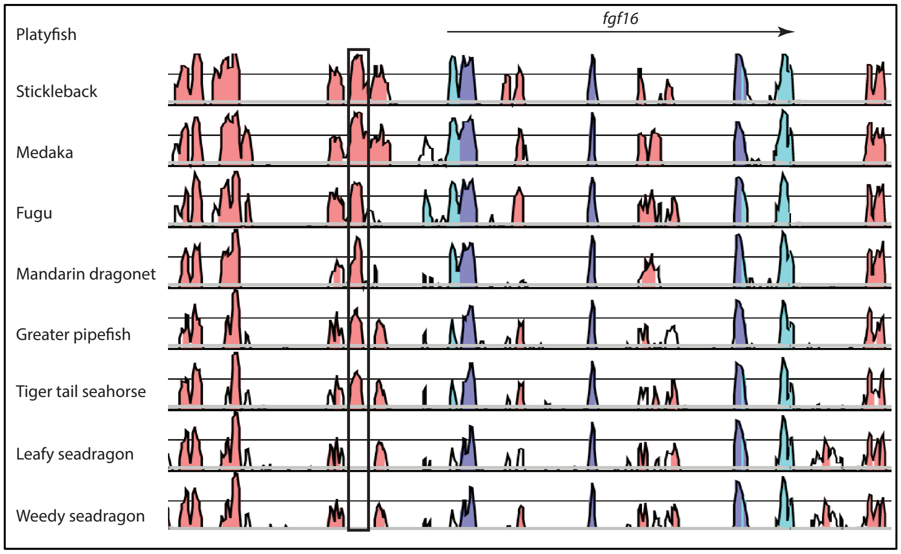

Figure S17. Leafy and weedy seadragons have lost a conserved non-coding element (shown in the black box) near *fgf16* that is retained in tiger tail seahorse, greater pipefish, and other percomorph fish. The VISTA plot highlights conserved non-coding sequences in pink, conserved UTRs in light blue, and conserved exons in dark blue.

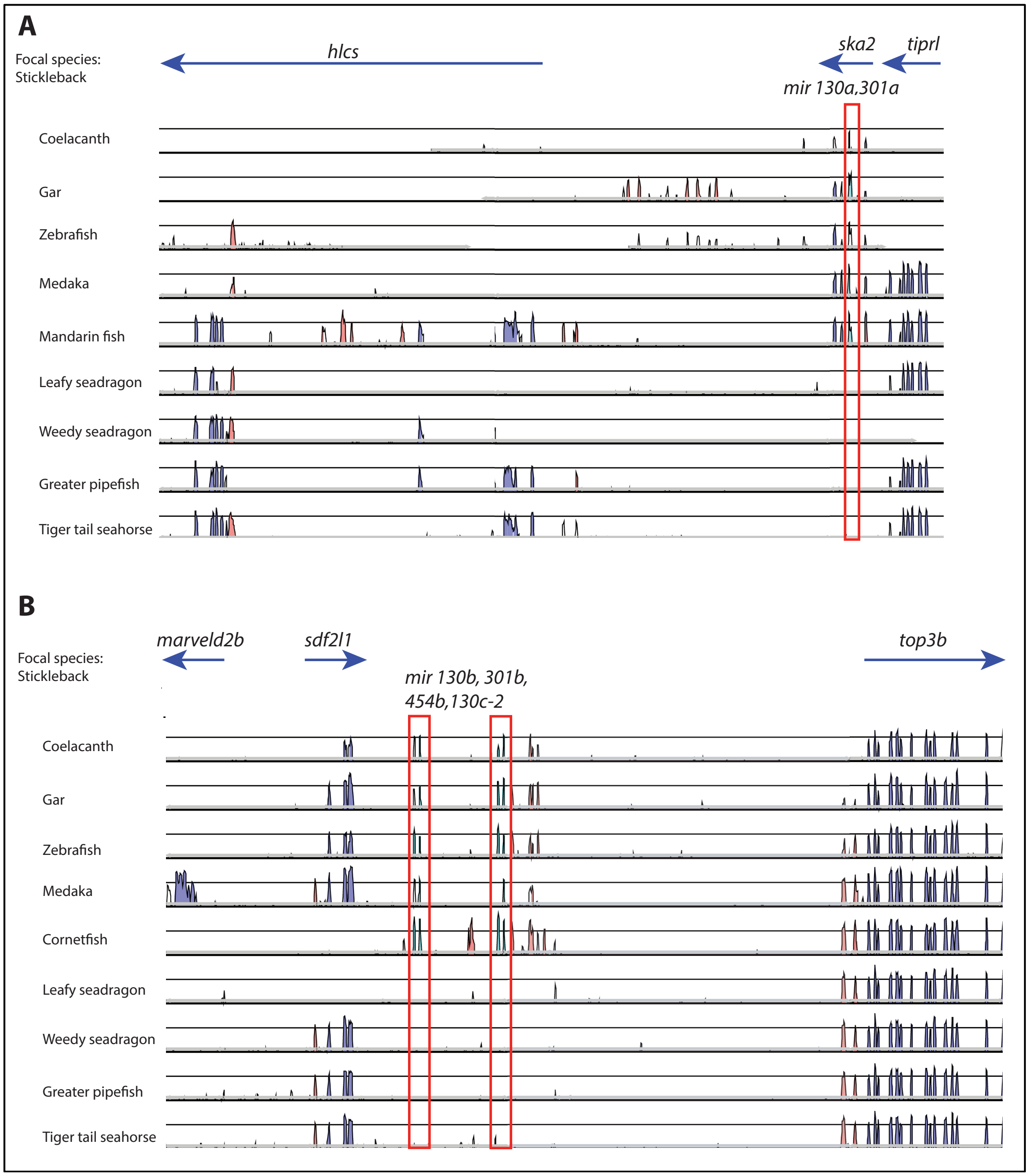

Figure S18. Two conserved miRNA clusters appear to be lost in the syngnathid lineage, clusters containing A) *mir130a (*also including *mir301a)* and B) *mir130b* (additionally having *mir301b*, *mir130c-2*, and *mir454b*). VISTA plots using threespine stickleback as the reference illustrate these losses (boxed in red). In these plots, conserved exons are demonstrated in dark blue peaks, conserved non-coding elements are noted in pink peaks, and conserved miRNAs are shown in light blue peaks. The orientation (blue arrows) and names of protein coding genes are shown above the plot. Syngnathids have further lost *ska2*, the protein coding gene in whose intron *mir130a* and *mir301a* normally reside (A).

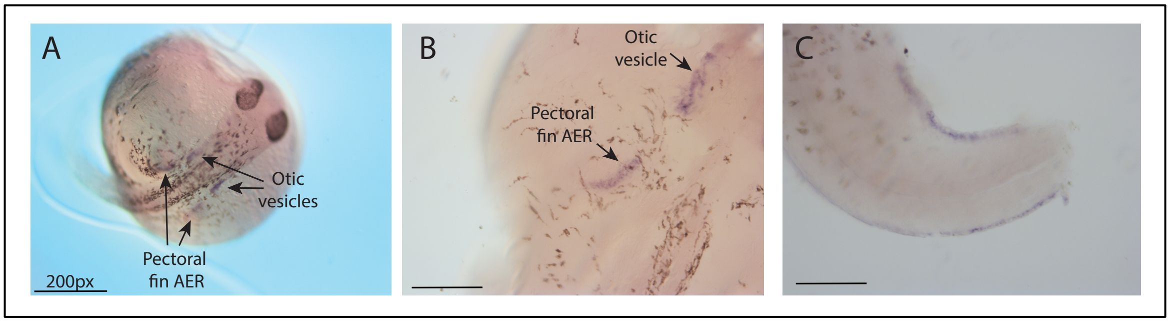

Figure S19. We confirmed that *fgf16* is expressed in a percomorph fish in the fin bud AER, as had been described in a phylogenetically distant taxon, zebrafish. Shown is the expression in developing threespine stickleback (*Gasterosteus aculeatus*; 75 hours post fertilization). Panels A) and B) show the expression domains in the marginal tissue of the fin bud and in the developing ear. C) The caudal fin margin also expresses the gene. The anti-sense RNA probe was transcribed from a cloned fragment amplified from stickleback genomic DNA using these primer sequences: forward primer 5' CGGACGAACTGGTTACTACATC3' and reverse 5' TTCATCCCTCGGACAGTCTTC3'.

SUPPLEMENTAL TABLES:

	fgf3	*fgf7*	*fgf10a*	*fgf10b*	*fgf22*	*fgf4*	*fgf4-like*	*fgf5*	*fgf6a*	*fgf6b*	*fgf8a*	*fgf8b*	*fgf17*	*fgf24*	*fgf16*	*fgf20a*	*fgf20b*	*fgf19*
Leafy Sea-dragon	-	+	+	+	+	-	-	+	+	+	+	+	-	+	+	+	+	+
Tiger tail Seahorse	-	+	+	+	+	-	+	+	+	+	+	+	+	+	+	+	+	+
Greater Pipefish	-	+	+	+	+	-	-	-	+	+	+	+	+	+	+	+	+	+
Bluestripe Pipefish	-	+	+	+	+	-	+	+	+	+	+	+	-	+	+	+	+	-
Platyfish	+	+	+	+	+	+	+	+	+	+	+	+	+	+	+	+	+	+

Table S1. Several developmentally important, conserved FGF ligands have been lost in syngnathid genomes. Shown in this table are the gene complements for focal FGF subfamilies, from leafy seadragon, tiger tail seahorse, greater pipefish, and bluestripe pipefish, as well as from a percomorph outgroup to the syngnathids: platyfish. Alternating column shading denotes gene subfamily groupings (1, 2).

**Species**	**Intact cluster**	**Description of split**
Amazon molly (*Poecelia formosa*)	Yes	
Ballan wrasse (*Labrus bergylta*)	Yes	
Barramundi perch *(Lates calcarifer*)	Yes	
Bicolor damselfish (*Stegastes partitus*)	Yes	
Blue tilapia (*Oreochromis aureus*)	Yes	
Blunt-snouted clingfish (*Gouania willdenowi*)	Yes	
Burton's mouthbrooder (*Haplochromis burtoni*)	Yes	
Channel bull blenny (*Cottoperca gobio*)	Yes	
Chinese medaka (*Oryzias sinensis*)	Yes	
Climbing perch (*Anabas testudineus*)	Yes	
Clown anemonefish (*Amphiprion ocellaris*)	Yes	
Eastern happy (*Astatotilapia calliptera*)	Yes	
European seabass (*Dicentrarchus labrax*)	Yes	
Fugu (*Takifugu rubripes*)	Split	*fgf3*, *fgf4*, *fgf19* all disbursed
Gilthead seabream (*Sparus aurata*)	Yes	
Greater amberjack (*Seriola dumerili*)	Yes	
Guppy (*Poecilia reticulata*)	Yes	
Indian glassy fish (*Parambassis ranga*)	Yes	
Indian medaka (*Oryzias melastigma*)	Yes	
Japanese medaka HSOK (*Oryzias latipes*)	Yes	
Javanese ricefish (*Oryzias javanicus*)	Yes	
Jewelled blenny (*Salarias fasciatus*)	Split	*fgf3/4* separated from *fgf19* by a small number of genes
Large yellow croaker (*Larimichthys crocea*)	Yes	
Live sharksucker (*Echeneis naucrates*)	Yes	
Lumpfish (*Cyclopterus lumpus*)	Yes	
Lyretail cichlid (*Neolamprologus brichardi*)	No	many assembly gaps adjacent to *fgf4/19*; *fgf3* missing
Makobe Island cichlid *(Pundamilia nyererei*)	Split	assembly break between *fgf3/4* and *fgf19*
Mangrove rivulus (*Kryptolebias marmoratus*)	Yes	
Midas cichlid (*Amphilophus citrinellus*)	Yes	
Monterrey platyfish (*Xiphophorus couchianus*)	Yes	
Mummichog (*Fundulus heteroclitus*)	Yes	
Nile tilapia (*Oreochromis niloticus*)	Split	*fgf3/4* separated from *fgf19*
Ocean sunfish (*Mola mola*)	Yes	
Orange clownfish (*Amphiprion percula*)	Yes	
Orbiculate cardinalfish (*Sphaeramia orbicularis*)	Yes	
Periophthalmus magnuspinnatus	Split	*fgf3*, *fgf4*, *fgf19* individually present on three short scaffolds
Pike-perch (*Sander lucioperca*)	Yes	
Platyfish (*Xiphophorus maculatus*)	Yes	
Round goby (*Neogobius melanostomus*)	Split	*fgf3/4* and *fgf19* on different short scaffolds
Sailfin molly (*Poecilia latipinna*)	Yes	
Sheepshead minnow (*Cyprinodon variegatus*)	Split	*fgf3/4/19* present but assembly broken in the middle of *fgf4*
Shortfin molly (*Poecilia mexicana*)	Split	*fgf3/4* and *fgf19* on different short scaffolds
Siamese fighting fish (*Betta splendens*)	Yes	
Spiny chromis (*Acanthochromis polyacanthus*)	Yes	
Stickleback (*Gasterosteus aculeatus*)	Yes	
Swamp eel (*Monopterus albus*)	Yes	
Tiger tail seahorse (*Hippocampus comes*)	No	*fgf3* and *fgf4* are missing

Tetraodon (*Dichotomyctere nigroviridis*)	Split	*fgf3* and *fgf4* separated, partial blast hit for *fgf19* near *fgf3*
Tongue sole (*Cynoglossus semilaevis*)	Yes	
Turbot (*Scophthalmus maximus*)	Yes	
Turquoise killifish (*Nothobranchius furzeri*)	Split	*fgf3*, *fgf4*, *fgf19* individually present on three short scaffolds
Western mosquitofish (*Gambusia affinis*)	Yes	
Yellowtail amberjack (*Seriola lalandi dorsalis*)	Yes	
Zebra mbuna (*Maylandia zebra*)	Yes	
Zig-zag eel (*Mastacembelus armatus*)	Yes	
Asian bonytongue (*Scleropages formosus*)	Yes	
Atlantic cod (*Gadus mohua*)	Yes	
Atlantic herring (*Clupea harengus*)	Split	*fgf3/4* separated from *fgf19*
Atlantic salmon (*Salmo salar*)	Yes	
Blind barbel (*Sinocyclocheilus anshuiensis*)	Yes	
Brown trout (*Salmo trutta*)	Yes	
Channel catfish (*Ictalurus punctatus*)	Yes	
Chinook salmon (*Oncorhynchus tshawytscha*)	Yes	
Coho salmon (*Oncorhynchus kisutch*)	Yes	
Common carp (*Cyprinus carpio*)	Yes	
Denticle herring (*Denticeps clupeoides*)	Yes	
Electric eel (*Electrophorus electricus*)	Yes	
Golden-line barbel (*Sinocyclocheilus grahami*)	Yes	
Goldfish (*Carassius auratus*)	Yes	
Horned golden-line barbel (*Sinocyclocheilus rhinocerous*)	Yes	
Huchen (*Hucho hucho*)	Split	*fgf3/4* separated from *fgf19*
Mexican tetra (*Astyanax mexicanus*)	Yes	
Northern pike (*Esox lucius*)	Yes	
Pachon cavefish (*Astyanax mexicanus*)	Split	*fgf3/4/19* present but assembly broken in the middle of *fgf4*
Paramormyrops kingsleyae	Yes	
Pinecone soldierfish (*Myripristis murdjan*)	Yes	
Rainbow trout (*Oncorhynchus mykiss*)	Yes	
Red-bellied piranha (*Pygocentrus nattereri*)	Yes	
Reedfish (*Erpetoichthys calabaricus*)	Yes	
Spotted gar (*Lepisosteus oculatus*)	Yes	
Zebrafish (*Danio rerio*)	Yes	

Table S2. A survey of the *fgf3/4/19* complements from ray-finned fish species with genomes currently available in the Ensembl database. The percomorph fishes, with a radiation time of about 128 Mya, are shaded in grey. The most basally branching Actinopterygian lineage represented, reedfish, diverged about 386 Mya (dates listed in Ensembl). Aside from Tiger tail seahorse, only one other assembly is missing any of the three genes. Lyretail cichlid is missing *fgf3*, but its assembly has sequence gaps adjacent to *fgf4*, and several closely related species of cichlid have intact clusters.

Gene	Branch.Tested	Likelihood.Ratio.Test	P.value	Adjusted.P.value	Sites.Detected.Under.Positive.Selection	adjustP
fgfrl1a	syngnathid	1.70	0.19	1.00		1.00
fgfrl1b	syngnathid	0.00	1.00	1.00		1.00
fgf8a	syngnathid	0.00	1.00	1.00		1.00
fgf8b	syngnathid	0.00	1.00	1.00		1.00
fgf10a	syngnathid	0.00	1.00	1.00		1.00
fgf10b	syngnathid	0.03	0.86	1.00		1.00
fgf19	syngnathid	0.00	1.00	1.00		1.00
fgfr1a	syngnathid	0.00	1.00	1.00		1.00
fgfr1b	syngnathid	0.07	0.79	1.00		1.00
fgfr2	syngnathid	0.00	0.97	1.00		1.00
fgfr3	syngnathid	0.00	1.00	1.00		1.00
fgfr4	syngnathid	0.00	1.00	1.00		1.00
fgf17	syngnathid	11.12	0.00	0.02	18, 19, 20, 60, 120	0.03
fgf6b	syngnathid	4.70	0.03	0.79	25,161,170	0.85
fgf6a	syngnathid	0.00	1.00	1.00		1.00
fgf20a	syngnathid	0.00	1.00	1.00		1.00
fgf20b	syngnathid	0.00	1.00	1.00		1.00
fgf4-like	syngnathid	0.00	1.00	1.00		1.00
fgf5	syngnathid	0.00	1.00	1.00		1.00
fgf22	syngnathid	0.00	1.00	1.00		1.00
fgf7	syngnathid	0.00	1.00	1.00		1.00
fgf24	syngnathid	0.00	1.00	1.00		1.00
fgfr1a	Peq/Hco	0.00	1.00	1.00		1.00
fgfr1b	Peq/Hco	5.72	0.02	0.45	3,35,47,52,56,90,97,125,127,128,129,137,170,293,355,406,407	0.49
fgf20a	Peq/Hco	0.00	1.00	1.00		1.00
fgf20b	Peq/Hco	0.00	1.00	1.00		1.00
fgf16	Peq	0.00	1.00	1.00		1.00
fgf6b	Peq	0.00	1.00	1.00		1.00
fgf6a	Peq	0.00	1.00	1.00		1.00
fgf16	Hco	0.00	1.00	1.00		1.00

Table S3. Subtle evidence for positive selection on FGF ligands and receptors in syngnathid fishes is revealed by phylogenetic analysis by maximum likelihood (PAML). Included are PAML branch site model results for each gene and the foreground branches tested. *fgf17* and *fgf6b* show potential signatures of positive selection in the lineage leading to syngnathids, and *fgfr1b* independently in lineages leading to leafy seadragon and tiger tail seahorse, although two of these fall within the range of false discovery after multiple testing is considered.

SUPPLEMENTAL DATASET CAPTIONS (See individual spreadsheet files):

Dataset S1. The 20 most abundant repeat clusters in leafy and weedy seadragon genomes, as measured by basepair proportion of ungapped genome length. Included are the cluster IDs, % abundance values, abundance ranks, consensus class, and total bp in seadragon genomes (both species) and the remaining taxa, for each repeat cluster. Clusters highlighted in orange and green loaded heavily on major PCA axes differentiating seadragons from other teleosts, based on basepair- and count-wise abundance measures, respectively.

Dataset S2. Statistically significant expansions/contractions of gene families from the CAFE 5 analysis. Sheet A) The 290 rapidly evolving gene families identified by the CAFE 5 analysis, including KO information and the top blast hit representative of each family. Branches of interest from the analysis, including all terminal syngnathiform branches, the seadragon ancestral branch, and the syngnathid ancestral branch, are included here. Gene families statistically significantly expanded/contracted along

the terminal seadragon branches, the ancestral seadragon branch, and the syngnathid ancestral branch are highlighted in red and blue, respectively. Numbers in branch columns indicate the estimated numbers of family members gained or lost (-) along each branch. Sheet B) Results from a KEGG pathway overrepresentation test for gene families expanded/contracted along the leafy seadragon branch, with the FDR controlled at 0.1. Pathways highlighted in green do not overlap with those significantly overrepresented in the list of “background” rapidly evolving gene families. Sheet C) KEGG pathway overrepresentation test results for expanded/contracted families in the weedy seadragon lineage. Sheet D) KEGG pathway overrepresentation test results for expanded/contracted families along the branch leading to seadragons. Sheet E) KEGG pathway overrepresentation test results for expanded/contracted families along the branch leading to syngnathids.

Dataset S3. Putative *copb2* duplicate regions in leafy and weedy seadragon genomes based on tblastn searches using 2 *X. maculatus* (paralog) queries. Sheet A) Regions of the leafy seadragon genome with one or more high-scoring pairs from the blast search. Sheet B) Similar information as in Sheet A, except that overlapping hits resulting from the two *X. maculatus* paralogs have been merged to eliminate redundancy, and coordinates have been adjusted to include 1 kb of flanking sequence on each side when possible. Sheets C-D) Information as in Sheets A-B, but for weedy seadragon. One of the *copb2* paralogs (on *P. eques* scaffold 13 and *P. taeniolatus* scaffold 14) is likely orthologous to the *X. maculatus copb2* paralog on chromosome (Xmac 6), and the others are possibly derived from this sequence. Echoing one of our other findings - that the fgf3/4/19 locus could harbor some inherent structural instability in the syngnathid lineage - leafy seadragon sequences for *copb2* fall in three locations on scaffold 2, in a region that also harbors a set of genes (e.g., *selenos*, *chsy1*, *snrpa1*) whose orthologs are adjacent to the *fgf3/4/19* cluster region in outgroup percomorphs (e.g., platyfish and threespine stickleback), but in syngnathids fall many megabases away from the remains of the cluster.

Dataset S4. Statistical enrichment test results for elevated repeat density in *cop2b* regions (leafy seadragon genome) and in seven syngnathid-specific gene loss regions (leafy seadragon and common dragonet genomes). Columns include the repeat class tested, the observed and randomly resampled among-region means, 95% confidence intervals for the resampled means, the ratio of observed to randomly resampled means (“density fold change”), and test p-values (original and FDR-adjusted). Clusters significantly enriched (FDR=0.1) in focal regions relative to randomly sampled regions are highlighted in green. Repeat classes with observed mean regional densities > 10% are in red font. Sheet A) Enrichment test results for *copb2* regions with 10 kb flanks. Sheet B) Enrichment test results for *copb2* regions with 1 kb flanks. Sheet C) Enrichment test results for common dragonet regions orthologous to the “seven gene loss” regions in syngnathids. Sheet D) Enrichment test results for the “seven gene loss” regions in the leafy seadragon genome. Sheet E) Enrichment test results for the *BovB* class of LINEs, among a test panel of 20 gene families from the leafy seadragon genome. The three evolutionary categories for these 20 gene families are in the second column. Note that the adjusted *p*-values were calculated in light of the 152 repeat classes tested. Sheet F) Enrichment test results for the *Tc1* family of DNA transposons. Columns are as described for Sheet E. Sheet G) Total repeat enrichment test results among the test panel of 20 gene families from the leafy seadragon genome, and additional information for these families, including blast annotation, evolutionary categories, etc. In this case adjusted *p*-values were calculated in light of the 20 different tests.

Dataset S5. Annotations of miRNAs from leafy seadragon (peq) and weedy seadragon (pta) genomes, based on Illumina miRNA-seq data. The names, confidence in correspondence with other teleost miRNA annotations, locations, and sequences for the miRNAs are listed.

REFERENCES

1. Itoh N, Konishi M. The zebrafish fgf family. Zebrafish. 2007;4(3):179-86.
2. Ornitz DM, Itoh N. The Fibroblast Growth Factor signaling pathway. Wiley Interdiscip Rev Dev Biol. 2015;4(3):215-66.
3. Durand NC, Robinson JT, Shamim MS, Machol I, Mesirov JP, Lander ES, et al. Juicebox Provides a Visualization System for Hi-C Contact Maps with Unlimited Zoom. Cell Syst. 2016;3(1):99-101.
4. Mendes FK, Vanderpool D, Fulton B, Hahn MW. CAFE 5 models variation in evolutionary rates among gene families. Bioinformatics. 2020.
5. Choi Y, Chan AP. PROVEAN web server: a tool to predict the functional effect of amino acid substitutions and indels. Bioinformatics. 2015;31(16):2745-7.

<u>Supplementary Methods</u>

LEAFY AND WEEDY SEADRAGON GENOMES CONNECT GENIC AND REPETITIVE DNA FEATURES TO THE EXTRAVAGANT BIOLOGY OF SYNGNATHID FISHES

Clayton M. Small, Hope M. Healey, Mark C. Currey, Emily A. Beck, Julian Catchen, Angela S. P. Lin, William A. Cresko, and Susan Bassham

Seadragon genome assembly

We isolated genomic DNA from the leafy appendage of a single adult male leafy seadragon (*Phycodurus eques*) provided by the Birch Aquarium at Scripps Institution of Oceanography, and from the liver of a single adult female weedy (common) seadragon (*Phyllopteryx taeniolatus*) provided by the Tennessee Aquarium. Both animals were being euthanized (because of terminal decline in health) by their respective husbandry teams, and tissues were preserved by either flash-freezing at -80C or storage in RNAlater Stabilization Solution (Invitrogen) after equilibration at 8C.

We manually pulverized approximately 100 mg of frozen tissue using Covaris tissueTUBE TT1 (SKU 520001) cryosafe bags, and a dry-ice chilled hammer and block. High-molecular-weight genomic DNA was purified from the powdered tissue using the magnetic pulldown-based Circulomics Nanobind Tissue Big DNA Kit (SKU NB-900-701-01), and we submitted 7.4 µg of leafy seadragon and 9 µg of weedy seadragon genomic DNA for PacBio sequencing at the University of Oregon Genomics Core Facility (UO_GC3F). There, genomic DNA was sheared to 60 kb size with a Megaruptor 2, resulting in post-shear average size of 37.1 kb for leafy and 36.3 kb for weedy. PacBio libraries were generated for each via the CLR method at 2x reaction scale with 6.7 µg of sheared DNA for leafy, and at 1x reaction scale with 4.6 µg of sheared DNA for weedy. Post-library production average size was 41.0 kb for leafy and 42.5 kb for weedy. Libraries were size-selected with a BluePippin using the high-pass method with

Marker U1 and a 25 kb lower cutoff for leafy and a 35 kb lower cutoff for the weedy. Post-selection average size was 51.5 kb for leafy and 45.7 kb for leafy. The leafy library was sequenced on a PacBio Sequel I in four SMRT Cells (1M) at 5 pM loading concentration, with Binding Kit 3.0, Sequencing Kit 3.0, primer v4, and internal control 3.0. The weedy library was sequenced on a PacBio Sequel II in one SMRT Cell (8M), at 15 mM loading concentration, with Binding Kit 1.0, Sequencing Kit 1.0, primer v4, and internal control 1.0. Total throughputs were 49.12 gb for leafy and 80.80 gb for weedy.

We constructed shotgun Illumina libraries using DNA from the same *P.* eques individual used for the PacBio data, and a different male from the same tank as the PacBio fish, for *P. taeoniolatus*. These shotgun libraries were constructed at different times, using slightly different methods. We sonicated 150 ng of leafy and 1.34 µg of weedy genomic DNA with a Covaris sonicator, targeting a shear peak at 400 bp, and sheared DNA was end-repaired, A-tailed, and ligated to Illumina-compatible adapters by standard enzymatic methods. The final leafy seadragon library was generated with 10 PCR cycles and fragments between 500 and 700 bp were selected by slab gel electrophoresis. The final weedy seadragon library was generated with 12 PCR cycles, and 340 to 390 bp fragments were selected by capillary electrophoresis via a Blue Pippin apparatus (Sage Science). We sequenced the short-insert libraries in one flowcell lane on an Illumina HiSeq4000 at the UOGC3F.

To assemble each genome we used Flye (1) with all PacBio data (excluding "scraps") with default parameters and a genome size estimate of 600 Mb. *For a list of all software used in this paper, and the versions used, see section below*. We performed two rounds of polishing on the primary Flye assemblies with the tool arrow (2) using the PacBio reads aligned to the assemblies with the pbmm2 wrapper for minimap2 (3). We then performed an additional two rounds of polishing for each genome with "shotgun"

whole-genome sequencing (WGS) Illumina data, using pilon (4), in “basic corrections mode,” aligning the reads to their respective assembly using bwa-mem (5). We also used the raw WGS data to estimate genome sizes and heterozygosity for both seadragon species with GenomeScope (6) which is based on k-mer frequency spectra models.

Hi-C scaffolding

Illumina-compatible Hi-C libraries were produced (from the same individuals used for PacBio sequencing) from liver (*P. taeniolatus*) and from appendage (*P. eques*), tissue using Hi-C construction kits (Animal Proximo v1 and v2, respectively; Phase Genomics). We sequenced the Hi-C libraries in 1 lane on an Illumina HiSeq4000 at the UOGC3F, generating 218.76 million and 150.99 million paired-end 150 nt reads for *P. eques* and *P. taeniolatus*. We used juicer (7) to align reads to the polished genome assemblies and construct Hi-C contact maps, and the resulting “merged_nodups.txt” files to order and orient Flye contigs/scaffolds into putative chromosomes using select steps from the 3D-DNA pipeline (8). Specifically, we ran generate-sorted-cprops-file.awk to obtain cprops files, followed by scaffolding with run-liger-scaffolder.sh. This prevented any automated breaking of original Flye contigs/scaffolds. We then ran run-asm-visualizer.sh from the pipeline to produce files suitable for visualization and manual editing in juicebox (9). Minimal manual editing was performed in juicebox: limited to movement or inversion of whole Flye contigs/scaffolds, except for a single case in which a leafy seadragon Flye scaffold was split to preserve a strong contact signal from the Hi-C data.

mRNA-seq

We used the same two individuals as for PacBio genomic sequencing for the source material for RNA-seq. From the *P. eques* specimen, testis, leafy appendage, eye, and gill tissues were sampled. From *P. taenoiolatus* ovary, leafy appendage, eye, and liver tissue were sampled. All tissues were equilibrated with RNAlater Stabilization Solution (Thermofisher) at 8 C for 24 hours and stored at -80 C. We harvested total RNA from ~20 mg portions of each tissue via organic extraction in Trizol followed by column-binding procedures (10). Indexed sequencing libraries for mRNA-seq were generated for each tissue using the Roche KAPA RNA HyperPrep Kit (SKU 08098115702), which includes an initial polyA+ RNA selection step. All libraries were sequenced in a single Illumina sequencing lane on a HiSeq4000 at UO_GC3F to obtain 301.52 million passing paired-end 100 nt reads (Pequ testis: 34.22 million; Pequ appendage: 34.38 million; Pequ eye: 40.66 million; Pequ gill: 40.03 million; Ptae ovary: 40.30 million; Ptae appendage: 27.81 million; Ptae eye: 34.93 million; Ptae liver: 49.19 million). We demultiplexed, Illumina quality-filtered, and adaptor-trimmed all mRNA-seq reads using process_shortreads from the Stacks software suite (11, 12).

miRNA-seq

From the same tissues used to generate the mRNA-seq data, we constructed indexed sequencing libraries using the NextFlex Small RNA-seq Kit v3. These were sequenced at UO_GC3F in a single lane on a HiSeq4000 to generate 38.38 million (leafy) and 27.68 million (weedy) passing paired-end 100 nt reads (Pequ testis: 7.08 million; Pequ appendage: 5.69 million; Pequ eye: 8.13 million; Pequ gill: 12.23 million; Pequ skin: 5.26 million; Ptae ovary: 7.31 million; Ptae appendage: 8.67 million; Ptae eye: 11.70 million). We demultiplexed, and Illumina quality-filtered as stated above, then used cutadapt (13) to remove adaptor and 4-nt randomer sequences from the miRNA-

seq reads. Finally, we merged the overlapping paired-end reads into single sequences using BBMerge (14).

The seadragon genomes were formatted as databases with BBMap (15). Each seadragon genome was annotated by supplying miRNA-seq reads to Prost! (16). We supplied Prost! With hairpin and mature miRNA sequences from Antarctic black icefin (*Chaenocephalus aceratus*), European perch (*Perca fluviatilis*), threespine stickleback (*Gasterosteus aculeatus*), platyfish (*Xiphophorus maculatus*), zebrafish (*Danio rerio*), gar (*Lepisosteus oculatus*), medaka (*Oryzias latipes*) (from a custom database provided by Thomas Desvignes). We also provided Prost! with noncoding RNAs from threespine stickleback (BROAD S1; 104.1 database). To confirm the absence of miRNAs, we used blastn (default parameters) and mVISTA analysis (through the mVISTA website with the shuffle lagan algorithm, translation anchoring, and default parameters).

Genome annotation

We aligned all processed mRNA-seq reads from seadragon libraries to both the leafy and weedy seadragon genome assemblies using STAR (17). We also processed and aligned (using identical tools and options) publicly available RNA-seq data (from *S. scovelli* and *S. typhle*) to the *Syngnathus acus* genome, (from *D. excisus* and *Microphis manadensis*) to the *Doryrhamphus excisus* genome, (from *Foetorepus agassizii* and *Repomucenus calcaratus*) to the *Synchiropus splendidus* genome, and (from *T. orientalis*, *T. maccoyii*, *T. albacares*, and *T. thynnus*) to the *Thunnus orientalis* genome. See below for accession information.

Prior to annotation, each genome was soft-masked using RepeatMasker (18) based on teleost-specific sequences in the RepeatModeler2 (19) library and a genome-specific library generated by the RepeatModeler2 pipeline. To annotate each soft-

masked genome based on the mRNA-seq alignments alone, we used Braker2 (20). We evaluated the Braker2 set of annotated genes for each genome using InterProScan (21) and retained for a final, “filtered” version of the annotation if they showed InterProScan evidence other than disorder-based (i.e. MobiDB-lite). We subjected remaining amino acid sequences to blastp searches against the NCBI RefSeq protein database and retained those in the filtered annotation if they returned a hit (e-value threshold of 0.001). In summary, gene annotations were excluded from the final annotations only if they 1. Failed to return a non-MobiDB-lite hit from InterProScan (21) analysis, and 2. Failed to return a blastp hit with an e-value < 0.001 after a search of the NCBI RefSeq protein database. For all downstream analysis of protein-coding sequences based on these and other annotations, we selected the longest transcript per locus.

Gene family evolution analysis

Based on the custom Braker genome annotations above and publicly available annotations from *Danio rerio, Astyanax mexicanus, Esox lucius, Gadus morhua, Boleophthalmus pectinirostris, Hippocampus comes, Notothenia coriiceps, Gasterosteus aculeatus, Dichotomyctere nigroviridis* (formerly called *Tetraodon nigroviridis*) *Oreochromis niloticus, Maylindia zebra, Oryzias latipes, Nothobranchius furzeri, Poecilia reticulata*, and *Xiphophorus maculatus* (see sequence accession section below), we retrieved amino acid sequences from the longest transcript of each locus, then performed all-by-all blastp (22). We clustered the 503,304 sequences into putative gene families using mcl (23). We ran mcl four times, each time with the inflation parameter set at 2, 3, 4, and 5, respectively, ultimately deciding on 4 as the optimal value to maximize species contributions to clusters while minimizing the total number of clusters.

Next we performed a series of gene family evolution analyses using CAFE 5 (24). We separated especially large gene families (N=22) from the remaining 13,150 (our "filtered" set) to minimize the influence of especially large families on parameter estimates, in accordance with suggestions in the CAFE manual. We used the filtered set of gene families and time-calibrated phylogenetic relationships for the 21 species from Rabosky et al. (25) to construct an error model for all subsequent CAFE analyses. We used CAFE and the 21-species tree to fit a single-lambda model for both filtered and large family sets, repeating each to check for convergence. The CAFE output from the initial single-lambda models was used to identify families that were likely the product of branch-specific expansion or contraction. All families with evidence for non-uniform gene family evolution, the subset of these with evidence for leafy and weedy seadragon branch-specific expansion/contraction, the subset with evidence for internal seadragon branch-specific expansion/contraction, and the subset with evidence for internal syngnathid branch-specific expansion/contraction, were tested for overrepresentation of KEGG pathways using the R package ClusterProfiler (26). The KEGG orthology (KO) annotations required for these analyses were obtained via the KAAS server (27): accessed on 10/23/2020 using the single-direction blast algorithm, with template genome codes hsa, dme, cel, ath, sce, eco, nme, rpr, bsu, cac, mtu, mmu, gga, xla, dre, amex, tng, ncc, mze, onl, ola, xma, pret, nfu, hcq, bpec, els, syc, and mja.

For the filtered set we also fit three multi-lambda models (seadragon branch-specific + background; syngnathid branch-specific + background; seadragon branch-specific + syngnathid branch-specific + background), and we evaluated their likelihood over the single-lambda model via 100 CAFE simulations of gene family evolution based on the root distribution of family sizes from the observed data.

Transposable element identification and analysis

We characterized the repetitive content of 16 teleost genome assemblies (see Sequence Accessions below), with a nearly exclusive focus on assemblies produced by long-read (i.e., PacBio or Oxford Nanopore) and/or linked-read (i.e., 10x Genomics) technologies. We identified repeats de novo for each assembly using RepeatModeler2 (19) and TransposonPSI (28). The TransposonPSI repeats identified in each genome were aligned using MAFFT (29) and clustered using Ninja (30) to reduce redundancy. We combined all of the de novo repeat predictions across species with the teleost library from RepeatMasker (18) and all sequences from the FishTEDB (31). Each of these sequences was classified using RepeatClassifier from RepeatModeler, and all sequences were clustered at a 0.80 identity threshold using USEARCH (32). This permitted use of a large, single repeat library to characterize all 16 genomes in a comparable framework. We masked each genome using this large library with RepeatMasker (18) and integrated the aforementioned RepeatClassifier and cluster status annotations into the RepeatMasker output.

We used both the RepeatClassifier repeat classes and the USEARCH cluster membership from the final, integrated RepeatMasker .gff files to quantify and characterize the distribution of repeat categories across genomes and ordinate species genomes in repeat space, using custom scripts written using the R statistical language. These analyses included standard principal components analysis (PCA), relative abundance plots, and repeat densities plotted along chromosomes.

Finally, we implemented genome-wide random sampling (without replacement) of repeat densities in gene flanking regions to test hypotheses about elevated repeat abundance associated with specific gene families or groups. To generate a null "genomic background" distribution of repeat densities neighboring genes we randomly

sampled N annotated genes from the focal genome 499 times, where N = the number of genes in a group. For each gene in the random sample we calculated the proportion of basepairs intersecting annotated repeat classes in the region extending from the end of the gene upstream of the sampled gene to the beginning of the gene downstream of the sampled gene. The mean repeat densities across all sampling bouts were then used as a null distribution against which to test extremeness of repeat density for a given focal group, such as gene family of interest. Those focal groups included both 1kb and 10kb flanking regions for blast-annotated copb2 sequences in the P. eques genome, seven regions of key developmental gene loss in P. eques (and corresponding regions of the C. lyra (common dragonet) genome), and a reference panel of 20 gene families representing three different evolutionary categories, tested in the P. eques genome.

We selected the 20-family reference panel to explore the possibility that enriched repeat density may be a more general feature of seadragon-expanded gene families, or gene families that have evolved rapidly in size in other ways. Specifically, we chose 1) five gene families with the strongest evidence for expansion in the ancestral seadragon lineage and blast evidence for homology with known proteins, 2) five families with the strongest evidence for contraction in the seadragon lineage and blast support, and 3) ten randomly selected gene families identified from the CAFE analysis as significantly expanded/contracted in non-syngnathid lineages and with blast support. We also required that these families contain at least two members. Our reasoning for choosing this test panel was to compare seadragon-expanded and -contracted genes with gene families rapidly evolving, but not in syngnathids.

For each member of the 20 gene families we calculated flanking repeat densities and compared repeat density distributions from those regions to null distributions from randomly sampled, equally-sized sets of genes (see above). This allowed us to test for

mean enrichment of repeat classes and summed (total) repeat densities at the gene family level. We also directly compared summed repeat densities among the three categories mentioned above by fitting a linear model in which within-family (i.e., among-gene) variation was accounted for as a random effect. To perform this analysis we used the lmer function of the lme4 R package and tested for an overall effect of gene family category using a Kenward-Roger F test (Anova function from the car R package with test=”F” and type=”III”), given the unbalanced nature of the design. R code examples for the repeat distribution analyses are included in the Dryad repository associated with this paper (doi:10.5061/dryad.31zcrjdmf).

Analyses of gene content and molecular evolution of the FGF and FGFR gene families in syngnathid fishes

We assessed gene content and sequence divergence in the FGF and FGFR gene families in four representative lineages of syngnathids: flagtail pipefishes (the most basal lineage), seadragons, seahorses, and *Syngnathus* pipefishes. For these analyses we considered published genome assemblies, annotations, and transcriptome data, and our newly annotated genome assemblies and RNA-seq data presented in this study. We used a combined strategy of BLAST, HMM-based genome scans for conserved domains using Wise2 (tps://www.ebi.ac.uk/~birney/wise2/), VISTA, and conserved synteny comparisons based on the above resources to determine presence or absence of genes. See sequence accessions below for the following ray-finned fish sequences used as FGF search queries: spotted gar, *Lepisosteus oculatus*; zebrafish, *Danio rerio*; threespine stickleback, *Gasterosteus aculeatus*; platyfish, *Xiphophorus maculatus*; Japanese medaka, *Oryzias latipes*; tigertail seahorse, *Hippocampus comes*. We also performed BLASTX searches of de novo-assembled transcripts from Gulf

pipefish (*S. scovelli*) embryo head and brood pouch RNA-seq data against seven NCBI RefSeq proteomes (*Homo sapiens*, *Danio rerio*, *Oryzias latipes*, *Xiphophorus maculatus*, *Gasterosteus aculeatus*, *Hippocampus comes*, *and Syngnathus acus*), to verify that *fgf4* and *fgf3* were absent from transcribed sequence data, in addition to their absence from genome assemblies. These two transcriptome assemblies are included in the Dryad repository for this paper and were generated from the RNA-seq data associated with NCBI BioProject PRJNA355893 as part of that study.

To test for positive selection, we aligned syngnathid coding sequences of select FGF and FGFR genes with their orthologs from several outgroup percomorph species: dragonet (mandarinfish, *Synchiropus splendidus* or common dragonet, *Callionymus lyra*), tuna (bluefin tuna, *Thunnus* orientalis or albacore tuna, *Thunnus alalunga*), mudskipper (blue-spotted mudskipper, *Boleophthalmus pectinirostris* or common mudskipper, *Periopthalmus magnuspinnatus*), pufferfish (fugu, *Takifugu rupripes*), cichlid (Nile tilapia, *Oreochromis niloticus*), Japanese medaka (*Oryzias latipes*), and platyfish (*Xiphophorus maculatus*). See sequence accessions below and supporting alignment files for sequences used.

We carried out branch-site tests for positive selection as described in Zhang et al. (33) using codeml in PAML. We supplied a species phylogenetic tree for each test; topology for the syngnathid clade was based on Longo et al. (34) and topology for outgroup taxa was based on Rabosky et al. (25) . In the PAML branch site-model test of positive selection, ω (omega) varies both among sites and among lineages (Model = 2 and NSites = 2). It assumes the phylogeny is divided *a priori* into foreground and background lineages; only the foreground lineage can experience positive selection. The model also assumes four different codon classes: (1) conserved throughout the phylogeny, (2) evolving neutrally throughout the phylogeny, (3) conserved on the

background lineages but positively selected on the foreground lineage, and (4) evolving neutrally on the background lineages and under positive selection on the foreground lineage. The null (neural) model allows sites to evolve under purifying selection on the background lineages and to be released from constraints to evolve neutrally on the foreground branches (fix_omega = 1 and omega = 1). The alternative model of positive selection is constrained to a fraction of sites with $\omega > 1$ on the foreground lineage (fix_omega = 0 and omega = 1.2). Fits of the neutral and selection models are then compared using a log-likelihood ratio test.

X-ray microscopic imaging of the weedy seadragon

A formalin-fixed male weedy seadragon from the Tennessee Aquarium (Chattanooga, TN) was imaged in segments with a Zeiss XRadia 620 Versa X-ray Microscope at the University of Oregon Knight Campus for Accelerating Scientific Impact's X-ray Imaging Core Facility, using the following settings to yield a voxel size of 54 µm: energy 80kV, power 10W, Objective 0.4X, Binning 2. Separate sections were scanned (4 wide-field scans and 2 vertical-stitch scans of 4 segments each), custom global reconstruction scaling factors for each scan were set to match the first scan performed, and Dragonfly Pro was used to register and stitch the scans to form a full specimen 3D reconstruction (Fig. 1; SI Appendix, Fig. S2). Specifically, the image segments were sequentially processed through Dataset Registration (coarse: rotation 2°, translation 110 µm; fine: rotation 0.4°, translation 11 µm) and 3D Stitching (parameters: weighted blending, nearest interpolation, most precise resolution) with Object Research Systems (ORS) Dragonfly Pro software (version 2020.2.0.094) in order to produce a composite full specimen 3D reconstruction at 54 µm voxel size. Regions of the head and cleithrum were additionally scanned at 17 µm voxel size (1 single

normal-field, 1 vertical-stitch normal-field scan of 3 segments; energy 80kV, power 10W, Objective 0.4X, Binning 1). These 17 μm scans - plus a patch from the 54 μm scan to cover a small wedge of missing data over the orbits - were stitched to generate a 3D reconstruction with Dragonfly (non-commercial license, version 2021.1; dataset registration parameters: coarse rotation of 10°, fine rotation of 0.4°, course translation of 30 mm, and fine translation of 3mm; stitching parameters: weighted blending, tricubic interpolation, and most precise resolution). After completing all scans of the unstained specimen, the fish was then dehydrated through an ethanol series and immersed in a contrast agent for visualization of soft tissues (11.25% Lugol's iodine (3.75% w/v of I_2 and 7.5% w/v of KI)) for 14 days. A segment of the rostral part of the Lugol's-stained tail, encompassing a pair of leafy appendages, was scanned at 27 μm voxel resolution (energy 80kV, power 10W, Objective 0.4X, Binning 2).

All 3D reconstructions were visualized using Dragonfly software (ORS non-commercial license, version 2021.1) and screenshots were taken. In generation of a composite image for Fig. 1 and SI Appendix, Fig. S2, we reflected a right lateral view of the seadragon - generating a virtual left side view to match presentation convention - because the left side gill of the specimen had been partially removed for necropsy at the Tennessee Aquarium. We additionally optimized contrast and brightness of the images with Photoshop (v22.4.3) and used the spot healing brush on one of the leafy appendages from the full body image to remove an artifactual line.

List of command-line software, with versions and any non-default parameters

flye (version 2.4.2) --pacbio-raw

PacBio Secondary Analysis Tools on Bioconda

pbmm2 (version 1.0.0) align

pbindex (version 0.23.0)

variantCaller (version 2.3.3) --algorithm arrow

bwa (version 0.7.17) mem

pilon (version 1.22)

GenomeScope (genomescope.R downloaded April 2019)

juicer (version BETA)

3D-DNA pipeline (version 180419)

generate-sorted-cprops-file.awk

run-liger-scaffolder.sh -p false -s 10000

run-asm-visualizer.sh -p false

Juicebox (version 1.9.8)

process_shortreads (mRNA-seq)

process_shortreads (version 2.2) --filter_illumina -r -D --adapter_mm 2 \

--adapter_1 AGATCGGAAGAGCACACGTCTGAACTCCAGTCAC \

--adapter_2 AGATCGGAAGAGCGTCGTGTAGGGAAAGAGTGT

process_shortreads (miRNA-seq)

process_shortreads (version 2.2) --filter_illumina -r -D

cutadapt (miRNA-seq; adaptor trimming)

cutadapt (version 1.18) --overlap 10 -a TGGAATTCTCGGGTGCCAAGG \

-A GATCGTCGGACTGTAGAACTCTGAAC --minimum-length 10

cutadapt (miRNA-seq; 4-nt randomer trimming, repeated for R1s and R2s)

cutadapt (version 1.18) -u 4 -u -4 --minimum-length 1

bbmerge.sh (version 8.82) mininsert=10 mininsert0=10 minoverlap=10

bbmap.sh (version 35.69) build=1 overwrite=true fastareadlen=500 k=7

prost (version 0.7.60) min_seq_count: 5 min_seq_length: 17 max_seq_length: 25 \

wiggle: 5 max_locations_to_report: 6

STAR (version 2.5.3) --outSAMtype BAM Unsorted

repeatmasker (version 4.1.1) -gff -nolow -norna -xsmall

repeatmodeler (version 2.10.0) -LTRStruct

braker.pl (version 2.1.4) --softmasking and --skip_fixing_broken_genes flags

interproscan.sh (version 5.27-66.0)

blastp (version 2.2.30+) -outfmt 7 -seg yes

mcl (version 14-137) -I 4

cafexp (version 5.0)

transposonPSI.pl (version 08222010)

RepeatClassifier (from RepeatModeler version 1.0.11)

mafft (version 7.471) --large --quiet

Ninja (version 0.97) --out_type c --corr_type m --cluster_cutoff 0.2

usearch (version 11.0) -cluster_fast -id 0.8 -sort length

codeml (PAML version 4.9)

List of accession IDs for publicly available sequence data

Publicly available (Ensembl and NCBI) genome annotations for gene family analysis

Danio_rerio.GRCz11.94.gff3

Astyanax_mexicanus.Astyanax_mexicanus-2.0.94.gff3

Esox_lucius.Eluc_V3.94.gff3

Gadus_morhua.gadMor1.94.gff3

GCF_000788275.1_BP.fa_genomic.gff

Hippocampus_comes.H_comes_QL1_v1.94.gff3

GCF_000735185.1_NC01_genomic.gff

Gasterosteus_aculeatus.BROADS1.94.gff

Tetraodon_nigroviridis.TETRAODON8.94.gff3

Oreochromis_niloticus.Orenil1.0.94.gff3

Maylandia_zebra.M_zebra_UMD2a.94.gff3

Oryzias_latipes.ASM223467v1.94.gff3

GCF_001465895.1_Nfu_20140520_genomic.gff

Poecilia_reticulata.Guppy_female_1.0_MT.94.gff3

Xiphophorus_maculatus.X_maculatus-5.0-male.94.gff3

mRNA-seq data for custom non-seadragon genome annotations

S. scovelli and *S. typhle* read SRA accession IDs for *S. acus* annotation

SRR10158924

SRR10158932

SRR10158938

SRR10158936

SRR5783122

SRR5783125

SRR5783119

SRR5071373

SRR5071374

SRR5071375

SRR5071380

SRR5071383

SRR5071384

SRR5783123

ERR3994083

ERR3994130

ERR3994136

ERR3994142

ERR3994155

ERR3994161

ERR3994211

ERR3994222

M. manadensis read SRA accession IDs for *D. excisus* annotation

SRR7640542

SRR7640531

SRR7640526

SRR7640533

SRR7640525

SRR7640534

F. agassizii and *R. calcaratus* read SRA accession IDs for *S. splendidus* annotation

SRR5997759

SRR5481263

SRR5481268

SRR5481273

SRR5481278

SRR5481283

SRR5481288

SRR5481293

SRR5481297

SRR5481302

SRR5481307

SRR5481311

SRR5481316

SRR5481321

SRR5481326

T. orientalis, *T. maccoyii*, *T. albacares*, and *T. thynnus* read SRA accession IDs for *T. orientalis* annotation

SRR8756175

SRR8756176

SRR8756177

SRR8756179

SRR8756180

SRR8756181

SRR8756183

SRR8756208

SRR8756213

SRR2079754

SRR2079756

SRR2079764

SRR2079768

SRR8242435

SRR1536265

SRR1536295

SRR1536891

SRR1536892

SRR1536893

Genome assemblies for repetitive DNA analysis

Phycodurus_eques	Pequ	pequ_v1.0.fasta (this study)
Phyllopteryx_taeniolatus	Ptae	ptae_v1.1.fasta (this study)
Syngnathus_acus	Sacu	GCA_901709675.2_fSynAcu1.2_genomic.fna
Doryrhamphus_excisus	Dexc	BP1_comb_355M_pseudohap.2.fasta (this study)
Callionymus_lyra	Clyr	SGN_Clyra_asm_hic.final.fasta (http://www.gigadb.org/dataset/100799)
Thunnus_orientalis	Tori	GCA_009176245.1_tuna2_genomic.fna.gz
Takifugu_rubripes	Trub	GCF_901000725.2_fTakRub1.2_genomic.fna
Pseudochanichthys_georgianus	Pgeo	GCA_902827115.1_fPseGeo1.1_genomic.fna
Oreochromis_niloticus	Onil	GCF_001858045.2_O_niloticus_UMD_NMBU_genomic.fna
Xiphophorus_maculatus	Xmac	GCF_002775205.1_X_maculatus-5.0-male_genomic.fna
Oryzias_latipes	Olat	GCA_004347445.1_ASM434744v1_genomic.fna
Periophthalmus_magnuspinnatus	Pmag	GCF_009829125.1_fPerMag1.pri_genomic.fna
Gadus_morhua	Gmor	GCF_902167405.1_gadMor3.0_genomic.fna
Esox_lucius	Eluc	GCF_011004845.1_fEsoLuc1.pri_genomic.fna
Danio_rerio	Drer	GCA_903684855.2_fDreTuH1.2_genomic.fna

FGF and FGFR molecular evolution analyses

Query FGF amino acid sequences for gene searches in genomes

Dre_fgf10a:ENSDARG00000030932:ENSDART00000048581.5

Dre_fgf10b:ENSDARG00000100475:ENSDART00000170921.3

Dre_fgf11a:ENSDARG00000069662:ENSDART00000144375.4

Dre_fgf11b:ENSDARG00000043907:ENSDART00000148831.2

Dre_fgf12a:ENSDARG00000027957:ENSDART00000028390.9

Dre_fgf12b:ENSDARG00000113525:ENSDART00000090596.4

Dre_fgf13a:ENSDARG00000035056:ENSDART00000010982.11

Dre_fgf13b:ENSDARG00000056633:ENSDART00000162710.3

Dre_fgf14:ENSDARG00000040982:ENSDART00000060051.5

Dre_fgf16:ENSDARG00000042233:ENSDART00000061928.6

Dre_fgf17:ENSDARG00000102778:ENSDART00000168252.2

Dre_fgf18a:ENSDARG00000088048:ENSDART00000123063.3

Dre_fgf18b:ENSDARG00000043962:ENSDART00000064545.6

Dre_fgf19:ENSDARG00000098663:ENSDART00000171864.2

Dre_fgf1a:ENSDARG00000017542:ENSDART00000005842.7

Dre_fgf1b:ENSDARG00000042811:ENSDART00000062836.4

Dre_fgf20a:ENSDARG00000007356:ENSDART00000013024.8

Dre_fgf20b:ENSDARG00000086070:ENSDART00000131174.3

Dre_fgf21:ENSDARG00000100846:ENSDART00000164087.1

Dre_fgf22:ENSDARG00000076510:ENSDART00000109655.3

Dre_fgf23:ENSDARG00000045854:ENSDART00000067388.3

Dre_fgf24:ENSDARG00000037677:ENSDART00000054877.7

Dre_fgf2:ENSDARG00000104348:ENSDART00000168951.2

Dre_fgf3:ENSDARG00000101540:ENSDART00000168474.2

Dre_fgf4:ENSDARG00000105230:ENSDART00000158898.2

Dre_fgf5:ENSDARG00000035377:ENSDART00000051256.4

Dre_fgf6a:ENSDARG00000009351:ENSDART00000016591.4

Dre_fgf6b_nextfgf23:ENSDARG00000045856:ENSDART00000067390.5

Dre_fgf7:ENSDARG00000059387:ENSDART00000139752.2

Dre_fgf8a:ENSDARG00000003399:ENSDART00000025583.7

Dre_fgf8b:ENSDARG00000039615:ENSDART00000057885.8

Dre_fgf9:ENSDARG00000078625:ENSDART00000108697.4

Gac_12bl:ENSGACG00000004403:ENSGACT00000005825.1

Gac_13b:ENSGACG00000020450:ENSGACT00000027095.1

Gac_fgf10a:ENSGACG00000017290:ENSGACT00000022877.1

Gac_fgf10b:ENSGACG00000004522:ENSGACT00000005983.1

Gac_fgf11a:ENSGACG00000019942:ENSGACT00000026395.1

Gac_fgf11b:ENSGACG00000020321:ENSGACT00000026901.1

Gac_fgf12a:ENSGACG00000013900:ENSGACT00000018405.1

Gac_fgf13:ENSGACG00000017204:ENSGACT00000022768.1

Gac_fgf14:ENSGACG00000001948:ENSGACT00000002545.1

Gac_fgf16:ENSGACG00000018221:ENSGACT00000024137.1

Gac_fgf17:ENSGACG00000003432:ENSGACT00000004497.1

Gac_fgf18a:ENSGACG00000017921:ENSGACT00000023719.1

Gac_fgf19:ENSGACG00000014191:ENSGACT00000018770.1

Gac_fgf1a:ENSGACG00000018207:ENSGACT00000024118.1

Gac_fgf1b:ENSGACG00000020727:ENSGACT00000027455.1

Gac_fgf1b:ENSGACG00000020727:ENSGACT00000027456.1

Gac_fgf20b:ENSGACG00000018613:ENSGACT00000024653.1

Gac_fgf23:ENSGACG00000019905:ENSGACT00000026347.1

Gac_fgf24:ENSGACG00000016697:ENSGACT00000022087.1

Gac_fgf2:ENSGACG00000016721:ENSGACT00000022120.1

Gac_fgf3:ENSGACG00000014186:ENSGACT00000018761.1

Gac_fgf4:ENSGACG00000014187:ENSGACT00000018768.1

Gac_fgf5:ENSGACG00000000178:ENSGACT00000000232.1

Gac_fgf6_nextfgf23:ENSGACG00000019904:ENSGACT00000026345.1

Gac_fgf6:ENSGACG00000013812:ENSGACT00000018274.1

Gac_fgf7:ENSGACG00000016807:ENSGACT00000022229.1

Gac_fgf8a:ENSGACG00000003803:ENSGACT00000005026.1

Gac_fgf8b:ENSGACG00000018276:ENSGACT00000024207.1

Gac_fgf9:ENSGACG00000001462:ENSGACT00000001894.1

fgf10a_KV879994.1:ENSHCOG00000003348:ENSHCOT00000010720.1

fgf10b_KV879773.1:ENSHCOG00000006595:ENSHCOT00000006105.1

fgf11a_KV879891.1:ENSHCOG00000010205:ENSHCOT00000000983.1

fgf11b_KV879794.1:ENSHCOG00000001048:ENSHCOT00000014052.1

fgf12a_KV880460.1:ENSHCOG00000020070:ENSHCOT00000028443.1

fgf12b_KV880533.1:ENSHCOG00000019239:ENSHCOT00000023550.1

fgf13b_KV880343.1:ENSHCOG00000009068:ENSHCOT00000002517.1

fgf13_KV879805.1:ENSHCOG00000001163:ENSHCOT00000013894.1

fgf14_KV880385.1:ENSHCOG00000003089:ENSHCOT00000011122.1

fgf14like_KV880103.1:ENSHCOG00000009194:ENSHCOT00000002338.1

fgf16_KV879952.1:ENSHCOG00000005892:ENSHCOT00000007059.1

fgf17_KV880531.1:ENSHCOG00000001834:ENSHCOT00000012937.1

fgf18a_KV880056.1:ENSHCOG00000014470:ENSHCOT00000026633.1

fgf19_KV880514.1:ENSHCOG00000007403:ENSHCOT00000004893.1

fgf1a_KV879952.1:ENSHCOG00000005595:ENSHCOT00000007481.1

fgf1a_KV879952.1:ENSHCOG00000005595:ENSHCOT00000007539.1

fgf1alike_KV880017.1:ENSHCOG00000016029:ENSHCOT00000019906.1

fgf1alike_KV880017.1:ENSHCOG00000016029:ENSHCOT00000027062.1

fgf20a_KV880079.1:ENSHCOG00000020837:ENSHCOT00000025270.1

fgf20b_KV880218.1:ENSHCOG00000004017:ENSHCOT00000009816.1

fgf21like_KV880215.1:ENSHCOG00000018139:ENSHCOT00000027788.1

fgf22_KV880451.1:ENSHCOG00000001540:ENSHCOT00000013363.1

fgf23_KV880389.1:ENSHCOG00000017488:ENSHCOT00000021721.1

fgf24_KV880347.1:ENSHCOG00000003883:ENSHCOT00000009996.1

fgf2_KV880347.1:ENSHCOG00000004057:ENSHCOT00000009763.1

fgf2_KV880347.1:ENSHCOG00000004057:ENSHCOT00000009785.1

fgf4like_KV880156.1:ENSHCOG00000013232:ENSHCOT00000026245.1

fgf5_KV880271.1:ENSHCOG00000009271:ENSHCOT00000002212.1

fgf6_KV880265.1:ENSHCOG00000012911:ENSHCOT00000016620.1

fgf6_nextfg23_KV880389.1:ENSHCOG00000017469:ENSHCOT00000021674.1

fgf7_KV880248.1:ENSHCOG00000001786:ENSHCOT00000013001.1

fgf8a_KV880188.1:ENSHCOG00000009341:ENSHCOT00000002151.1

fgf8b_KV880294.1:ENSHCOG00000015697:ENSHCOT00000019757.1

Loc_fgf10b:ENSLOCG00000012296:ENSLOCT00000015159.1

Loc_fgf11a:ENSLOCG00000013755:ENSLOCT00000016994.1

Loc_fgf12a:ENSLOCG00000009371:ENSLOCT00000011445.1

Loc_fgf13:ENSLOCG00000012296:ENSLOCT00000015159.1

Loc_fgf14:ENSLOCG00000002765:ENSLOCT00000003264.1

Loc_fgf16:ENSLOCG00000013663:ENSLOCT00000016875.1

Loc_fgf17:ENSLOCG00000014874:ENSLOCT00000018351.1

Loc_fgf18b:ENSLOCG00000009693:ENSLOCT00000011857.1

Loc_fgf19:ENSLOCG00000005100:ENSLOCT00000006148.1

Loc_fgf1:ENSLOCG00000010543:ENSLOCT00000012944.1

Loc_fgf20a:ENSLOCG00000013962:ENSLOCT00000017244.1

Loc_fgf22:ENSLOCG00000005240:ENSLOCT00000006319.1

Loc_fgf23:ENSLOCG00000016671:ENSLOCT00000020629.1

Loc_fgf24:ENSLOCG00000001921:ENSLOCT00000002231.1

Loc_fgf2:ENSLOCG00000002819:ENSLOCT00000003323.1

Loc_fgf3:ENSLOCG00000005127:ENSLOCT00000006178.1

Loc_fgf4:ENSLOCG00000005119:ENSLOCT00000006170.1

Loc_fgf5:ENSLOCG00000007208:ENSLOCT00000008732.1

Loc_fgf6b:ENSLOCG00000016670:ENSLOCT00000020628.1

Loc_fgf7:ENSLOCG00000013162:ENSLOCT00000016244.1

Loc_fgf8b:ENSLOCG00000011679:ENSLOCT00000014381.1

Loc_fgf9_LG17_fgf4like:ENSLOCG00000003820:ENSLOCT00000004564.1

Loc_fgf9_LG3:ENSLOCG00000001730:ENSLOCT00000002001.1

OlaHSOK_fgf10a:ENSORLG00015002082:ENSORLT00015012577.1

OlaHSOK_fgf10b:ENSORLG00015016632:ENSORLT00015034007.1

OlaHSOK_fgf11a:ENSORLG00015015483:ENSORLT00015022192.1

OlaHSOK_fgf11b:ENSORLG00015003409:ENSORLT00015010492.1

OlaHSOK_fgf12a:ENSORLG00015014441:ENSORLT00015020965.1

OlaHSOK_fgf12b:ENSORLG00015011383:ENSORLT00015017181.1

OlaHSOK_fgf13b:ENSORLG00015002662:ENSORLT00015011665.1

OlaHSOK_fgf13:ENSORLG00015010973:ENSORLT00015016615.1

OlaHSOK_fgf14:ENSORLG00015017262:ENSORLT00015024434.1

OlaHSOK_fgf16:ENSORLG00015003530:ENSORLT00015010311.1

OlaHSOK_fgf18:ENSORLG00015021415:ENSORLT00015029455.1

OlaHSOK_fgf19:ENSORLG00015005490:ENSORLT00015007126.1

OlaHSOK_fgf1a:ENSORLG00015003327:ENSORLT00015010606.1

OlaHSOK_fgf1a:ENSORLG00015003327:ENSORLT00015010610.1

OlaHSOK_fgf20a:ENSORLG00015000486:ENSORLT00015024745.1

OlaHSOK_fgf20b:ENSORLG00015013659:ENSORLT00015020002.1

OlaHSOK_fgf22:ENSORLG00015022219:ENSORLT00015030385.1

OlaHSOK_fgf23:ENSORLG00015010012:ENSORLT00015015473.1

OlaHSOK_fgf24:ENSORLG00015021658:ENSORLT00015029725.1

OlaHSOK_fgf2:ENSORLG00015022023:ENSORLT00015030147.1

OlaHSOK_fgf3:ENSORLG00015005472:ENSORLT00015007174.1

OlaHSOK_fgf4:ENSORLG00015000158:ENSORLT00015007146.1

OlaHSOK_fgf5:ENSORLG00015001741:ENSORLT00015013130.1

OlaHSOK_fgf6_nextfgf23:ENSORLG00015000683:ENSORLT00015015478.1

OlaHSOK_fgf6:ENSORLG00015002360:ENSORLT00015012263.1

OlaHSOK_fgf7:ENSORLG00015021441:ENSORLT00015029500.1

OlaHSOK_fgf8a:ENSORLG00015011045:ENSORLT00015016748.1

OlaHSOK_fgf8b:ENSORLG00015001891:ENSORLT00015012891.1

OlaHSOK_fgf9:ENSORLG00015003864:ENSORLT00015009766.1

Xma_fgf10a:ENSXMAG00000023995:ENSXMAT00000040231.1

Xma_fgf10a:ENSXMAG00000023995:ENSXMAT00000042112.1

Xma_fgf10b:ENSXMAG00000010136:ENSXMAT00000010170.2

Xma_fgf11a:ENSXMAG00000013668:ENSXMAT00000013710.2

Xma_fgf11b:ENSXMAG00000000385:ENSXMAT00000000384.2

Xma_fgf12a:ENSXMAG00000015648:ENSXMAT00000015704.2

Xma_fgf12b:ENSXMAG00000014755:ENSXMAT00000014818.2

Xma_fgf13b:ENSXMAG00000000441:ENSXMAT00000000437.2

Xma_fgf13:ENSXMAG00000022835:ENSXMAT00000022378.1

Xma_fgf14:ENSXMAG00000021156:ENSXMAT00000023347.1

Xma_fgf16:ENSXMAG00000026062:ENSXMAT00000034324.1

Xma_fgf16:ENSXMAG00000026062:ENSXMAT00000035274.1

Xma_fgf17:ENSXMAG00000003974:ENSXMAT00000040102.1

Xma_fgf18a:ENSXMAG00000008345:ENSXMAT00000008382.2

Xma_fgf19:ENSXMAG00000001523:ENSXMAT00000001519.2

Xma_fgf1a:ENSXMAG00000028252:ENSXMAT00000031984.1

Xma_fgf1l:ENSXMAG00000001555:ENSXMAT00000001550.2

Xma_fgf20a:ENSXMAG00000024245:ENSXMAT00000030065.1

Xma_fgf20b:ENSXMAG00000017419:ENSXMAT00000017478.2

Xma_fgf21:ENSXMAG00000001586:ENSXMAT00000041881.1

Xma_fgf21:ENSXMAG00000001586:ENSXMAT00000001579.2

Xma_fgf22:ENSXMAG00000012475:ENSXMAT00000012508.2

Xma_fgf23:ENSXMAG00000004291:ENSXMAT00000038648.1

Xma_fgf23:ENSXMAG00000004291:ENSXMAT00000004303.2

Xma_fgf24:ENSXMAG00000001991:ENSXMAT00000001993.2

Xma_fgf2:ENSXMAG00000024887:ENSXMAT00000031593.1

Xma_fgf3:ENSXMAG00000001504:ENSXMAT00000001504.2

Xma_fgf4:ENSXMAG00000026924:ENSXMAT00000037002.1

Xma_fgf5:ENSXMAG00000022690:ENSXMAT00000037530.1

Xma_fgf6_next23:ENSXMAG00000029319:ENSXMAT00000023079.1

Xma_fgf6:ENSXMAG00000004288:ENSXMAT00000004300.2

Xma_fgf7:ENSXMAG00000002280:ENSXMAT00000002286.2

Xma_fgf8a:ENSXMAG00000025032:ENSXMAT00000021323.1

Xma_fgf8b:ENSXMAG00000021661:ENSXMAT00000032206.1

Protein-coding DNA sequences for PAML analyses

fgf4-like

Bpe XM_020919979.1

Hco ENSHCOG00000013232.1:ENSHCOT00000026245.1

Tor hand annotated from BKCK01000126.1

Oni ENSONIG00000017466.2:ENSONIT00000022045.2

Xma ENSXMAG00000008556:ENSXMAT00000036490.1

Fgf5

Tru ENSTRUG00000000972:ENSTRUT00000002303.3

Hco ENSHCOG00000009271:ENSHCOT00000002212.1

Ola ENSORLG00015001741:ENSORLT00015013130.1

Oni ENSONIG00000033859:ENSONIT00000071224.1

Pma ENSPMGG00000020987:ENSPMGT00000027719.1

Xma ENSXMAG00000022690:ENSXMAT00000037530.1

fgf6a

Bpe XM_020926718.1

Tru ENSTRUG00000011078.3:ENSTRUT00000028081.3

Hco XM_019893705.1

Ola ENSORLG00015000683.1:ENSORLT00015015509.1

Oni ENSONIG00000035736.1:ENSONIT00000088507.1

Sac XM_037243485.1

Xma fgf6a ENSXMAG00000029319.1:ENSXMAT00000023079.1

Fgf6b

Bpe XM_020935296.1

Tru ENSTRUG00000010251.3:ENSTRUT00000025921.3

Hco ENSHCOG00000012911.1:ENSHCOT00000016620.1

Ola ENSORLG00015002360.1:ENSORLT00015012287.1

Oni ENSONIG00000041125.1:ENSONIT00000067386.1

Sac mod from XM_037253970.1 (minus last AAs: LPPPSKDRQ)

Xma ENSXMAG00000004288:ENSXMAT00000004300.2

fgf7

Bme XM_020939054.1

Tru ENSTRUG00000021359.2:ENSTRUT00000052133.2

Hco ENSHCOG00000001786.1:ENSHCOT00000013001.1

Ola ENSORLG00015021441.1:ENSORLT00015029500.1

Oni ENSONIG00000013338.2:ENSONIT00000016787.2

Xma ENSXMAG00000002280.2:ENSXMAT00000002286.2

fgf8a

Bpe XM_020921293.1

Tru ENSTRUG00000000925.3:ENSTRUT00000081859.1

Hco XM_019881874.1

Ola ENSORLG00015011045.1:ENSORLT00015016748.1

Oni ENSONIG00000013179.2:ENSONIT00000016589.2

Sac XM_037270980.1

Xma ENSXMAG00000025032.1:ENSXMAT00000026790.1

fgf8b

Bpe mod from XM_020941882.1

(5'extensionfromScaffNW_018348411.1)

Tru ENSTRUG00000022621.2:ENSTRUT00000050186.2

Hco XM_019889057.1

Ola ENSORLG00015001891.1:ENSORLT00015012900.1

Oni ENSONIG00000031278.1:ENSONIT00000058602.1

Sac XM_037245726.1

Tal hand annotated from OMLG01003852

Dda hand annotated from CAAKHH010016828

Xma ENSXMAG00000021661.1:ENSXMAT00000032206.1

fgf10a

Bpe XM_020940229.1

Tru ENSTRUG00000023059.2:ENSTRUT00000049927.2

Hco ENSHCOG00000003348.1:ENSHCOT00000010720.1

Ola ENSORLG00015002082.1:ENSORLT00015012577.1

Oni ENSONIG00000003669.2:ENSONIT00000004619.2

Sac XM_037266657.1

Xma ENSXMAG00000023995.1:ENSXMAT00000042112.1

fgf10b

Bpe XM_020918000.1

Tru ENSTRUG00000016593.3:ENSTRUT00000042577.3

Hco ENSHCOG00000006595.1:ENSHCOT00000006129.1

Ola ENSORLG00015016632.1:ENSORLT00015034010.1

Oni ENSONIG00000043120.1:ENSONIT00000090719.1

Sac XM_037259188.1

Xma fgf10b_>ENSXMAG00000010136.2:ENSXMAT00000010170.2

fgf16

Bpe XM_020928947.1

Tru ENSTRUG00000022728.2:ENSTRUT00000049290.2

Hco ENSHCOG00000005892.1:ENSHCOT00000007059.1

(mod. with genomic seq)

Ola ENSORLG00015003530.1:ENSORLT00015010311.1

Oni ENSONIG00000017896.2:ENSONIT00000022574.2

Sac modified from XM_037262282.1

Xma XM_005795490.3

Additional seqs used in Fgf16 protein alignment figure but not PAML

Gga ENSGALG00000007806.5: ENSGALT00000012662.5

Hsa ENSG00000196468.8:ENST00000439435.3

fgf17

Bpe XM_020918675.1

Tru XM_029830305.1

Hco XM_019860861.1

Oni ENSONIG00000020227.2:ENSONIT00000025507.2

Sac hand annotated from LR594594.1

Xma XM_032577758.1

fgf19

Bpe XM_020940642.1

Tru ENSTRUG00000029764.1:ENSTRUT00000083421.1

Hco ENSHCOG00000007403.1:ENSHCOT00000004893.1

Oni ENSONIG00000018094.2:ENSONIT00000022816.2

Ola ENSORLG00015005490.1:ENSORLT00015007126.1

Sac XM_037246711.1

Tal hand annotated from OMLG01037534.1

Xma ENSXMAG00000001523.2:ENSXMAT00000001519.2

fgf20a

Bpe XM_020931495.1

Tru ENSTRUG00000015359.3:ENSTRUT00000039390.3

Hco XM_019876469.1

Ola ENSORLG00015000486.1:ENSORLT00015024745.1

Oni ENSONIG00000016247.2:ENSONIT00000020472.2

Sac XM_037260044.1

Xma ENSXMAG00000024245.1:ENSXMAT00000030065.1

fgf20b

Bpe XM_020917885.1

Tru ENSTRUG00000003722.3:ENSTRUT00000008788.3

Hco ENSHCOG00000004017.1:ENSHCOT00000009816.1

Ola ENSORLG00015013659.1:ENSORLT00015020002.1

Oni fgf20b_ENSONIG00000017603.2:ENSONIT00000022215.2

Sac XM_037262485.1

Xma ENSXMAG00000017419.2:ENSXMAT00000017478.2

fgf22

Bpe XM_020940148.1

Tru ENSTRUG00000020363.2:ENSTRUT00000054966.2

Hco ENSHCOG00000001540.1:ENSHCOT00000013363.1

Ola ENSORLG00015022219.1:ENSORLT00015030385.1

Oni ENSONIG00000002404.2:ENSONIT00000003007.2

Sac XM_037250274.1

Xma ENSXMAG00000012475.2:ENSXMAT00000012508.2

fgf24

Bpe XM_020917646.1

Tru ENSTRUG00000008042.3:ENSTRUT00000054629.2

Hco XM_019892219.1

Ola ENSORLG00015021658.1:ENSORLT00015029732.1

Oni ENSONIG00000001385.2:ENSONIT00000001747.2

Xma ENSXMAG00000001991.2:ENSXMAT00000001993.2

fgfr1a

Bpe XM_020928300.1

Tru ENSTRUG00000016527: ENSTRUT00000077550.1

Oni ENSONIG00000011012: ENSONIT00000055055.1

Hco ENSHCOG00000000677

Ola ENSORLG00015015014: ENSORLT00015021156.1

Xma ENSXMAG00000028744

fgfr1b

Bpe XM_020926403.1

Tru ENSTRUG00000018627

Oni ENSONIG00000012609: ENSONIT00000076591.1

Hco ENSHCOG00000012193 (patched with sequence from KV880234.1)

Ola ENSORLG00015005324: ENSORLT00015007402.1

Xma ENSXMAG00000016402: ENSXMAT00000016515.2

fgfr2

Bpe XM_020933558.1

Tru ENSTRUG00000017610: ENSTRUT00000045297.3

Ola ENSORLG00015021373: ENSORLT00015029412.1

Xma ENSXMAG00000009512: ENSXMAT00000028926.1

Hco ENSHCOG00000003717

Oni ENSONIG00000010192: ENSONIT00000055812.1

fgfr3

Bpe XM_020919786

Tru ENSTRUG00000003670: ENSTRUT00000072878.1

Ola ENSORLG00015009276: ENSORLT00015000797.1

Xma ENSXMAG00000026115: ENSXMAT00000036089.1

Hco ENSHCOG00000001033: ENSHCOT00000014086.1

Oni ENSONIG00000012350: ENSONIT00000063330.1

fgfr4

Bpe XM_020920666.1

Ola ENSORLG00015018433:

Hco ENSHCOG00000010690

Oni ENSONIG00000017319: ENSONIT00000021862.2

Xma ENSXMAG00000018788

Tru ENSTRUG00000004233: ENSTRUT00000010086.3

fgfrl1a

Bpe XM_020923464

Tru ENSTRUG00000015192

Ola ENSORLG00015016006

Xma ENSXMAG00000027004: ENSXMAT00000030218.1

Hco ENSHCOG00000007058: ENSHCOT00000005452.1

Oni ENSONIG00000001038

fgfrl1b

Bpe XM_020919814.1

Tru ENSTRUG00000008594: ENSTRUT00000060890.1

Ola ENSORLG00015008260: ENSORLT00015002533.1

Xma ENSXMAG00000013536: ENSXMAT00000013578.2

Hco ENSHCOG00000020680: ENSHCOT00000028653.1

Oni ENSONIG00000042768

REFERENCES

1. M. Kolmogorov, J. Yuan, Y. Lin, P. A. Pevzner, Assembly of long, error-prone reads using repeat graphs. *Nat Biotechnol* **37**, 540-546 (2019).
2. C. S. Chin *et al.*, Nonhybrid, finished microbial genome assemblies from long-read SMRT sequencing data. *Nat Methods* **10**, 563-569 (2013).
3. H. Li, Minimap2: pairwise alignment for nucleotide sequences. *Bioinformatics* **34**, 3094-3100 (2018).
4. B. J. Walker *et al.*, Pilon: an integrated tool for comprehensive microbial variant detection and genome assembly improvement. *PLoS One* **9**, e112963 (2014).
5. H. Li, Aligning sequence reads, clone sequences and assembly contigs with BWA-MEM. *arXiv* (2013).
6. G. W. Vurture *et al.*, GenomeScope: fast reference-free genome profiling from short reads. *Bioinformatics* **33**, 2202-2204 (2017).
7. O. Dudchenko *et al.*, 10.1101/254797 (2018).
8. O. Dudchenko *et al.*, De novo assembly of the Aedes aegypti genome using Hi-C yields chromosome-length scaffolds. *Science* **356**, 92-95 (2017).
9. N. C. Durand *et al.*, Juicebox Provides a Visualization System for Hi-C Contact Maps with Unlimited Zoom. *Cell Syst* **3**, 99-101 (2016).
10. C. M. Small, K. Milligan-Myhre, S. Bassham, K. Guillemin, W. A. Cresko, Host Genotype and Microbiota Contribute Asymmetrically to Transcriptional Variation in the Threespine Stickleback Gut. *Genome Biol Evol* **9**, 504-520 (2017).
11. J. Catchen, P. A. Hohenlohe, S. Bassham, A. Amores, W. A. Cresko, Stacks: an analysis tool set for population genomics. *Mol Ecol* **22**, 3124-3140 (2013).
12. J. M. Catchen, A. Amores, P. Hohenlohe, W. Cresko, J. H. Postlethwait, Stacks: building and genotyping Loci de novo from short-read sequences. *G3 (Bethesda)* **1**, 171-182 (2011).
13. M. Martin, Cutadapt removes adapter sequences from high-throughput sequencing reads. *EMBnet.journal* **17**, 10-12 (2011).
14. B. Bushnell, J. Rood, E. Singer, BBMerge - Accurate paired shotgun read merging via overlap. *PLoS One* **12**, e0185056 (2017).
15. B. Bushnell, BBMap: A fast, accurate, splice-aware aligner. *sourceforge.net/projects/bbmap/* (2014).
16. T. Desvignes, P. Batzel, J. Sydes, B. F. Eames, J. H. Postlethwait, miRNA analysis with Prost! reveals evolutionary conservation of organ-enriched expression and post-transcriptional modifications in three-spined stickleback and zebrafish. *Sci Rep* **9**, 3913 (2019).
17. A. Dobin *et al.*, STAR: ultrafast universal RNA-seq aligner. *Bioinformatics* **29**, 15-21 (2013).
18. A. F. A. Smit, R. Hubley, P. Green, RepeatMasker at http://repeatmasker.org.
19. J. M. Flynn *et al.*, RepeatModeler2 for automated genomic discovery of transposable element families. *Proc Natl Acad Sci U S A* **117**, 9451-9457 (2020).
20. T. Bruna, K. J. Hoff, A. Lomsadze, M. Stanke, M. Borodovsky, BRAKER2: automatic eukaryotic genome annotation with GeneMark-EP+ and AUGUSTUS supported by a protein database. *NAR Genom Bioinform* **3**, lqaa108 (2021).
21. P. Jones *et al.*, InterProScan 5: genome-scale protein function classification. *Bioinformatics* **30**, 1236-1240 (2014).

22. S. F. Altschul, W. Gish, W. Miller, E. W. Myers, D. J. Lipman, Basic local alignment search tool. *J Mol Biol* **215**, 403-410 (1990).
23. A. J. Enright, S. Van Dongen, C. A. Ouzounis, An efficient algorithm for large-scale detection of protein families. *Nucleic Acids Res* **30**, 1575-1584 (2002).
24. F. K. Mendes, D. Vanderpool, B. Fulton, M. W. Hahn, CAFE 5 models variation in evolutionary rates among gene families. *Bioinformatics* 10.1093/bioinformatics/btaa1022 (2020).
25. D. L. Rabosky *et al.*, An inverse latitudinal gradient in speciation rate for marine fishes. *Nature* **559**, 392-395 (2018).
26. G. Yu, L. G. Wang, Y. Han, Q. Y. He, clusterProfiler: an R package for comparing biological themes among gene clusters. *OMICS* **16**, 284-287 (2012).
27. Y. Moriya, M. Itoh, S. Okuda, A. C. Yoshizawa, M. Kanehisa, KAAS: an automatic genome annotation and pathway reconstruction server. *Nucleic Acids Res* **35**, W182-185 (2007).
28. B. J. Haas, TransposonPSI. *http://transposonpsi.sourceforge.net.*
29. K. Katoh, D. M. Standley, MAFFT multiple sequence alignment software version 7: improvements in performance and usability. *Mol Biol Evol* **30**, 772-780 (2013).
30. T. J. Wheeler, "Large-scale neighbor-joining with NINJA" in Algorithms in Bioinformatics. Lecture Notes in Computer Science, S. L. Salzberg, T. Warnow, Eds. (Springer Berlin, Heidelberg, 2009).
31. F. Shao, J. Wang, H. Xu, Z. Peng, FishTEDB: a collective database of transposable elements identified in the complete genomes of fish. *Database (Oxford)* **2018** (2018).
32. R. C. Edgar, Search and clustering orders of magnitude faster than BLAST. *Bioinformatics* **26**, 2460-2461 (2010).
33. J. Zhang, R. Nielsen, Z. Yang, Evaluation of an improved branch-site likelihood method for detecting positive selection at the molecular level. *Mol Biol Evol* **22**, 2472-2479 (2005).
34. S. J. Longo *et al.*, Phylogenomic analysis of a rapid radiation of misfit fishes (Syngnathiformes) using ultraconserved elements. *Mol Phylogenet Evol* **113**, 33-48 (2017).
